# *Klebsiella*-dominant gut communities are associated with impeded oral rotavirus vaccine response in infants

**DOI:** 10.1038/s41467-026-78148-x

**Published:** 2026-10-15

**Authors:** Cale O. Seymour, Gertrude Ecklu-Mensah, Yesun Lee, Alexander R. Nicols, Wesley A. Cheng, Jaycee M. Jumarang, Carolyn Jennifer Marentes Ruiz, Jocelyn Navarro, Rafaella Navarro, Yamile Rodriguez-Angeles, David Durand, Sarah M. Allard, Zackary W. Taylor, Ruth G. De León, Theresa J. Ochoa, Jack A. Gilbert, Pia S. Pannaraj

**Affiliations:** 1 Department of Pediatrics, University of California San Diego, La Jolla, CA USA; 2 Scripps Institution of Oceanography, University of California San Diego, La Jolla, CA USA; 3 Instituto de Medicina Tropical Alexander von Humboldt and Laboratorios de Investigación y Desarrollo, Universidad Peruana Cayetano Heredia, Lima, Peru; 4 Department of Pediatrics, Kaiser Permanente, Los Angeles, CA USA; 5 Department of Sexual and Reproductive Health, Gorgas Memorial Institute for Health Studies, Panama City, Panama; 6 Division of Infectious Diseases, Rady Children’s Health, San Diego, CA USA

**Keywords:** Viral infection, Paediatric research

## Abstract

Rotavirus gastroenteritis is the leading cause of infant diarrheal death worldwide. Oral rotavirus vaccine (RVV) prevents illness and death, but efficacy differs between high- and low-income countries and the etiologies are unclear. Here, we describe the relationship between colonization of the infant gut by commensal bacteria, inflammation, and vaccine response across a cohort of infants from Panama, Peru, and the U.S. Vaccine immunogenicity was related to community composition in the first month of life and inverse to increased relative abundance of *Klebsiella*. Clustering communities into gut types revealed that microbiome configurations with elevated *Klebsiella* or *Escherichia* in the first month of life were negatively associated with both seroconversion and rotavirus shedding after vaccination, suggesting potential inhibition of vaccine function or host response. Infants without RVV strain shedding displayed elevated fecal calprotectin in the first month. These results implicate microbiome-augmented infant gut inflammation as a potential impediment to RVV response.

## Abstract (web summary)

Here, the authors identify a relationship between rotavirus vaccine response, colonization of the infant gut by commensal bacteria, and inflammation across a cohort of infants from three different regions (Panama, Peru, and the U.S.A).’

## Introduction

Rotavirus remains the leading cause of diarrheal mortality among children under 5 years of age globally^1–3^. Introduction of oral, live, attenuated rotavirus vaccines (RVVs) has significantly reduced the burden of rotavirus gastroenteritis. However, the effectiveness of RVV is markedly reduced in low- and middle-income countries (LMICs)^4^, where disease burden is the highest^5,6^. The biological mechanisms underlying this discrepancy are incompletely understood, and identifying the drivers of this efficacy gap is a critical public health priority.

Multiple population- and individual-level factors have been proposed to influence oral vaccine performance, including maternal secretor status and breastfeeding^7,8^, pre-existing immunity^8,9^, antibiotic usage^10^, environmental enteropathy, and intestinal dysbiosis^6,8,11–15^. Among these, composition and function of the early-life gut microbiome are potentially modifiable determinants of RVV response. Infant gut microbiota plays a key role in shaping mucosal immune development, antigen uptake, inflammatory tone, and B cell-mediated IgA responses. Recent studies suggest that the structure of the infant gut microbiome may alter RVV immunogenicity, although results have varied across settings. Nicaraguan infants who failed to seroconvert showed elevated Enterobacteriaceae and depleted *Bacteroides*^16^, while infants responders had decreased Enterobacteriaceae in early life^11^. Ghanaian infants whose gut microbiota resembled that of Dutch infants exhibited greater IgA fold change following vaccination^13^. In contrast, Indian infants with lower gut microbial richness demonstrated enhanced vaccine immunogenicity^8,14^. Additional studies in India and Malawi link RVV strain shedding dynamics to the relative abundance of Enterobacteriaceae and *Collinsella*^15^. While these findings highlight potential microbial signatures of vaccine responsiveness, heterogeneity in study design, including differences in microbiome profiling techniques, dosing schedules, and infant age, complicates interpretation. Moreover, the ecological configurations and metabolic capacities of gut microbes that consistently promote or impair RVV efficacy remain poorly defined.

Few studies have integrated microbial, inflammatory, and immunological data in a longitudinal framework across geographically distinct populations. Here, we address this critical gap through a longitudinal, multi-country infant cohort study with integration of high-resolution gut microbiome profiling with clinical measures of RVV response and gut inflammation to define microbial-inflammatory-immune axes that modulate vaccine efficacy.

We identify *Klebsiella*- and *Escherichia*-dominated gut communities as being associated with impaired seroconversion and reduced RVV strain shedding, likely mediated by microbiota-driven mucosal inflammation. These findings underscore the importance of early-life microbial colonization in shaping oral vaccine outcomes and suggest that microbiome-informed interventions may improve vaccine performance in high-risk populations.

## Results

### Description of cohort structure and rotavirus vaccine responses

**Fig. 1:**
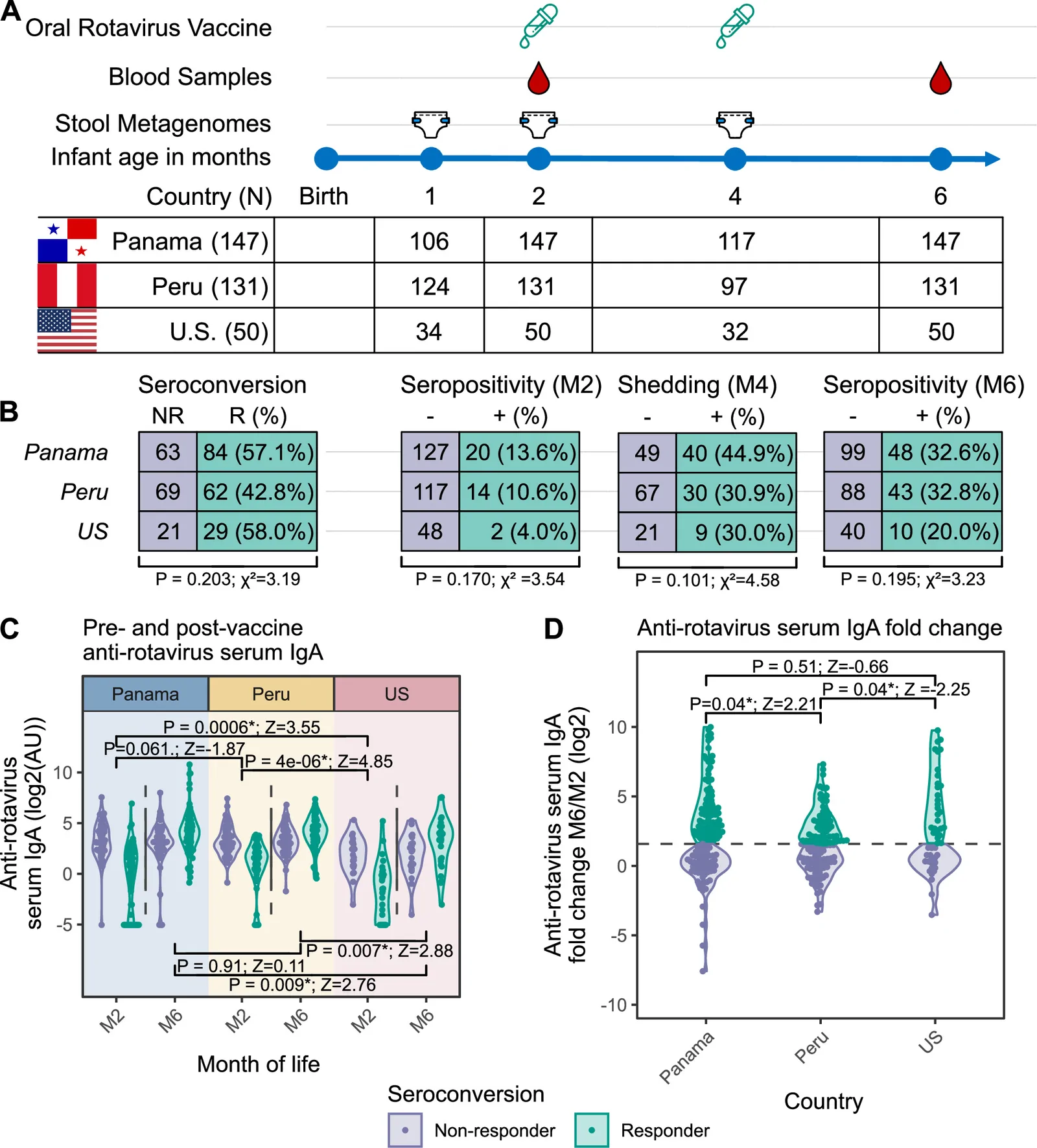
Study overview and Rotavirus Vaccination 1 (RV1) response statistics. **A** Summary of enrollment, infant RV1 vaccine schedule, and specimen collection timeline. **B** Number of infants that displayed each RV1 seroconversion and shedding outcome per country; none of these comparisons differed by country. **C** Distribution of paired pre- (M2) and post-vaccine (M6) anti-rotavirus IgA magnitude in each country. *P*-values indicate differences in anti-rotavirus IgA between countries at each time point and were calculated via Dunn’s test (*N* = 328 infants). **D** Distribution of anti-rotavirus IgA fold change from pre- to post-vaccination, grouped by RV1 response. *P*-values indicate differences in anti-rotavirus IgA fold change between countries and were calculated via Dunn’s test (*N* = 328) with BH correction. Additional analyses of RV1 response dynamics are available in Supplementary Fig. 3.

We enrolled 337 healthy infants shortly after birth who completed the 2-dose RVV (RV1, Rotarix, GlaxoSmithKline) series at 2 and 4 months of age in Panama, Peru, and the U.S. Of those, 328 (97.3%) completed visits through 6 months of age (Fig. 1A). We tracked demographic, clinical, and dietary factors at each visit (Table 1). To estimate vaccine response, we utilized two response measures: RV-IgA seroconversion and RV1 stool shedding. Seroconversion was defined as a ≥3-fold increase in serum rotavirus-specific IgA from pre-vaccine at month 2 (M2) (median: 54 days of life [DOL]) to post-vaccination at month 6 (M6) (median: 188 DOL). To complement rotavirus IgA serology, we assayed the shedding of vaccine-strain RNA via polymerase chain reaction (PCR) in stool within 14 days after the second dose of RV1 at month 4 (M4) (median: 132 DOL).

**Table 1:** Demographic and clinical characteristics of mother–infant pairs

	Overall	Panama	Peru	U.S.	*P*-Value
	(*N* = 328)	(*N* = 147)	(*N* = 131)	(*N* = 50)	
Male sex	157 (47.9%)	76 (51.7%)	54 (41.2%)	27 (54.0%)	0.14
Mother age (years)					
Mean [SD]	28.5 [6.11]	26.5 [5.37]	28.1 [5.72]	35.0 [4.49]	<0.001
Maternal secretor status					
Secretor	286 (87.2%)	128 (87.1%)	124 (94.7%)	34 (68.0%)	<0.001
Non-secretor	27 (8.2%)	12 (8.2%)	3 (2.3%)	12 (24.0%)	
Inconclusive	4 (1.2%)	0 (0%)	4 (3.1%)	0 (0%)	
Hispanic ethnicity	289 (88.1%)	137 (93.2%)	131 (100%)	21 (42.0%)	<0.001
Race					
American Indian	10 (3.0%)	10 (6.8%)	0 (0%)	0 (0%)	<0.001
Asian	8 (2.4%)	0 (0%)	0 (0%)	8 (16.0%)	
Black	26 (7.9%)	22 (15.0%)	0 (0%)	4 (8.0%)	
White	116 (35.4%)	85 (57.8%)	0 (0%)	31 (62.0%)	
Other	1 (0.3%)	1 (0.7%)	0 (0%)	0 (0%)	
Mixed	167 (50.9%)	29 (19.7%)	131 (100%)	7 (14.0%)	
Delivery Type					
Vaginal	271 (82.6%)	118 (80.3%)	122 (93.1%)	31 (62.0%)	<0.001
C-section	57 (17.4%)	29 (19.7%)	9 (6.9%)	19 (38.0%)	
Gestational age (weeks)					
Mean [SD]	39.0 [1.20]	38.9 [1.23]	38.8 [1.07]	39.5 [1.29]	0.001
Birth weight (grams)					
Mean [SD]	3.29 [0.406]	3.25 [0.408]	3.30 [0.386]	3.40 [0.439]	0.092
Diet at RV 1st dose					
Human milk	207 (63.1%)	75 (51.0%)	105 (80.2%)	27 (54.0%)	<0.001
Formula	4 (1.2%)	2 (1.4%)	1 (0.8%)	1 (2.0%)	
Mix	117 (35.7%)	70 (47.6%)	25 (19.1%)	22 (44.0%)	
Antibiotic exposure^a^	18 (5.5%)	10 (6.8%)	4 (3.1%)	4 (8.0%)	0.23
Reported diarrhea^a^	7 (2.1%)	3 (2.0%)	4 (3.1%)	0 (0%)	0.60
RV1 1st dose age (days)					
Median [Q1, Q3]	63.0 [61.0, 70.0]	64.0 [61.5, 69.5]	64.0 [61.0, 73.0]	62.0 [61.0, 64.8]	0.007
RV1 2nd dose 2 age (days)					
Median [Q1, Q3]	127 [123, 136]	126 [123, 137]	128 [123, 142]	128 [124, 131]	0.51

The table shows the demographic and clinical characteristics of the infant cohort. Indicated *P*-values were calculated using the following tests and comparisons between countries: one-sided Chi-squared test (Male sex, Delivery Type); two-sided Fisher’s exact test (Maternal secretor status, Hispanic ethnicity, Infant race, Reported diarrhea, Antibiotic exposure, Diet at rotavirus vaccination (RV) 1st dose); two-sided ANOVA (Mother age, Gestational age); Kruskal–Wallis test (Birth weight, RV1 1st dose age, RV1 2nd dose age). An extended version of this is available as Supplementary Data 3.

^a^From birth to RV1 1st dose.

Overall, 153 of 328 (46.6%) infants demonstrated ≥3-fold increased serum rotavirus-specific IgA (RV-IgA) from pre- to post-vaccination. Neither seroconversion rates nor M2 or M6 seropositivity (defined as serum RV-IgA >20 arbitrary units [AU]) significantly differed between countries (Fig. 1B). Pre-vaccine RV-IgA levels were higher in Peruvian compared to U.S. infants (*χ*² = 23.5, *P* < 0.001, *N* = 328; Dunn’s Test) and similar to Panamanian infants (*χ*² = 23.5, *P* = 0.06, *N* = 328; Dunn’s Test). Panamanian infants displayed similar pre-vaccine IgA than U.S. infants (*P* = 0.06, *N* = 328; Dunn’s Test). (Fig. 1C). Post-vaccination, Panamanian and Peruvian infants demonstrated higher serum RV-IgA than U.S. infants (Fig. 1C). RV-IgA fold change values were lower among Peruvian than Panamanian or U.S. infants (Fig. 1C). Despite differences in RV-IgA levels at both time points, IgA fold-changes did not differ between Panama and U.S. (Fig. 1D). While absolute RV-IgA and magnitude of fold change varied between Panama, Peru, and U.S., the percent of infants in each country that responded to the vaccine was similar. Pre-existing serum RV-IgA prior to vaccination was positively correlated with post-vaccination serum RV-IgA (0.39, *P* = 3e-13, *N* = 328; Spearman’s correlation; Supplementary Fig. 3A, B).

RV1 shedding was detected in fewer patients than seroconversion. Of 216 infants with stool collected 1–14 days after the second RV1 dose, 79 (36.6%) had positive detection (Fig. 1B). Differences between countries were not significant. Shedding and seroconversion were not associated (*Z* = 1.677, *P* = 0.09, *N* = 216; Logistic regression + Wald test).

### Diet, delivery, and country drive differences in microbiome structure at all time points

We generated and sequenced shotgun metagenomes from 816 stool samples collected from the 328 infants at month 1 (M1) (median: 9 DOL), M2 collected prior to vaccination, and at M4 contemporaneous with second vaccine dose. Of those, 51 samples and 4 infants were excluded via quality filtering (Supplementary Fig. 4), culminating in a dataset of 765 metagenome samples from 324 infants, including 252 at M1, 288 at M2, and 225 at M4.

**Fig. 2:**
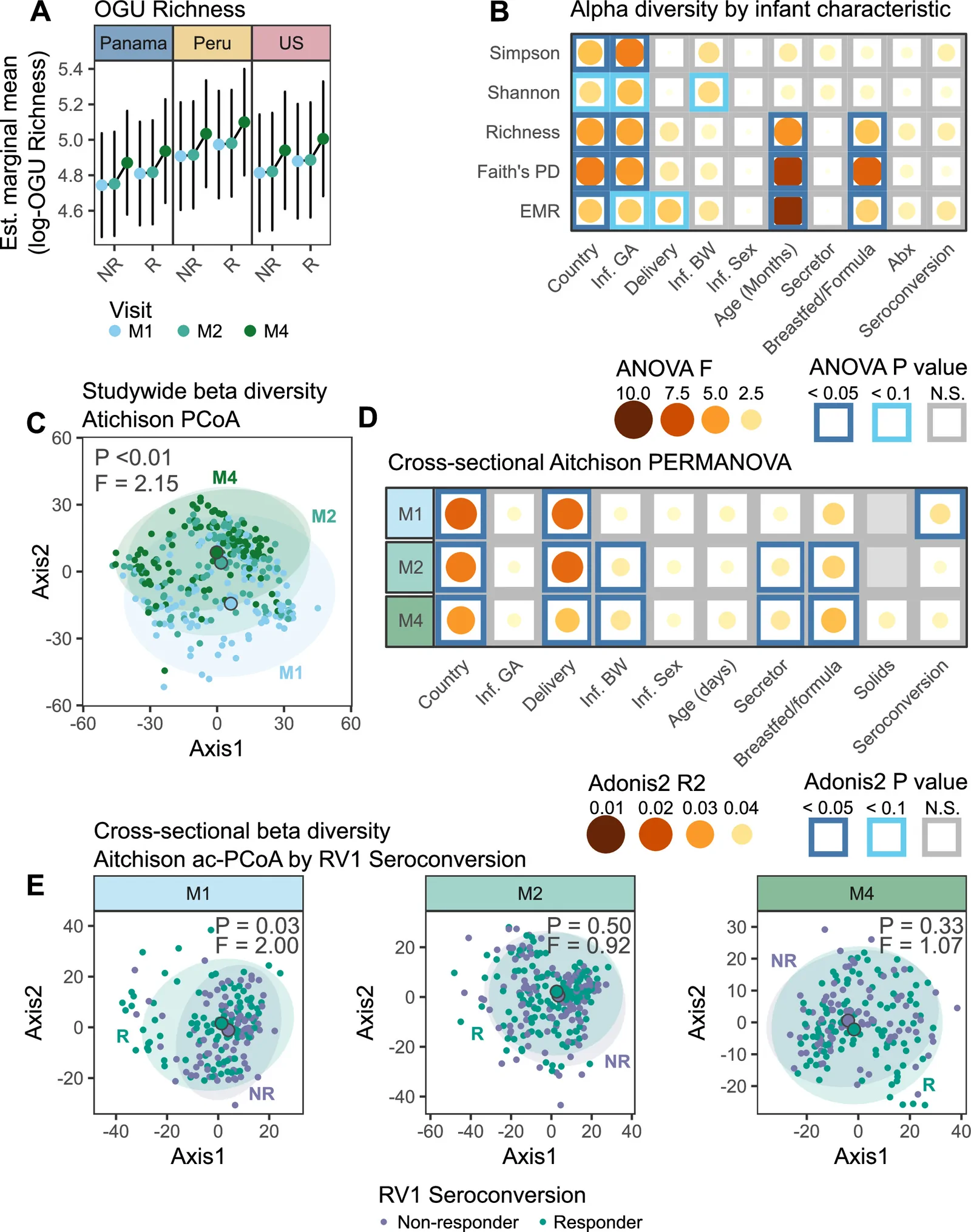
Differences in gut microbiome diversity metrics corresponding to country, visit, seroconversion status, and other variables. **A** Operational Genomic Unit (OGU) richness linear mixed-effects model (LMEM) estimated marginal means for each visit, country, or RV1 responders (R) and non-responders (NR) (*N* = 120 infants). Error bars represent 95% confidence intervals of estimated marginal means. Additional analysis of alpha diversity is available in Supplementary Fig. 6. **B** Results of Partial Sum of Squares Analysis of Variance (partial-SS ANOVA) on alpha diversity LMEMs (*N* = 120). Effect sizes are represented by circles proportional to the F statistic for each model term. Boxes with colored outlines indicate statistical significance (*P* < 0.05, blue) or near-significance (*P* < 0.1, light blue). **C** PCoA ordination showing differences in Aitchison distance in the first (M1) and second (M2) and fourth month (M4) of life. Effect sizes and *P*-values indicated reflect sequential Permutational multivariate analysis of variance (PERMANOVA) of time point. Per-visit centroids are indicated by outlined circles. Ellipses indicate 95% confidence intervals of coordinates. (*N* = 110). **D** Results of marginal PERMANOVA based on Aitchison distances at M1 (*N* = 193), M2 (*N* = 268), and M4 (*N* = 188). Circles are proportional to the *R*^2^ statistic for each term. Colored boxes indicate significance (*p* < 0.05, blue) or near-significance (*p* < 0.1, light blue). None of the infants had received solid foods by M1 or M2. At M4, 39 infants had received solid food. **E** Cross-sectional Aitchison adjusted-for-covariates PCoA (ac-PCoA). Centroids for RV1 seroconversion (responder = R; non-responder = NR) are indicated by outlined circles. Ellipses indicate 95% confidence intervals of coordinates. Effect sizes and *P*-values indicated reflect seroconversion status marginal PERMANOVA tests per (**D**). Additional analysis of beta diversity is available in Supplementary Fig. 7.

As expected, OGU richness increased with infant age and differed between countries (Fig. 2A, B). Beta diversity within the cohort differed with delivery, age, diet, and country (Fig. 2C, D). We evaluated functional diversity based on MetaCyc^17^ pathways in 204 infants. Alpha diversity of microbial functions increased with age with both Shannon (*P* = 0.01–0.024) and Observed (*P* = 0.001–0.007, *N* = 204 infants; PERMANOVA) metrics. Aitchison distance-PERMANOVA revealed country (*R*² = 0.015, *P* = 0.001, *N* = 204; PERMANOVA) and delivery mode (*R*² = 0.015, *P* = 0.001, *N* = 204; PERMANOVA) as primary drivers of differences in functional composition, while age (*R*² = 0.01, *P* = 0.001, *N* = 204; PERMANOVA) accounted for a smaller proportion of variation. These findings demonstrate that clinical features known to associate with microbiome variation in early life likewise contribute to differences within this cohort; delivery mode, age, diet, and geographic context shape both taxonomic and functional maturation of the infant gut microbiome.

### Gut community composition in the first month of life correlates with RV1 seroconversion

Because gut microbiome beta diversity corresponded to infant age, we divided community data into cross-sections by age and included chronological age as a term in our model formulae. Differences in RV1 response correlated to the composition of the gut microbiome at M1 (Fig. 2E). Marginal PERMANOVA^18^ performed at M1 indicated correlation between seroconversion and Aitchison distance (*R*² = 0.011, *P* = 0.030, *N* = 193; PERMANOVA, Fig. 2D, Supplementary Fig. 7). To probe the relationship between beta diversity and post-vaccination IgA response, we applied sequential PERMANOVA including terms for country, delivery, breastfeeding, cross-sectional age, and pre- and post-RV1 IgA, in that order. This test accounts for variation correlated with post-vaccination IgA *after* accounting for variation correlated with other variables. This model indicated differences in post-vaccination RV-IgA were related to community composition at M1 (*R*² = 0.010, *P* = 0.032; *N* = 193). In contrast, no relationship was found between RV1 seroconversion and composition at M2 (*P* = 0.50, *N* = 268) or M4 (*P* = 0.33, *N* = 188). Functional beta diversity based on MetaCyc pathway profiles reflected similar patterns. At M1, Aitchison distance of pathway composition was associated with seroconversion status (*R*² = 0.012, *P* = 0.017, *N* = 193), while no differences were observed at M2 or M4.

**Fig. 3:**
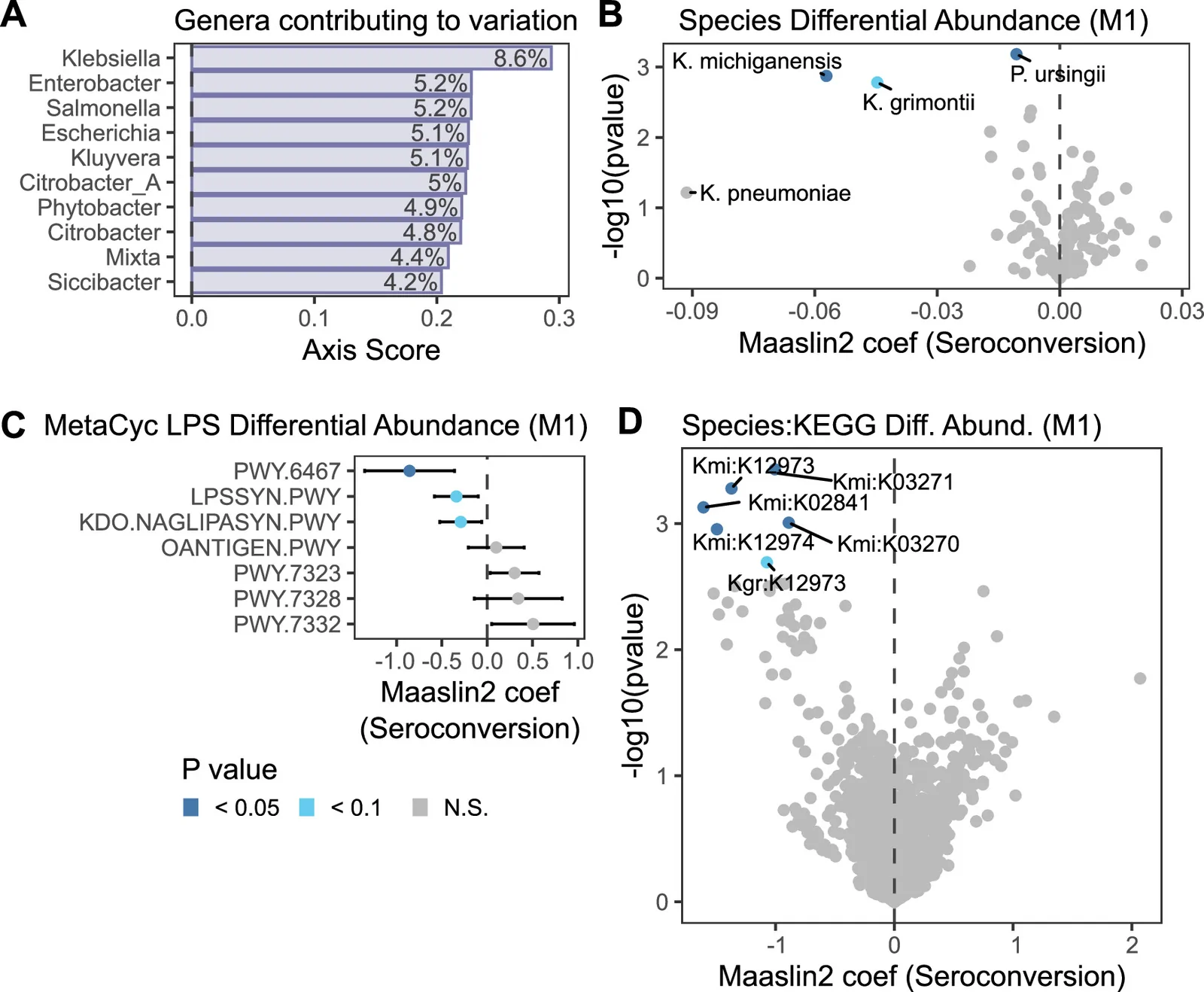
Enrichment of *Klebsiella spp*. and LPS among Rotavirus Vaccination (RV)1 non-responders according to differential abundance analysis and constrained ordination in the first month of life. **A** Top 10 genera contributing to constrained ecological variation correlated to RV1 seroconversion (*N* = 193). Percentages indicate contribution to total variation across distance-based redundancy analysis axis 1 (dbRDA1). Klebsiella was the dominant contributor at 8.6%. **B** Taxonomic differential abundance analysis using MaAsLin2 at M1 (*N* = 252). Maaslin coefficient indicates enrichment among RV1 seroconverters, while a negative coefficient indicates enrichment among non-seroconverters. **C** Differential abundance analysis of M1 MetaCyc pathways involved in LPS biosynthesis (*N* = 204). Pathways shown include the superpathway of LPS biosynthesis (LPSSYN-PWY), (Kdo)2-lipid A biosynthesis (KDO-NAGLIPASYN-PWY), Kdo transfer to lipid IVA III (PWY-6467), O-antigen building blocks biosynthesis (OANTIGEN-PWY; PWY-7323; PWY-7328; PWY-7332). Error bars indicate 95% confidence intervals of MaAsLin2 beta coefficients. **D** Differential abundance using combined M1 species and Kyoto Encyclopedia of Genes and Genomes (KEGG) orthologs (*N* = 204). Species names are abbreviated for clarity (Kmi = *Klebisella michiganensis*; Kgr = *Klebsiella grimontii*).

To identify an ecological gradient correlated to RV1 seroconversion, we performed constrained ordination via distance-based redundancy analysis (dbRDA) of M1 genus-level additive log-ratio^19^-transformed counts, conditioned on country, delivery mode, diet, and cross-sectional age. Constrained ordination recovered a gradient inverse to RV1 seroconversion (*P* = 0.014; ANOVA). The genus *Klebsiella* was the top contributor to this axis (Fig. 3A), explaining 8.6% of the total variation.

### Enrichment of Klebsiella in the first month of life is associated with risk of impeded RV1 response

To identify taxa associated with RV1 response, we performed species-level multivariable differential abundance analysis (DAA)^20–22^. At M1, DAA indicated *Klebsiella michiganensis* and *Phytobacter ursingii* were overrepresented in the gut of RV1 non-responders (Fig. 3B, Supplementary Figs. 8 and 9). No species at M2 or M4 were associated with seroconversion status. These findings were reproducible across multiple methodologies (Supplementary Fig. 8), confirming the robustness of the *Klebsiella* enrichment signature in non-responders. Complementary analyses of MetaCyc^17^ pathways using MaAsLin2^21^ identified 61 pathways enriched in RV1 non-responders, and six enriched in responders (*q* < 0.1) (Supplementary Fig. 8). Pathways enriched in non-responders were dominated by aromatic compound degradation, particularly of toluene and catechol (e.g., PWY-5178, PWY-5179, PWY-5180, PWY-5183, PWY-5417). Additional enriched pathways included ureide biosynthesis (URSIN-PWY), fatty acid and cholesterol biosynthesis (PWY66-341, PWY66-4), trimethylamine degradation (PWY-6962), and amino acid catabolism pathways: arginine (ARGDEG-PWY), ornithine (ORNDEG-PWY), asparagine (ASPASN-PWY), glutamate (GLUDEG-II-PWY), and threonine (THREOCAT-PWY). Other pathways overrepresented in non-responders included phenylalanine biosynthesis (PWY-6628), ubiquinone biosynthesis (UBISYN-PWY), and acetate utilization (ACETATEUTIL-PWY).

Cross-sectional DAA indicated that functional differences between RV1 responders and non-responders were most pronounced in M1. Forty-four pathways were enriched in non-responders, while seven were enriched in responders (Supplementary Fig. 9). At M1, the lipopolysaccharide (LPS) biosynthesis pathway (PWY-6467) was enriched among non-responders. RV1 responders at this same time point demonstrated enriched core energy-generating pathways, including glycolysis with TCA cycle and glyoxylate bypass (GLYCOLYSIS-TCA-GLYOX-BYPASS), as well as phosphatidylglycerol (PWY4FS-7, PWY4FS-8) and colanic acid (COLANSYN-PWY) biosynthesis. At later time points, functional differentiation by seroconversion was reduced. Only six pathways (*q* < 0.1) were differentially abundant at M2, and none at M4.

### Klebsiella-associated LPS define a pro-inflammatory profile in RV1 non-responders

Following the observed enrichment of *Klebsiella* species in RV1 non-responders at M1, accompanied by a distinct metabolic profile characterized by upregulation of LPS biosynthesis, amino acids, and aromatic compounds degradation pathways, we hypothesized that early life *Klebsiella* colonization may contribute to a pro-inflammatory gut mucosal environment through enhanced endotoxin production and pro-inflammatory metabolite generation. We conducted DAA of MetaCyc LPS biosynthesis pathways at M1 (Fig. 3C). All differentially abundant LPS pathways were overrepresented in RV1 non-responders (PWY-6467, LPSSYN-PWY, KDO-NAGLIPASYN-PWY), with no LPS pathways enriched in responders, supporting a group-specific elevation in LPS-producing potential in early life pre-vaccination.

To strengthen the link between taxonomic drivers and functional potential, we performed gene-level DAA of KEGG^23^ orthologs associated with LPS biosynthesis, stratified by species (Fig. 3D). This analysis demonstrated that *Klebsiella* species carrying LPS biosynthetic genes are enriched in RV1 non-responders, while no LPS gene-harboring species are enriched in responders. These findings provide species- and gene-level support for the pathway-level findings and suggest *Klebsiella* overabundance as a driver of the LPS enrichment signature observed in the early-life microbiome of RV1 non-responders.

### RV1 response is associated with gut community type in the first month of life

**Fig. 4:**
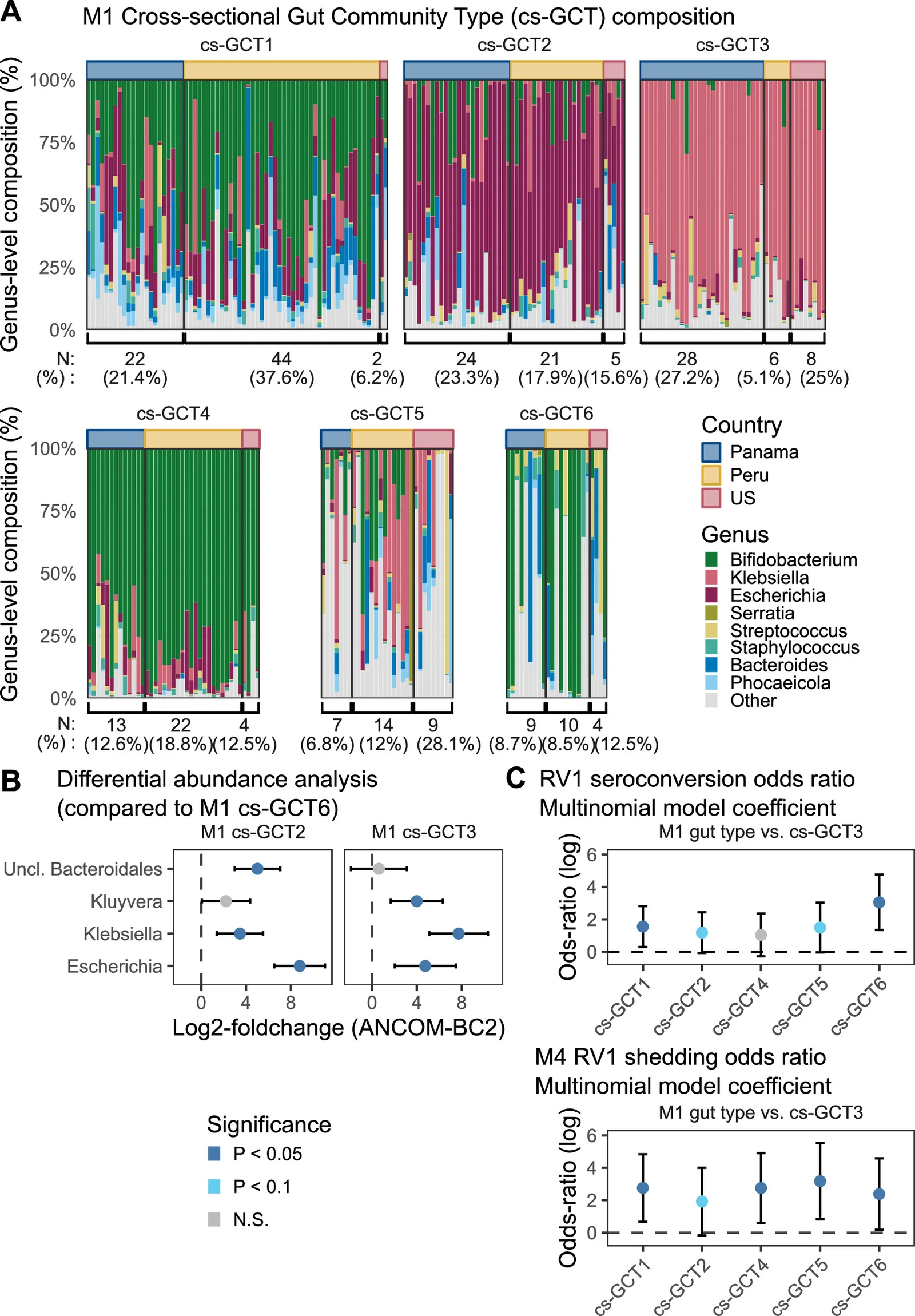
Overview of cross-sectional gut community types (cs-GCTs) in the first month of life (M1) and association with Rotavirus Vaccination 1 (RV1) response differential. **A** Taxonomic bar plots detailing genus-level composition of each M1 gut community type. Additional time points are available in Supplementary Fig. 13. The number and percentage of infants from each country assigned to each cs-GCT are indicated below each facet. **B** Differential abundance analysis (ANCOM-BC2^22^) comparing M1 cs-GCT3 and cs-GCT2 to cs-GCT6 (*N* = 252). Positive differential abundance (log2-foldchange) corresponds to enrichment of a genus in each cs-GCT compared to cs-GCT6. The model used included terms for country, delivery mode, and cumulative diet. Error bars indicate 95% confidence intervals of the fold change as estimated by ANCOM-BC2. **C** Odds ratios (OR) for the association between seroconversion (top) and stool shedding (bottom) based on multinomial logistic regression (*N* = 252) using cs-GCT3 as a pivot value. Significance was assessed using an uncorrected post-hoc Wald test on multinomial model coefficients and standard errors for each RV1 response variable. Points indicate mean log-OR model coefficients. Confidence intervals shown are 2× SEM log-OR.

To describe overall patterns in infant gut community composition, we applied Dirichlet Multinomial Mixture modeling^24^ and generated cross-sectional gut community types (cs-GCTs). These cs-GCTs summarized the complex microbial community composition at each time point (M1, M2, and M4) as discrete categorical community states (Fig. 4A, Supplementary Figs. 11–14). To characterize the taxonomic differences among cs-GCTs, we performed DAA to identify genera that differed between community types (Fig. 4B, Supplementary Fig. 14A, B). Here, the gut type M1 cs-GCT3 was enriched in *Klebsiella*. M1 cs-GCT6, in contrast, was depleted in *Klebsiella* as compared to cs-GCT2 and cs-GCT3 and depleted in *Escherichia* as compared to cs-GCT1, cs-GCT2, cs-GCT3, and cs-GCT4. M1 cs-GCT2 was enriched in *Escherichia* and *Klebsiella* as compared to cs-GCT6 and enriched in *Escherichia* as compared to cs-GCT3. M1 cs-GCT6 was not enriched in *Bifidobacterium* or *Bacteroides* relative to cs-GCT1, cs-GCT5, or cs-GCT4; however, cs-GCT6 was enriched in *Bifidobacterium* relative to both cs-GCT2 and cs-GCT3.

To determine whether gut configurations persisted beyond the M1 time point, sequential PERMANOVA was applied to quantify correlation between M1 cs-GCTs and beta diversity at subsequent time points (Supplementary Fig. 14C). M1 cs-GCTs corresponded to differences in beta diversity at M2 (*R*² = 0.012, *P* = 0.002, *N* = 223; PERMANOVA), indicating temporal autocorrelation in gut composition from M1 to the first RV1 dose. By M4, no relationship was observed (*R*² = 0.012, *P* = 0.92, *N* = 169; PERMANOVA), suggesting that signal from the M1 gut is obscured by diversification of the gut microbiome later in infancy.

Using cs-GCTs as the outcome, a multinomial model was used to quantify the association of clinical variables and RV1 seroconversion with the odds of infants being assigned to particular gut types at each time point. This model was selected to quantify the extent to which RV1 seroconversion associates with each gut type while accounting for other factors related to beta diversity such as country of origin, diet, and breastfeeding. Accounting for these covariates, seroconversion status was associated with overall differences in M1 cs-GCT odds (*G*² = 18.249, *P* = 0.003, *N* = 252; LRT). Model coefficients (log-odds-ratios) revealed that seroconverters displayed lower odds of being associated with *Klebsiella*-dominant cs-GCT3 than cs-GCT1, cs-GCT5, or cs-GCT6 (Fig. 4C). Conversely, seroconverters displayed the highest odds associated with *Klebsiella*- and *Escherichia*-depleted M1 cs-GCT6 relative to cs-GCT3. Likewise, cs-GCT6 included the greatest absolute percentage of seroconverters (17/23; 74.0%) among all gut types. RV1 seroconversion had no impact on the odds ratio observed at M2 (*P* = 0.128; *N* = 288) or M4 (*P* = 0.606; *N* = 188).

Similarly, differences in seropositivity after vaccination were associated with M1 cs-GCT (*χ*² = 17.0, *P* = 0.004, *N* = 252; Pearson’s Chi-squared test) and not any other time point. No differences were observed between M1 cs-GCTs in seropositivity prior to vaccination (*χ*² = 8.72, *P* = 0.12, *N* = 252). Proportionally fewer M1 *Klebsiella*-dominant cs-GCT3 infants (16% [7/42]) were seropositive post-vaccination than other cs-GCTs. Despite lower IgA response, however, M1 cs-GCT3 was also less likely to have detection of RV1 shedding in post-vaccination stool than any other cs-GCTs except for cs-GCT2 (Fig. 4C). Patterns in RV1 shedding and seroconversion were restricted to M1, suggesting persistent association between M1 gut configuration and RV1 response later in life irrespective of gut composition contemporaneous with RV1 administration at M2.

To distinguish differences in IgA response dynamics between gut types, we executed additional multinomial models including (i) a term for pre- and (ii) a term for pre- as well as post-vaccine IgA responses (Supplementary Fig. 12B). Lower RV-IgA fold change and reduced post-vaccination IgA levels (the latter including a term for pre-existing immunity) were associated with the *Klebsiella*-dominant cs-GCT3 as compared to all cs-GCTs except for cs-GCT4. Odds differential between cs-GCT4 and cs-GCT6 may be influenced by differences in prior RV-IgA rather than vaccine response however: M1 *Bifidobacterium*-dominant cs-GCT4 displayed the highest proportion of pre-vaccination (M2) RV1 seropositivity, 21% (8/39) among all M1 gut types (Supplementary Fig. 12A) and likewise was associated with elevated pre-vaccination RV-IgA as compared to cs-GCT6 (Supplementary Fig. 12B). To contrast, low-response cs-GCT3 and cs-GCT2 were not associated differences in pre-vaccination RV-IgA compared to high-response cs-GCT6, suggesting that diminished seroconversion odds associated with these gut types is due to decreased relative post-vaccine IgA independent of confoundment by pre-existing humoral immunity.

### Gut inflammation is correlated with RV1 response

Overgrowth of facultative anaerobes such as *Klebsiella* and *Escherichia* has been implicated as an indicator of an inflamed gut^25,26^. Thus, we hypothesized that a pro-inflammatory gut in M1 may contribute to poorer vaccine responses. We measured fecal calprotectin, a clinical biomarker for gut inflammation. Calprotectin concentration did not differ between countries at M1 (*χ*² = 4.07, *P* = 0.26, *N* = 253; Kruskal–Wallis test); however, by M2, calprotectin was elevated in the stool of Peruvian infants compared to those in Panama and the U.S. (*χ*² = 29.9148; *P* = 0.002 and *P* = 0.004, respectively, *N* = 277; Dunn’s test + BH). At both M1 and M2, neither diet nor delivery mode were significantly related to calprotectin concentration when considering differences between countries.

**Fig. 5:**
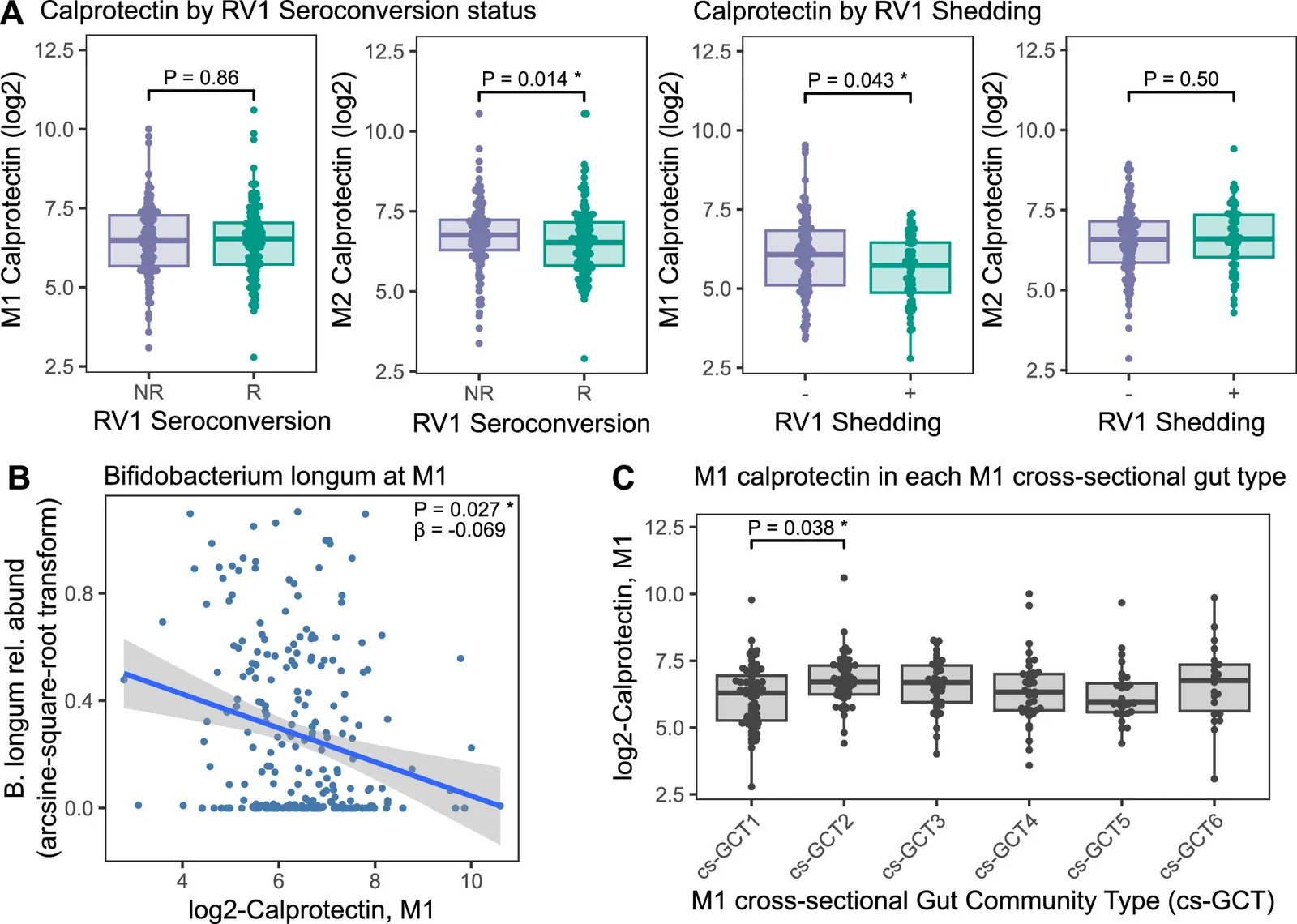
Analysis of fecal calprotectin at M1 and M2. **A** M1 and M2 calprotectin levels compared across seroconversion response (R) or non-response (NR) status (left) and Rotavirus Vaccination 1 (RV1) shedding positive (+) or negative (−) response at M4 (right). Wilcoxon rank-sum tests were used for comparison (*N* = 250). Boxes represent the median and interquartile range (IQR). Whiskers extend to maxima and minima within 1.5x IQR. Outliers are not highlighted. **B** Arcsine square-root -transformed *Bifidobacterium longum* relative abundance at M1 compared to calprotectin at the same time point. Analysis was performed using MaAsLin2 (*N* = 250) and corrected for multiple comparisons using false discovery rate (FDR). The shaded region of the line represents the 95% confidence interval of the regression coefficient calculated via the function geom_smooth. **C** M1 calprotectin compared to M1 cross-sectional Gut Community Type (cs-GCT). Dunn’s Test was used and corrected for multiple comparisons using the BH method (*N* = 242).

Infants with RV1 shedding detected at M4 displayed lower calprotectin concentration at M1 (*W* = 4242.5, *P* = 0.043, *N* = 176; Fig. 5A) but not at M2, despite a correlation between calprotectin at M1 and M2. DAA revealed that calprotectin at M1 was negatively associated with *Bifidobacterium longum* (*β* = −0.069, *P* = 0.026, *N* = 252, MaAsLin2; Fig. 5B) and *Bifidobacterium saguini* (*β* = −0.006, *P* = 0.065, *N* = 252), but not *Klebsiella* or *Escherichia*. These results suggest that early colonization by *Bifidobacterium*, while not linearly related to IgA response, may prevent inflammatory gut phenotypes from developing at this critical period and augment the efficacy of RV1 later in life.

Calprotectin was elevated at M1 among *Klebsiella*- and *Escherichia-* enriched cs-GCT2 infants as compared to cs-GCT1 (*χ*² = 13.0, *P* = 0.038, *N* = 245; Dunn’s Test + BH, Fig. 5C). However, no association was observed between calprotectin concentration and any other cs-GCT. M1 cs-GCT1 and cs-GCT2 are demographically comparable; neither is overrepresented by a particular country (*P* = 0.34), and in both cases, most (54/54 [100%] and 40/43 [93%] of cs-GCT1 and cs-GCT2, respectively) were vaginally delivered and exclusively breastfed by the first time point. *Klebsiella*-rich non-response configuration cs-GCT3 was overrepresented by cesarean-delivered (*z* = −4.109, *P* = 4e-05, *N* = 201; Logistic regression + Wald test) Panamanian (*z* = −2.450; *P* = 0.014, *N* = 201; Logistic regression + Wald test) infants and thus lacked a direct comparator among Peruvian infants.

### Classification models of RV1 response do not generalize

We developed machine learning models using LightGBM to predict seroconversion based on taxonomic composition of the gut microbiome at each time point. Our models were unable to predict seroconversion; when applied to held-out samples, area under the receiver operating characteristic curve (auROC) and area under the precision-recall curve (auPR) were visually comparable to a naive model, and holdout auROC did not exceed 50% using inputs from any time point (Supplementary Fig. 16).

## Discussion

Our study demonstrates that the composition and functional capacity of the early-life gut microbiome are associated with oral RVV immunogenicity in infants. Drawing from a geographically diverse cohort spanning Panama, Peru, and the U.S., we observed that infants with *Klebsiella*- and *Escherichia*-enriched microbiota in the first month of life exhibited lower RV1 seroconversion rates, post-vaccine rotavirus-specific IgA titers, and vaccine virus shedding. These associations were temporally restricted to the first month of life, underscoring the importance of early colonization in shaping mucosal immune responsiveness.

Recent evidence supports early-life microbiome composition impacting vaccine outcomes^8,10,11,13–16^. In a prospective Australian cohort, neonatal antibiotic exposure was associated with decreased antibody responses to multiple vaccines, attributed to reductions in *Bifidobacterium* and other beneficial taxa^10^. While our study centers on oral rather than intramuscular vaccination, the parallels are clear. Rather than a lack of *Bifidobacterium*, we identified overgrowth of *Klebsiella* and *Escherichia* pathobionts alongside enrichment of pro-inflammatory functional pathways, suggesting that early disruption of the gut microbiome from dominance by pathobionts can impair systemic and mucosal vaccine responses.

Prior studies show that RVV is less effective in LMICs, attributed to environmental enteropathy, maternal antibodies, and microbial dysbiosis^27,28^. Our results refine this model by identifying specific microbial configurations rather than global diversity metrics associated with impaired vaccine outcomes. We found that microbial diversity increased as expected with age^11,15,16,29^ but lacked predictive power for RVV outcomes, suggesting that community structure rather than richness is the key determinant of vaccine response. The absence of a global predictive signal in machine learning models further supports the notion that context-specific ecological configurations and host-microbe interactions may drive vaccine outcomes.

Crucially, specific taxonomic and functional configurations in the first month of life, particularly enrichment in *Klebsiella michiganensis*, *Klebsiella grimontii*, and related LPS biosynthetic pathways, were the most consistent correlates of vaccine non-response. Notably, differential abundance of *Klebsiella* spp. was accompanied by overrepresentation of metabolic pathways involved in LPS biosynthesis, aromatic compound degradation, and amino acid catabolism, functional profiles that likely promote a pro-inflammatory mucosal environment. These microbial features are consistent with findings from vaccine efficacy studies in South Asia and sub-Saharan Africa. Harris et al.^13^ showed that oral RVV failure in Indian infants was associated with gut microbiomes enriched in Enterobacteriaceae, including *Klebsiella*, and depleted in *Bifidobacterium*. Similarly, Praharaj et al.^30^ and Becker-Dreps et al.^31^ found that non-polio enterovirus colonization and increased gut inflammation were associated with reduced poliovirus and RVV responses. Together, these studies and our results converge on a theme whereby inflammatory gut environments, often defined by overgrowth of facultative anaerobes like *Klebsiella* and *Escherichia*, reduce the efficacy of oral vaccines in early infancy.

To assess whether these microbial signatures corresponded with an inflammatory gut environment, we evaluated fecal calprotectin concentrations as a clinical biomarker of mucosal inflammation. We found that infants with lower M1 calprotectin levels were more likely to have detectable RV1 virus after vaccination, linking lower early-life inflammation with enhanced mucosal replication of the vaccine virus. At M2, calprotectin was associated with pre-vaccine RV-IgA more than with vaccine response itself, suggesting that prior exposure to circulating rotavirus may drive some postnatal inflammation. These observations reflect the complex interplay between exposure history, microbial composition, and mucosal inflammatory tone. Linear modeling revealed no relationship between post-vaccine IgA and calprotectin when controlling for pre-vaccine titers, indicating that inflammation may interfere with vaccine take^11^ rather than the magnitude of IgA induction.

De Koff et al.^12^ demonstrated that neonatal immune states shaped by early environmental exposures, including microbial cues, can determine systemic vaccine responses over the first year of life. Our work deepens this framework by linking gut microbiome ecotypes and functional gene content with RV1-specific immune outcomes. Notably, community state typing revealed that infants having *Klebsiella*- and *Escherichia*-depleted configurations (cs-GCT6) demonstrated higher vaccine responsiveness. Intriguingly, high-response types were not characterized by enrichment of “probiotic” genera like *Bifidobacterium*, suggesting that protection against vaccine hypo-responsiveness may result from exclusion of pro-inflammatory taxa rather than enrichment of beneficial ones. This interpretation is consistent with the finding that reduced *Proteobacteria* abundance at birth correlated with improved vaccine shedding following neonatal RV3-BB administration in Indonesia and Malawi by Wagner et al.^11^. Lazarus et al.^32^ also reported that probiotic supplementation did not improve RV1 immunogenicity among infants in India. Additionally, *Klebsiella*- and *Escherichia*-enriched community types (e.g., cs-GCT2) displayed higher calprotectin levels compared to demographically similar, pathobiont-depleted configurations (e.g., cs-GCT1), further implicating early inflammatory gut states in oral vaccine non-responsiveness. *Bifidobacterium longum* and *B. saguini* were inversely correlated with calprotectin in the first month of life, even though they were not predictive of seroconversion. These taxa may help buffer the gut against inflammation during this critical developmental window, promoting a mucosal environment conducive to vaccine uptake.

Our findings also provide additional context for the paradoxical relationship between shedding and seroconversion in LMICs^33,34^. Lee et al.^33^ proposed absence of second-dose RV1 shedding may reflect protective immunity from the first dose. Here, we found the opposite: *Klebsiella*- and *Escherichia*-enriched gut types were associated with both lower RV-IgA response *and* inhibited RV1 replication. The relationship between RV1 replication and calprotectin observed here suggests an inflammatory gut may reflect neutrophil-driven clearance of the vaccine virus in an inflamed gut without concurrent generation of effective adaptive immune memory function. Previous research links gut microbiome and fecal calprotectin to increased neutrophil activation in inflammatory bowel disease and Graft-versus-host disorders^35–38^. Enhanced neutrophil activity may suppress immune memory^39^ through competition with dendritic cells for antigen^40^ or by inhibition of IgA production^41^. Although neutrophils clear infections^42^ and may limit RV1 replication, this occurs at the expense of long-term humoral immunity. The relationship between neutrophils and humoral immunity to rotavirus requires further research.

Mechanistically, our findings reinforce the role of LPS-rich, facultative anaerobes like *Klebsiella* in promoting inflammatory gut states that disrupt antigen presentation and immune priming. Animal models further support this framework. Lynn et al.^43^ demonstrated that neonatal microbiome disruption impaired both mucosal and systemic vaccine responses in mice, which could be rescued via microbial restoration. Together, these lines of evidence suggest that pathobiont-associated inflammation at the time of vaccine dosing reduces the likelihood of oral vaccine success.

Our findings underscore the need for microbiome-targeted strategies to enhance vaccine responsiveness. Preclinical studies suggest that maternal or neonatal probiotic supplementation^44^ could improve oral vaccine efficacy. This is further supported by Harris et al.^45^, who proposed modulation of the gut microbiome as a potential avenue for improvement of oral RVV efficacy in LMICs. More strategies, including synbiotic combinations, selective prebiotics to support *Bifidobacterium*, or targeted methods such as phage-based depletion of *Klebsiella*, may offer greater specificity and efficacy. Critically, the timing of these interventions should align with the neonatal window, during which the microbiome and immune system are most dynamically interacting.

Limitations to our study are imposed by complications in the estimation of RV1 immunity and of infant gut microbiome data. Seroconversion measured in blood specimens represents *systemic* IgA response rather than localized gut response where protection is needed. However, at this time, rotavirus IgA seroconversion is the measurement used in clinical trials despite its limitations. The degree of seroconversion may be confounded by pre-existing immunity to rotavirus found in this cohort and others. Transplacentally-acquired maternal IgG can decrease RVV immunogenicity^46^; we did not assess maternal RV-specific antibody levels. Human milk may contain immunologic and compositional factors that impact vaccine response. This study was observational; our framework is unable to assess a causal relationship between RV1 response and the infant gut microbiome. The ecological pattern that we identify correlates with differences in RV1 response, but only after considering the contribution of covariates to gut composition. We structured our ecological analysis to account for confoundment; however, we were unable to identify a published machine-learning workflow to satisfy this constraint. A modified workflow may enable prediction of RV1 seroconversion non-response based solely on gut microbiome data. Nevertheless, the lack of a globally generalizable model remains a key limitation of our findings.

Overall, we identify a microbial-metabolic-inflammatory axis centered on *Klebsiella* colonization and early-life gut inflammation that may impair RV1 efficacy. These findings have important implications for precision vaccine approaches and underscore the need to consider microbiome composition and inflammatory tone when developing strategies to improve oral vaccine performance globally.

## Methods

### Study design and participants

Healthy mother and infant (born ≥35 weeks’ gestation) pairs were enrolled prior to the infant’s first RVV dose at Children’s Hospital Los Angeles (CHLA; Los Angeles, USA), Kaiser Permanente (Los Angeles, USA), Universidad Peruana Cayetano Heredia (UPCH; Lima, Peru), and Hospital del Niño Doctor Jose Renan Esquivel (Panama City, Panama), using a convenience recruitment strategy. Infants enrolled in the study were vaccinated with the oral live, attenuated human monovalent RVV (RV1, Rotarix™, GlaxoSmithKline) at 2 months and 4 months of age per the routine immunization schedule in each country.

Demographic characteristics and comorbidities for mother and infant were collected by survey at enrollment. Sex, race, and ethnicity were documented as reported by parents. Medical and detailed feeding history (breastfeeding frequency, duration, formula intake, introduction to solid food) were collected at enrollment and every follow-up visit at 1–2 weeks (M1), 2 (M2), 4 (M4), and 6 months (M6) of age.

### Ethics statement

This research complies with all relevant ethical regulations. Written, informed consent was obtained from all participants or their parents / legal guardians. Institutional review boards at CHLA, Kaiser, UPCH, and Hospital del Niño each approved the study at their respective sites.

### Statistics and reproducibility

RVV vaccine seroconversion rates by country were used to determine sample size based on the following parameters: 90% RVV seroconversion in high-income countries, 74% in Panama, and 66% in Peru, power = 0.8, *α* = 0.05, a sample size of 255 infants was included in the study. To account for longitudinal subject retention of 80% at 6 months, we aimed to enroll until reaching a total of 330 infants who completed the pre-vaccination visit. Our final dataset included 324 infants from whom we collected pre- and post-vaccination blood samples with stool for metagenomic analysis that passed quality filtering. Infants were excluded if they did not complete study visits or obtain RV1 per the recommended vaccination schedule: a dose at each of 2 and 4 months. There was no blinding. This study was observational and thus no randomization was performed.

### Specimen collection and processing

Infant blood samples were collected pre- and post-vaccination at 2 and 6 months, respectively. Samples were transported to the laboratory within 2–4 h after collection for processing. Plasma and serum were isolated by centrifugation, aliquoted, and stored at −80 °C at each center.

Human milk samples were self-collected just prior to or during the study visit into sterile containers using electric or manual pumps. Cells and fat from human milk samples were removed by centrifuging twice at 1000 × *g* for 10 min. The separated supernatant was then stored at −80 °C.

Stool samples were collected at 1–2 weeks (M1), 2 (M2), and 4 (M4) months of age and stored at −80 °C. Blood, human milk, and stool samples from all sites were shipped to UCSD on dry ice for further analysis.

### Serum IgA and RV1 seroconversion status

Rotavirus-specific blood IgA (RV-IgA) in serum or plasma was measured using an enzyme-linked immunosorbent assay (ELISA), as previously described^7^. Plates were incubated with 1:20 diluted serum samples in diluent buffer and a 1:10 diluted positive standard control, serially diluted 1:3. Plates were then washed and stained with goat anti-human IgA secondary antibody (Southern Biotech, Birmingham, AL) diluted 1:5000, and then reactions were developed with 3,3′,5,5′-tetramethylbenzidine (TMB). The plate reaction was quenched with 2 M sulfuric acid, and optical density (OD) values were measured at 450 nm immediately after the reaction was quenched. Seroconversion was defined as a ≥3-fold-change in post-vaccination infant serum RV-IgA compared with pre-vaccination serum RV-IgA. A seropositivity value of ≥20 AU/mL was determined for serum RV-IgA based on the median OD450 values of all pre-vaccination samples plus the 75th percentile.

### Maternal secretor status

Maternal secretor status was determined by detection using high-performance liquid chromatography (HPLC) of α-1,2 fucosylated human milk oligosaccharides (HMOs), as described previously^47,48^. Secretor status was defined by presence or near absence of 2′FL or LNFP-I, as the maternal secretor locus produces higher concentrations of these α-1,2 fucosylated HMOs.

### RV1 shedding polymerase chain reaction

Stool aliquots weighing 150 µg were resuspended in PBS and then homogenized. RNA was extracted from the homogenized stool solutions using an eMAG nucleic acid extraction system (Biomeriéux, Marcy-l’Étoile, France). Custom RV1 vaccine-strain-targeted oligonucleotide sequences (Integrated DNA Technologies, Coralville, IA) were used as forward and reverse primers in detecting amplified vaccine virus in stool. A panel of master mix (Thermo Fisher Scientific, Waltham, MA) in addition to forward and reverse primers(F: 5′-GAACTTCCTTGAATATAAGATCACACTGA-3′, R: 5′-TTGAAGACGTAAATGCATACCAATTC-3′), and probes (5′-/56-FAM/TCCAATAGATTGAAGT/iAP-dC/AGTAA/ipdC/GTTTCCA/3BHQ1/-3′) were utilized to perform RT-qPCR on M4 stool samples. Samples with a cycle threshold of <40 were considered positive for RV1 shedding.

### DNA extraction and metagenomic next-generation sequencing analysis protocol

Total nucleic acid was extracted from fecal samples using the MagMAX Microbiome Ultra Kit (Thermo Fisher Scientific, Cat#A42357) on KingFisher Flex robots following published protocols. Blank controls and ZymoBIOMICS standards (Zymo Research) were included in each extraction batch. Purified DNA was quantified via PicoGreen assay (Thermo Fisher Scientific), and shotgun metagenomic libraries were prepared using KAPA HyperPlus kits (Roche Diagnostics) on EpMotion automated systems (Eppendorf). Paired-end sequencing (2 × 150 bp) was performed on Illumina NovaSeq X Plus at UC San Diego’s Institute for Genomic Medicine.

Sequences were quality-filtered using fastp (v0.22)^49^ and depleted of human reads by sequential alignment to the GRCh38 reference genome and PhiX control, and the T2T-Y^50^ reference genome using minimap2 (v2.22)^51^. Non-human sequence reads were extracted, converted to FASTQ format using SAMtools (v1.15.1)^52^, and aligned to the Web of Life reference version 2^53^ with Bowtie2 version 2.4.2^54^. Alignment profiles were translated to feature tables of Operational Genomic Units (OGUs) and functional profiling using MetaCyc^17,55^ and KEGG^23,56^ annotations, and were performed using Woltka version 0.1.5^53^. OGUs with <1% coverage were removed, yielding a dataset of 9,556,896,551 reads across 928 samples and 1698 OGUs, with a median sequencing depth of 7,016,230 (IQR: 8,469,467) reads per sample. Samples with fewer than 500,000 reads were subsequently removed, which also excluded all negative controls and blanks, resulting in 835 samples. Positive controls were further removed, yielding a final dataset of 792 fecal samples comprising 9,016,814,196 reads and 1623 OGUs, with a median sequencing depth of 7,520,388 (IQR: 8,216,599) reads per sample.

The filtered biom files were imported into R version 4.4.0^57^ using the biomformat package version 1.32.0^58^. Reagent contaminant OGUs were identified using phyloseq version 1.48.0^59^ and decontam version 1.24.0^60^ and were subsequently removed. The Web of Life reference phylogeny was imported into the R environment using ape version 5.8^61^ and rooted at the phylogenetic midpoint using phytools version 2.1-1^62^. Reference taxonomy reflecting Genome Taxonomy Database 202^63^ designations for each OGU was obtained from the Web of Life release 2. The resulting decontaminated OGU matrix, phylogenetic tree, and taxonomy were imported into phyloseq and subsequently converted into a tree-summarized-experiment (tse) object using mia version 1.12.0^64^.

### Microbiome diversity analyses

The decontaminated OGU count matrix was rarefied using fast subsampling-with-replacement offered by the R package dqrng version 0.4.1^65^ to a per-sample depth of 500,000 counts, resulting in 1575 OGUs. Samples with fewer than 500,000 counts were removed from all downstream analysis. The unrarefied count matrix was also agglomerated at the species and genus levels, and at each level was filtered such that only features in greater than 1% abundance in any sample and prevalent across greater than 5% of all samples were retained. Centered-log-ratio (CLR) transformation was performed on Genus and Species -level matrices using the *clr* function from the R package compositions version 2.0-8^66^, with a pseudocount of 1. Shannon’s diversity and Simpson’s evenness were calculated from the rarefied count matrix using the *diversity* function from vegan version 2.6-6^18^. OGU richness and Faith’s PD were calculated using the *pd* function from picante version 1.8.2^67^. Unrarefied and filtered, as well as rarefied, OGU, species, and genus matrices and their CLR-transformed counterparts were used as inputs for downstream analyses.

To assess the impact of clinical variation on alpha diversity among the cohort across time, linear mixed models were constructed with each of the alpha diversity metrics as the dependent variable using the R package lme4 version 1.1-35.3^68^. Individuals were excluded from this analysis if they were missing samples at any time point. Country-of-origin, delivery mode, age in days, seroconversion status, cumulative feeding habits, recent antibiotic status, infant birthweight, and infant gestational age were provided to the model as fixed-effect terms, while the coded identifier for the individual infant was provided as a random-intercept term. To ensure that no terms were collinear, GVIFs were calculated using the *vif* function from the R package car version 3.1-2^69^. To ensure normality of the dependent variables, transformations were applied within the model formula: OGU richness and Faith’s PD were log-transformed, Simpson’s evenness was logit-transformed, and Shannon diversity was not transformed. Normality of residuals was evaluated visually using the *stat_qq* and *stat_qq_line* functions offered by the R package ggplot2 version 3.5.1^70^. Statistical significance of terms was assessed using mixed-model ANOVA with Kenward-Roger approximation via the mixed function from the R package *afex* version 1.3.1^71^.

MetaCyc functional pathway abundance table from shotgun metagenomic sequencing was imported and processed using the phyloseq package in R. Rare pathways appearing in fewer than 10% of samples with a minimum abundance threshold of >1 count were excluded, retaining 319 pathways. To equalize sampling effort across samples and maximize sample retention prior to alpha diversity estimation, data were rarefied to 7578 pathway-mapped reads per sample, resulting in 793 samples for analysis. Observed and Shannon alpha diversity indices were calculated from rarefied data using the using the phyloseq package. To evaluate robustness to normalization strategy, Shannon diversity was additionally computed using non-rarefied pathway abundances normalized to relative abundance, which yielded highly consistent results (Spearman *ρ* = 0.983, *p* < 2.2 × 10⁻¹⁶). Linear mixed-effects models were fit using lmerTest to examine associations between log-transformed Observed and Shannon diversity and fixed effects including seroconversion status, clinical and demographic variables.

Taxonomic beta-diversity analyses were performed within each time point using functions from the R package vegan version 2.6-6^18^. Bray-Curtis dissimilarity was calculated using the *vegdist* function from vegan with the rarefied species-count matrix. Weighted and unweighted UniFrac distances were calculated using the *unifrac* function from the R package rbiom 1.0.3^72^, provided with inputs of the rarefied OGU count matrix and the Web of Life OGU reference tree. Aitchison distance was calculated by passing CLR-transformed species counts to Euclidean distance calculation via the *vegdist* function. To assess the significance of relationships between beta diversity and clinical or demographic variables, a marginal test was performed for each distance/dissimilarity metric at each time point via *adonis2* from vegan. Cross-sectional distance or dissimilarity matrices were provided to the function as dependent variables. Country, mode of delivery, cumulative dietary habits (formula/breastfeeding/both), introduction of solid food, infant birth weight, infant gestational age, infant gender, infant seroconversion status, cross-sectional age, and mother’s secretor status were provided to *adonis2* as dependent variables. Where a marginal relationship was found between seroconversion and beta diversity, an additional sequential *adonis2* call was performed to distinguish the relationship between beta diversity and pre-existing immunity to rotavirus from differences in post-vaccine IgA titer. Terms included, in order: delivery, cumulative diet, cross-sectional age, country, and pre- and post-vaccine IgA. Missing data were handled by the “na.omit” function. Functions *pcoa* from R package ape version 5.8^61^ and *acPCoA* from R package acPCoA version 1.0^73^ were used to render PCoA and acPCoA ordinations, respectively.

For the analysis of functional beta diversity, Aitchison distance was calculated by applying CLR transformation to pathway abundance data (after adding a pseudocount of 1) followed by Euclidean distance calculation using the Microbiome package. Permutational multivariate analysis of variance (PERMANOVA) was performed using adonis2 with 999 permutations to test associations between microbial community composition and metadata variables. To account for the repeated measures design, permutations were performed within subject blocks. Cross-sectional analysis within each visit was done using the same statistical approaches but without the repeated measures constraints.

### Differential abundance analyses (DAA)

Taxonomic DAA was carried out using MaAsLin2 version 1.18.0^21^, ANCOMBC version 2.6.0^22,74^, and LinDA from R package MicrobiomeStat version 1.2^20^. The count matrix was prepared by first agglomerating the tse object at the species rank, and then by filtering species to retain those present in at least 1% abundance among at least 5% of samples. A statistical model comparable between the three methods was used, including fixed effect terms for cross-sectional time point, country, feeding modality (formula/breastmilk/both), mode of delivery, per-sample read depth, and seroconversion status. For longitudinal DAA, a random effect term was included representing the individual enrollee. For MaAsLin2, the arcsine square-root (AST) was selected, and features were scaled using total sum scaling (TSS). Cross-sectional DAA was performed separately using three slices of the original tse object, each representing data from a single time point. Only MaAsLin2 was applied for cross-sectional DAA, and random effects were omitted from the model. For cross-sectional DAA, agglomeration and filtering were performed according to the same steps described above, but applied separately to each cross-section. Multiple testing correction was applied using the Benjamini–Hochberg method.

Pathway-level and KO gene differential abundance testing was performed using MaAsLin2 with linear mixed-effects models to account for repeated measures. Data were normalized using TSS followed by log transformation. The model included similar covariates used in taxonomic DAA as fixed effects, with enrollment ID as a random effect. Cross-sectional DAA were also performed separately at each time point using the same approach but without random effects. Multiple testing correction was applied using the Benjamini–Hochberg method with a significance threshold of *q* < 0.10.

### Clustering of gut types and multinomial models

Infant gut microbiome samples were clustered into cross-sectional gut community types (cs-GCT) based on a series of Dirichlet Multinomial Mixed models^24^. The unrarefied, filtered, Genus-level matrix was divided into cross-sections based on time point, and each cross-section was passed to the *dmn* function from the R package DirichletMultinomial version 1.46.0^24^. The optimal number of clusters (DMMs) at each time point was determined based on the minimum Laplace score returned by the *laplace* function from the same package. Association of demographic and clinical variables with each DMM was quantified using a multinomial logistic regression executed via the *multinom* function from the R package nnet version 7.3-19^75^. Variables included were selected based on statistical significance in alpha- or beta -diversity analyses and included cross-sectional age. RV1 vaccine responses (seroconversion, pre- and post- vaccine IgA titer, and shedding) were included as terms in the model to determine marginal association with each DMM. For later time points, the state at the previous time point was included as a term to distinguish novel associations. Statistical significance of each term was determined using marginal ANOVA as implemented in the R package car version 3.1-2^69^. Magnitude (as log-odds-ratio) and significance of association between RV1 response differential and specific DMM-contrasts were quantified by fixing the pivot value and then using a Wald test on resulting model coefficients.

### Fecal calprotectin quantification

Stool calprotectin levels were measured using the IDK® Calprotectin ELISA kit (Immundiagnostik AG, Germany) according to the manufacturer’s protocol. Briefly, approximately 15 mg of each stool specimen was weighed and transferred into a tube. Extraction buffer was added as instructed by the manufacturer, and the sample was thoroughly homogenized using a vortex mixer to ensure complete suspension.

The homogenized samples were centrifuged to pellet any particulate matter. The supernatant was carefully collected and diluted 1:2500 in sample dilution buffer for the ELISA. Diluted samples were added to microplate wells pre-coated with anti-calprotectin antibody. The plate was incubated, washed, and then incubated with the provided enzyme conjugate. After additional washing steps, substrate solution was added, and the plate was incubated in the dark for color development. The reaction was stopped using stop solution, and absorbance was measured at 450 nm with a microplate reader. Calprotectin concentrations were determined from a standard curve generated with kit standards.

### Machine learning model development

The tidymodels^76^ machine learning framework was used to develop LightGBM^77^ classifiers for RV1 seroconversion, RV1 stool shedding, and delivery mode based on cross-sectional species and genus matrices combined with alpha diversity data. Unrarefied, unfiltered OGU matrices were divided into cross-sections, and then each matrix was then further divided into training (70%) and holdout (30%) fractions. Each training fraction was filtered to retain taxa present in at least 1% abundance among at least 5% of training samples. The same taxa were retained in the holdout fraction. The training fractions were then used to train binary classifier models using the machine learning framework offered by the R package tidymodels version 1.2.0^76^. For each time point, a classifier targeting RV1 seroconversion (or delivery) was fit using the LightGBM wrapper offered by the R package bonsai version 0.3.0^78^. Models were subject to 5-fold cross-validation, and hyperparameters were tuned based using a 500-row Latin hypercube grid. Final model selection was based on the Brier class score to account for class imbalances. Receiver operating characteristic (ROC) and precision-recall (PR) curves were calculated using the R package pROC version 1.18.5^79^. Feature importance scores for classifier decisions were calculated based on the full dataset using the R package kernelshap version 0.6.0^80^.

### Reporting summary

Further information on research design is available in the Nature Portfolio Reporting Summary linked to this article.

## Supplementary information

Supplementary Information

Supplementary Data 1

Supplementary Data 2

Supplementary Data 3

Supplementary Data 4

Supplementary Data 5

Reporting Summary

Transparent Peer Review file

## Source data

Source data

## Data Availability

Raw shotgun metagenomic data generated here are available on the NCBI Sequence Read Archive under the BioProject accession number PRJNA1404625. Source data for each figure are provided with this paper. Sample metadata, including demographic and RV1 response variables, are provided in Supplementary Data 1. Source data are provided with this paper.

## Code availability

Code used for this analysis is available via the GitHub repository PIALabUCSD/rv1-metagenomics-2025 [https://github.com/PiaLabUCSD/rv1-metagenomics-2025].
