# Supplementary material for: Klebsiella-dominant gut communities are associated with impeded oral rotavirus vaccine response in infants
Source: Nat Commun. 2026 Oct 15;17:10783. doi: 10.1038/s41467-026-78148-x (PMC13672339; doi:10.1038/s41467-026-78148-x)
Supplement: Supplementary file 1 [file 41467_2026_78148_MOESM1_ESM.pdf]

Klebsiella-dominant gut communities are associated with impeded oral rotavirus vaccine response in infants

Supplementary Information

Cale O. Seymour[1,*], Gertrude Ecklu-Mensah[1,2*], Yesun Lee [1], Alexander R. Nicols[1], Wesley A. Cheng[1], Jaycee Jumarang[1], Carolyn Jennifer Marentes Ruiz[1], Jocelyn Navarro[1], Rafaella Navarro[3], Yamile Rodriguez-Angeles[3], David Durand[3], Sarah Allard[1,2], Zackary W. Taylor[4], Ruth G. De León[5], Theresa J. Ochoa[3], Jack A. Gilbert [1,2], Pia S. Pannaraj [1,6]

[1] Department of Pediatrics, University of California, San Diego, La Jolla, CA, United States

[2] Scripps Institution of Oceanography, University of California San Diego, La Jolla, CA, United States

[3] Instituto de Medicina Tropical Alexander von Humboldt and Laboratorios de Investigación y Desarrollo, Universidad Peruana Cayetano Heredia, Lima, Peru.

[4] Department of Pediatrics, Kaiser Permanente, Los Angeles, CA, United States.

[5] Department of Sexual and Reproductive Health, Gorgas Memorial Institute for Health Studies, Panama City, Panama.

[6] Division of Infectious Diseases, Rady Children's Hospital, La Jolla, CA, United States.

[*] Equal contribution

Contents

Contents 2
Supplementary Datafile Descriptions 3
Supplementary text 4
Supplementary methods 4
Model formula 4
Alpha diversity model formula 4
Alpha diversity linear mixed-effects model formula 4
Adonis2 model formula 4
Sequential adonis2 with restricted perms 4
Cross-sectional marginal adonis2 4
Cross-sectional sequential adonis2 4
NB cs-GCT DMM adonis2 (sequential) 4
dbRDA model formula 4
Differential abundance analysis model formula 5
Longitudinal differential abundance analysis formula 5
Cross-sectional differential abundance analysis formula 5
Multinomial logistic regression formula 5
Supplementary Figures 6
Supplementary References 22

Supplementary Datafile Descriptions

Supplementary Data 1. Metagenomes sample metadata. Sample metadata for the metagenomic data described in this study. Samples were excluded from analysis if: (a) they failed to yield 500,000 sequence reads or (b) if Seroconversion status was not defined for the patient.

Supplementary Data 2. OGU table. Unfiltered and unrarefied OGU table generated via metagenomic analysis. Domain-through-OGU taxonomy is specified in the first eight columns. Additional columns indicate the OGU count associated with each sample as specified in Supplementary Data 1.

Supplementary Data 3. Extended demographics table. Indicated P-values were calculated using the following tests and comparing between countries: one-sided Chi-squared test (Male sex, Delivery Type); two-sided Fisher's exact test (Maternal secretor status, Hispanic ethnicity, Infant race, Reported diarrhea, Antibiotic exposure, Diet at rotavirus vaccination (RV) 1st dose), two-sided ANOVA (Mother age, Gestational age), Kruskal-Wallis test (Birth weight, RV1 1st dose age, RV1 2nd dose age).

Supplementary Data 4. Model terms. Table including a description of model formula terms and data types used in this study.

Supplementary Data 5. STORMs checklist. Completed STORMS checklist for metagenomic survey reporting.

Supplementary text

Supplementary methods

Model formula

Several statistical model formulas were used to account for factors that alter the colonization scheme of the infant gut. These formulas are listed below, with the term of interest indicated in bold. VIFs were calculated using the R package car[2] version 3.1.2; a dummy term was provided as the dependent variable of a linear model via the *lm* function, and the output of this linear regression was then passed to the *vif* function. Model terms were selected based on both VIFs and a reasonable understanding of the factors that impact composition of the infant gut microbiome: Diet, delivery, age (either cross-sectional or based on visit), and country were included in all models, however VIFs for all models including these terms were lower than 3. A description of terms is included as a table in **Supplementary Data 4**.

Alpha diversity model formula

Alpha diversity linear mixed-effects model formula

alpha_div_metric ~ formula_bm + visit + abx_status + country + sex + inf_birth_weight + gest_age + delivery + **Seroconverted** + Secretor + (1|enrollment_id)

Adonis2 model formula

Sequential adonis2 with restricted perms

*distance ~ delivery + formula_bm + **visit** + country + **Seroconverted***

Cross-sectional marginal adonis2

*distance_at_timepoint ~ age + delivery + formula_bm + Secretor + solid_food_introduced + inf_birth_weight + gest_age + country + sex + **Seroconverted***

Cross-sectional sequential adonis2

*distance_at_timepoint ~ formula_bm + delivery + age + country + gest_age + log(pre_rota_sero_iga) + **log(post_rota_sero_iga)***

NB cs-GCT DMM adonis2 (sequential)

*distance_at_timepoint ~ formula_bm + delivery + country + age + gest_age + **DMM***

dbRDA model formula

*distance_at_timepoint ~ Condition(delivery + formula_bm + country) + **Seroconverted***

Differential abundance analysis model formula

Longitudinal differential abundance analysis formula

taxon_abundance ~ *delivery* + visit + country + formula_bm + delivery + gest_age + library_size + **Seroconverted** + (1|enrollment_id)

reference levels: country,Panama; formula_bm,BOTH; visit,NB

Cross-sectional differential abundance analysis formula

taxon_abundance ~ *delivery* + age + country + formula_bm + delivery + gest_age + library_size + **Seroconverted**

reference levels: country,Panama; formula_bm,BOTH

Multinomial logistic regression formula

For timepoints [NB, 2mo, 4mo]: *DMM_at_visit* ~ *DMM_at_previous_visit* + *country* + *delivery* + *formula_bm_at_visit* + *gest_age* + *agedifference_current_and_previous* + ***Seroconverted***

For timepoints [NB, 2mo, 4mo]: *DMM_at_timepoint DMM_at_previous_visit* + *country* + *delivery* + *formula_bm_at_visit* + *gest_age* + *agedifference_current_and_previous* + *log(pre_vaccine_iga)* + ***log(post_vaccine_iga)***

Supplementary Figures

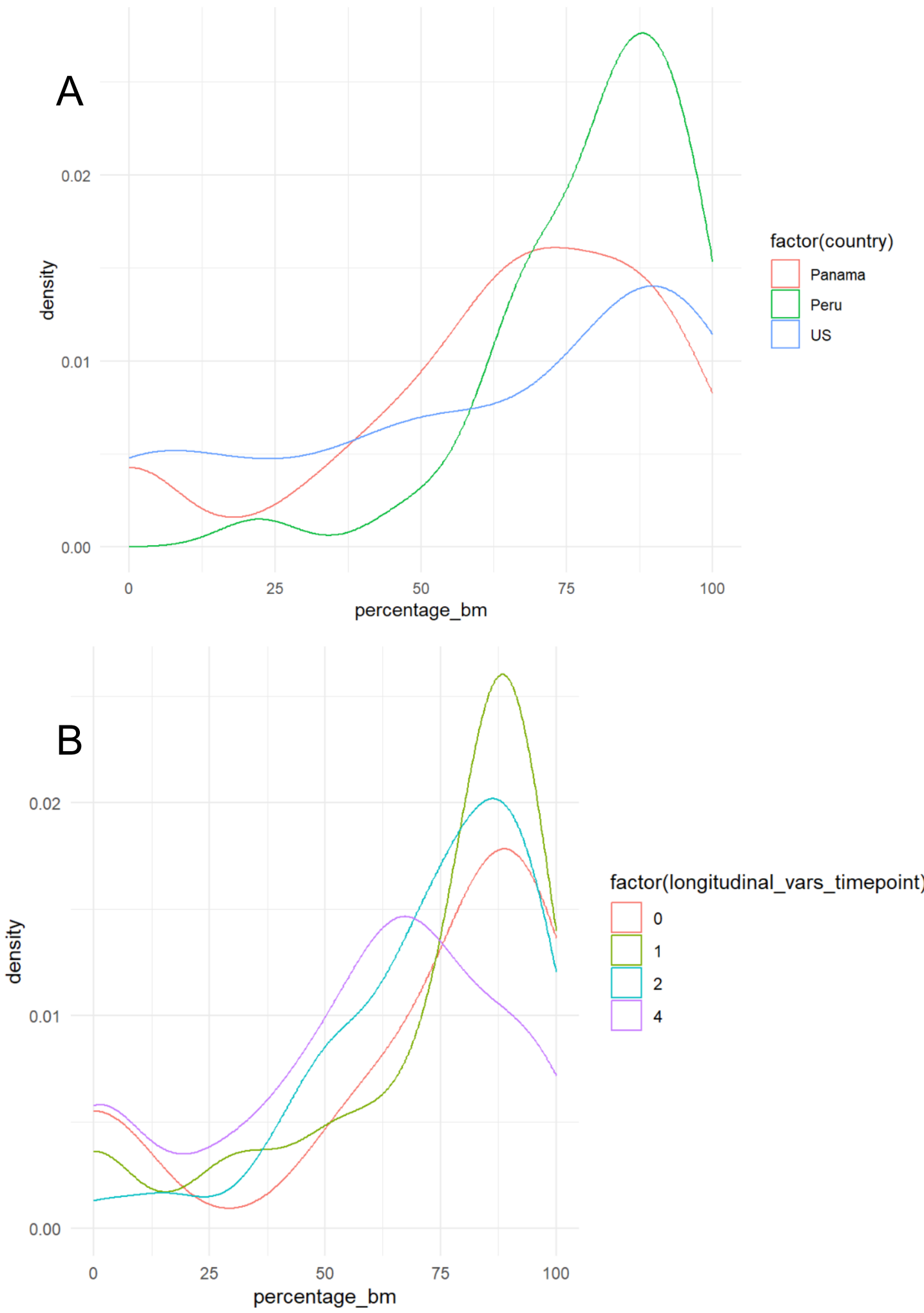

Supplementary Figure 1. Breastfeeding vs. formula percentages in infant diet. Density plots indicate the percentage formula vs. breastfed (**A**) between countries and (**B**) at each month of life (0 = M0, 1 = M1, 2 = M2, 4 = M4). Given the bimodal distribution in B, the decision was made to simplify the data model into binary categories rather than percentage. N = 337 infants.

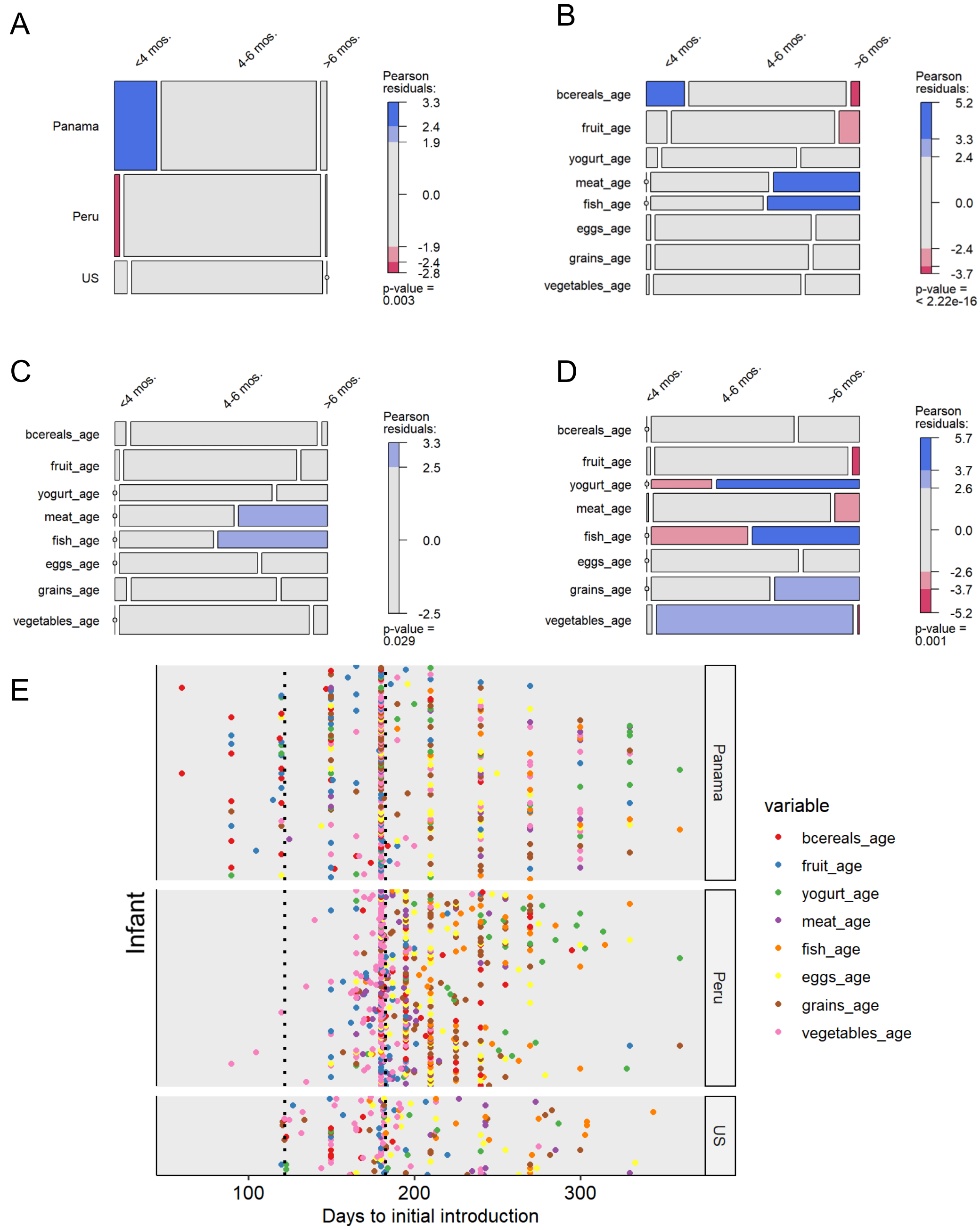

Supplementary Figure 2. Infant introduction to solid food differed between countries, but was rare prior to 4 months of age. For panels A-D, Pearson's Chi-squared tests evaluated associations. Cell colors map to Pearson residuals, showing positive (blue) or negative (red) deviations from expected frequencies; overall p-values are on the right. **(A)** Mosaic plot of differences in the introduction of solid food at each timepoint, indicating differences between countries (N = 337). (**B-D**) Mosaic plots indicating differences in the introduction of solid foods at each timepoint in Panama **(B)**, Peru **(C)**, and US **(D)**. **(E)** Map of specific food types introduced at each visit. The dotted line indicates 120 DOL (N=337).

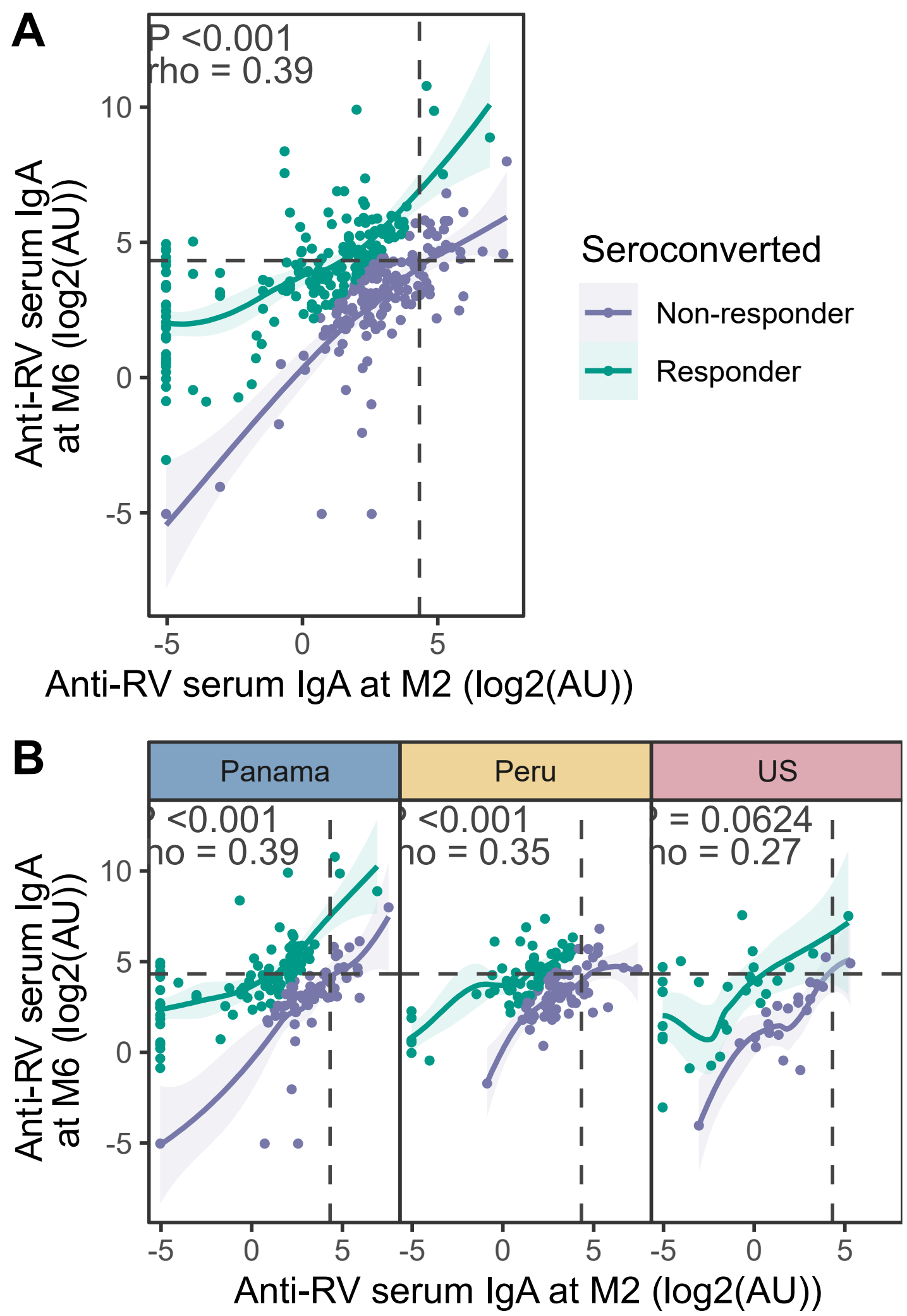

Supplementary Figure 3. Extended analysis of RV1 IgA response and sampling. (**A**) Comparison of pre- (M2) and post-vaccine (M6) anti-rotavirus serum IgA studywide (N=328). (**B**) Comparison of pre- and post-vaccine anti-rotavirus serum IgA in Panama (N = 147), Peru (N=131), and US (N=50). Dotted lines are placed at 20 AU, indicating the threshold of seropositivity. Statistical significance is calculated using Spearman's correlation between pre- and post- vaccine anti-rotavirus IgA in each country. Shaded regions around the trend lines represent 95% confidence intervals of loess curves.

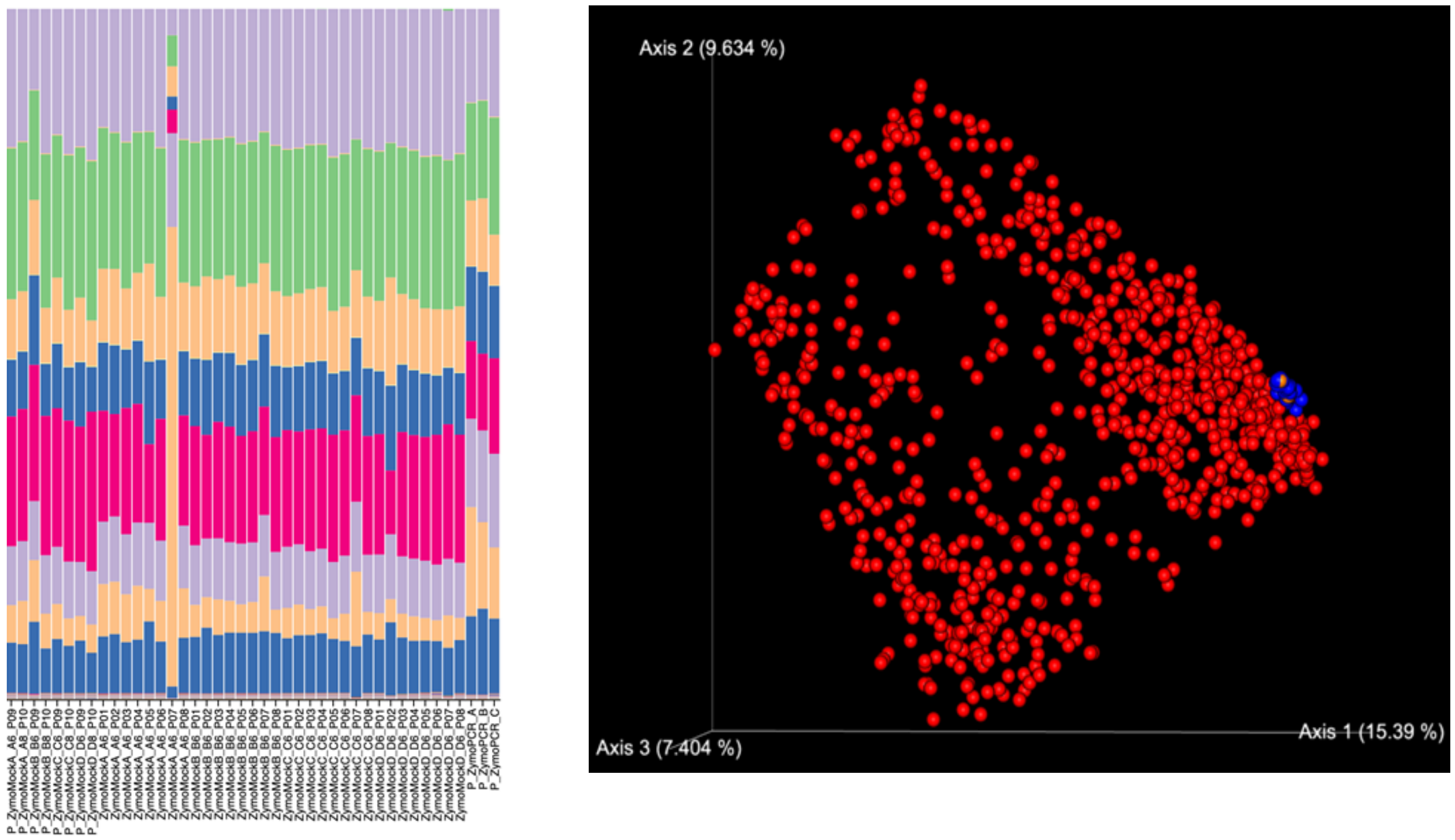

Supplementary Figure 4. Composition of positive controls included in metagenomic sequencing runs. (**A**) Community barplots of Zymo mock communities and mock DNA communities included in the study. (**B**) Unweighted UniFrac Emperor plot ordination of mock communities included in the study. Blue and yellow points indicate mock and mock-DNA positive controls, while red points indicate infant stool samples. The percentage of total variance explained by each of the three principal coordinate axes is indicated in parentheses on the respective axes.

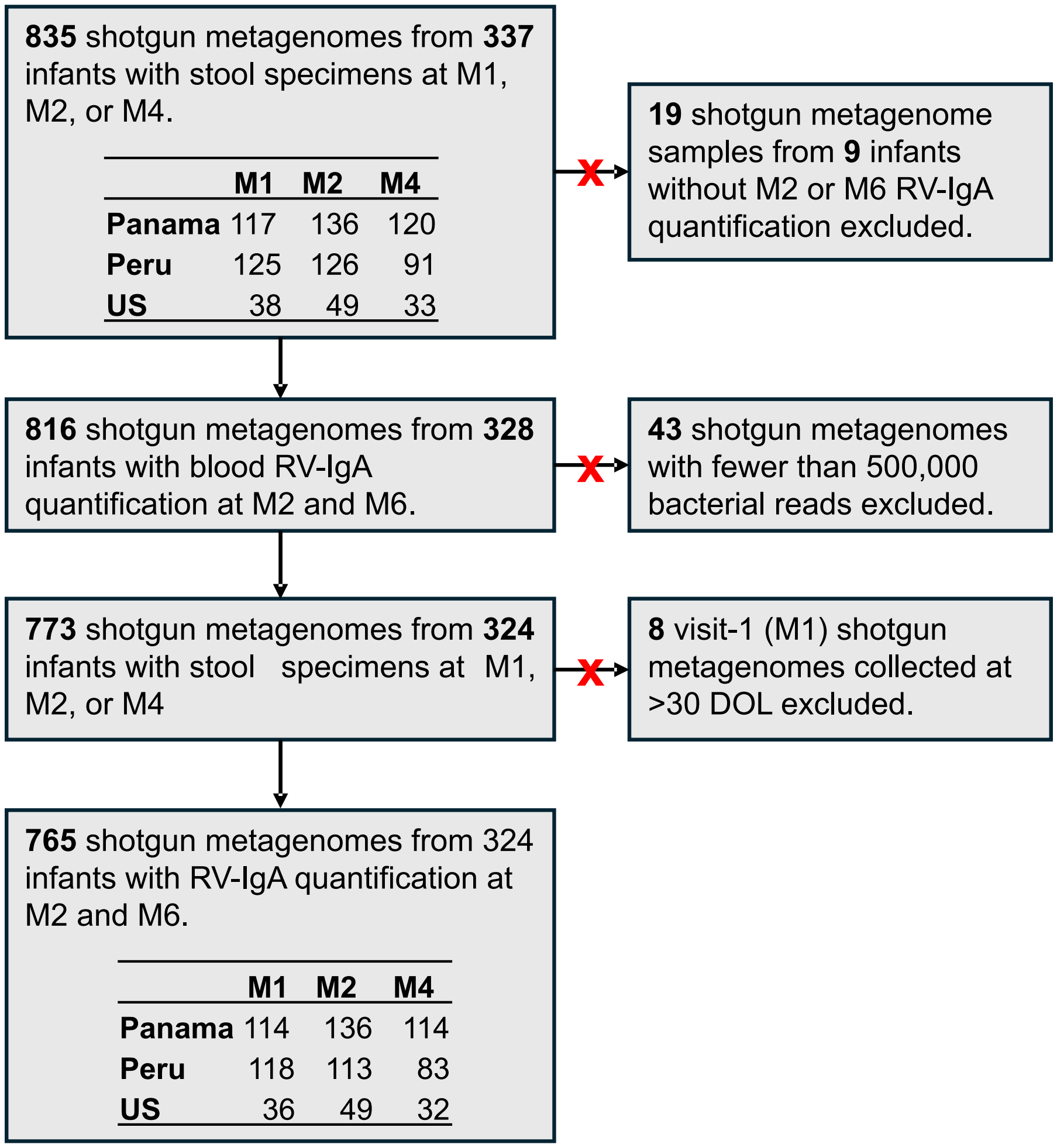

Supplementary Figure 5. Exclusion flowchart for shotgun metagenome samples and infants. This diagram describes the exclusion criteria implemented to cleanse metagenomic data and harmonize RV1 response and shotgun metagenomic datasets in this study.

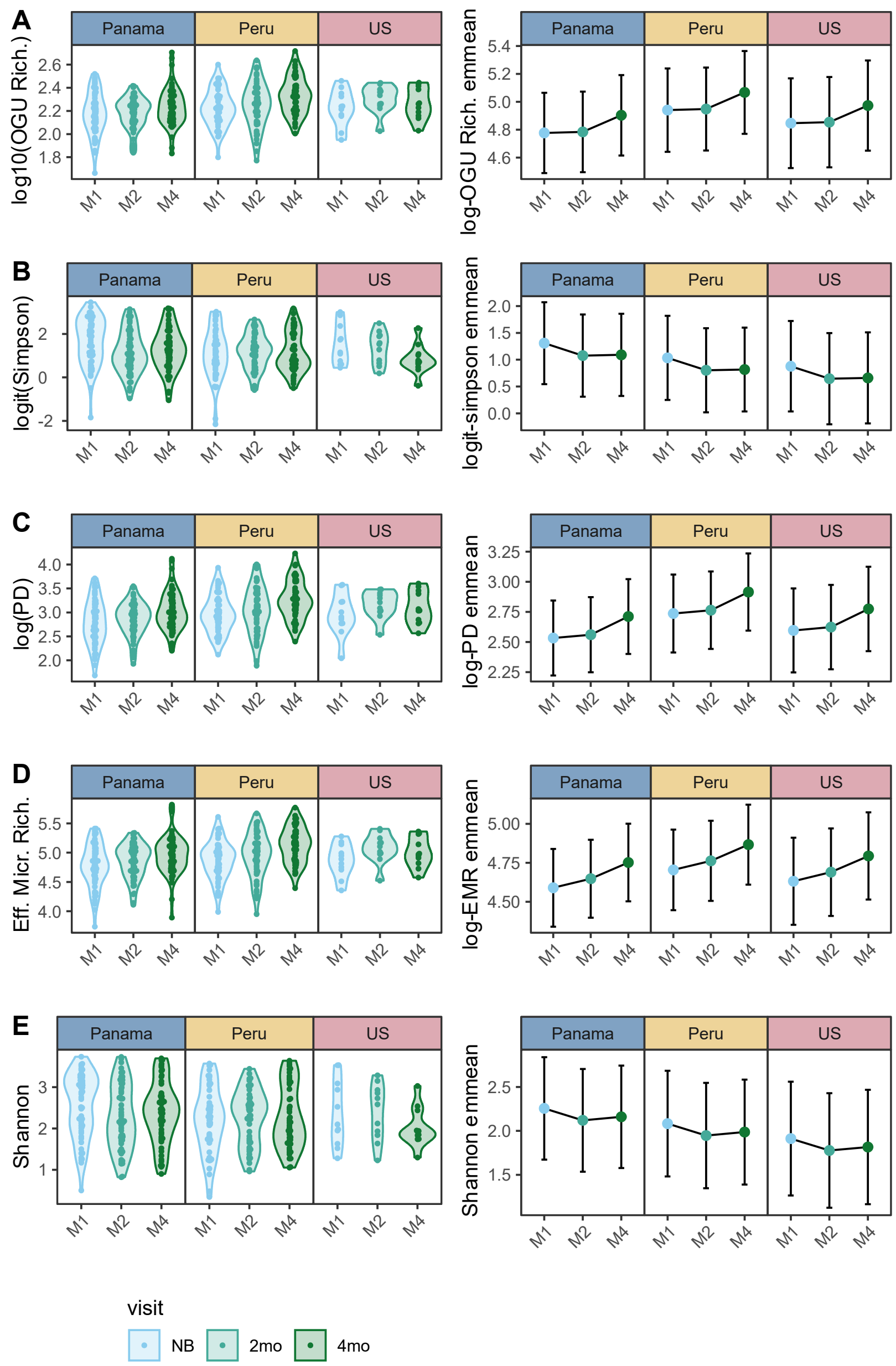

Supplementary Figure 6. Additional analysis of alpha diversity. Violin plots (left) illustrating the data distribution of raw diversity values, with individual sample data points overlaid. Emmeans (right) of the diversity index. Central points denote the model-adjusted estimated marginal means, and error bars represent the corresponding 95% confidence intervals (N = 110). Panels refer to alpha diversity metrics: **(A)** log10-OGU richness per country and timepoint, **(B)** Simpson evenness, **(C)** Faith's Phylogenetic Diversity, **(D)** Effective Microbial Richness, and **(E)** Shannon diversity.

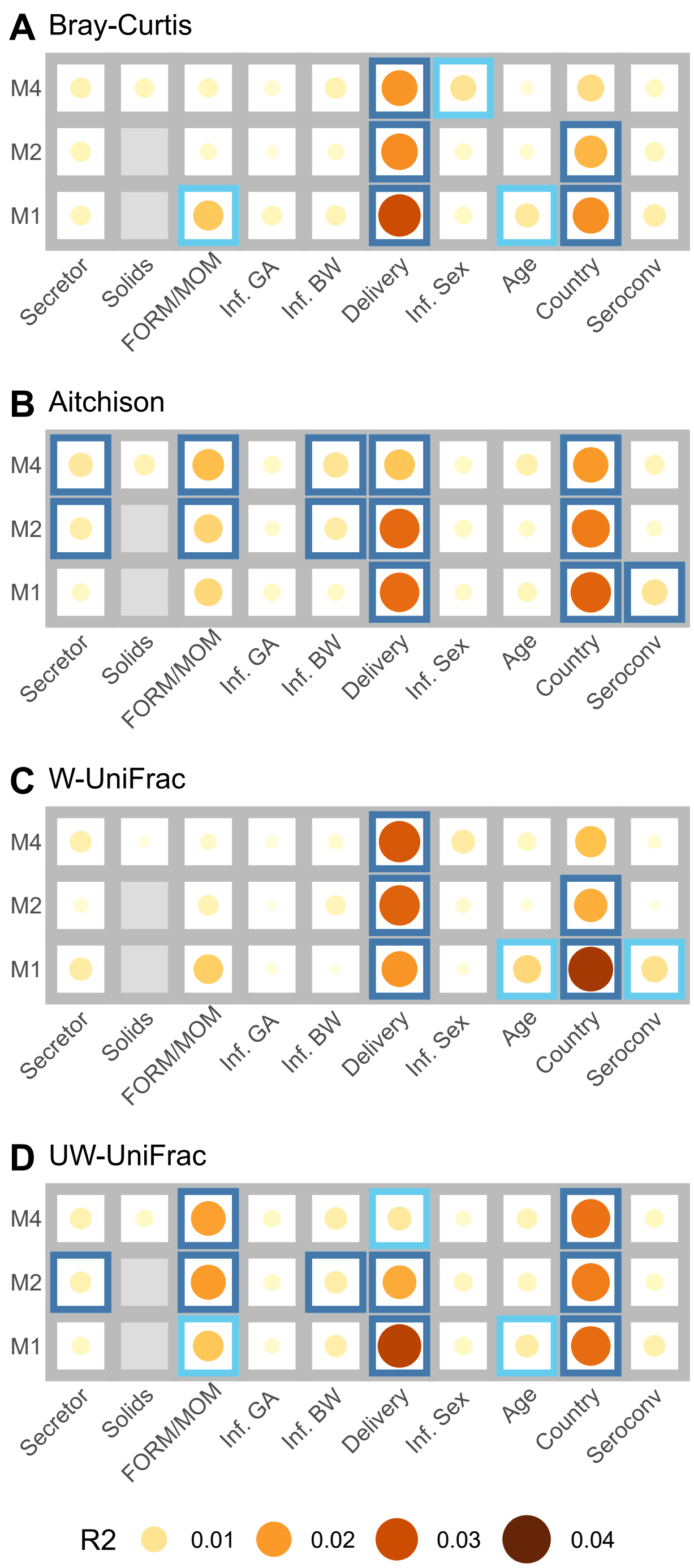

Supplementary Figure 7. Additional analysis of beta diversity. Panels A-D indicate the statistical significance and effect magnitude associated with each variable according to PERMANOVA cross-sectionally at M1 (N = 193), M2 (N =268), and M4 (N =188). The size and color intensity of the inner circles represent the effect size with larger, darker orange circles indicating a stronger effect on community composition. P < 0.1 are indicated by light blue boxes, P < 0.05 are indicated by dark blue boxes, and grey boxes indicate non-significant factors.

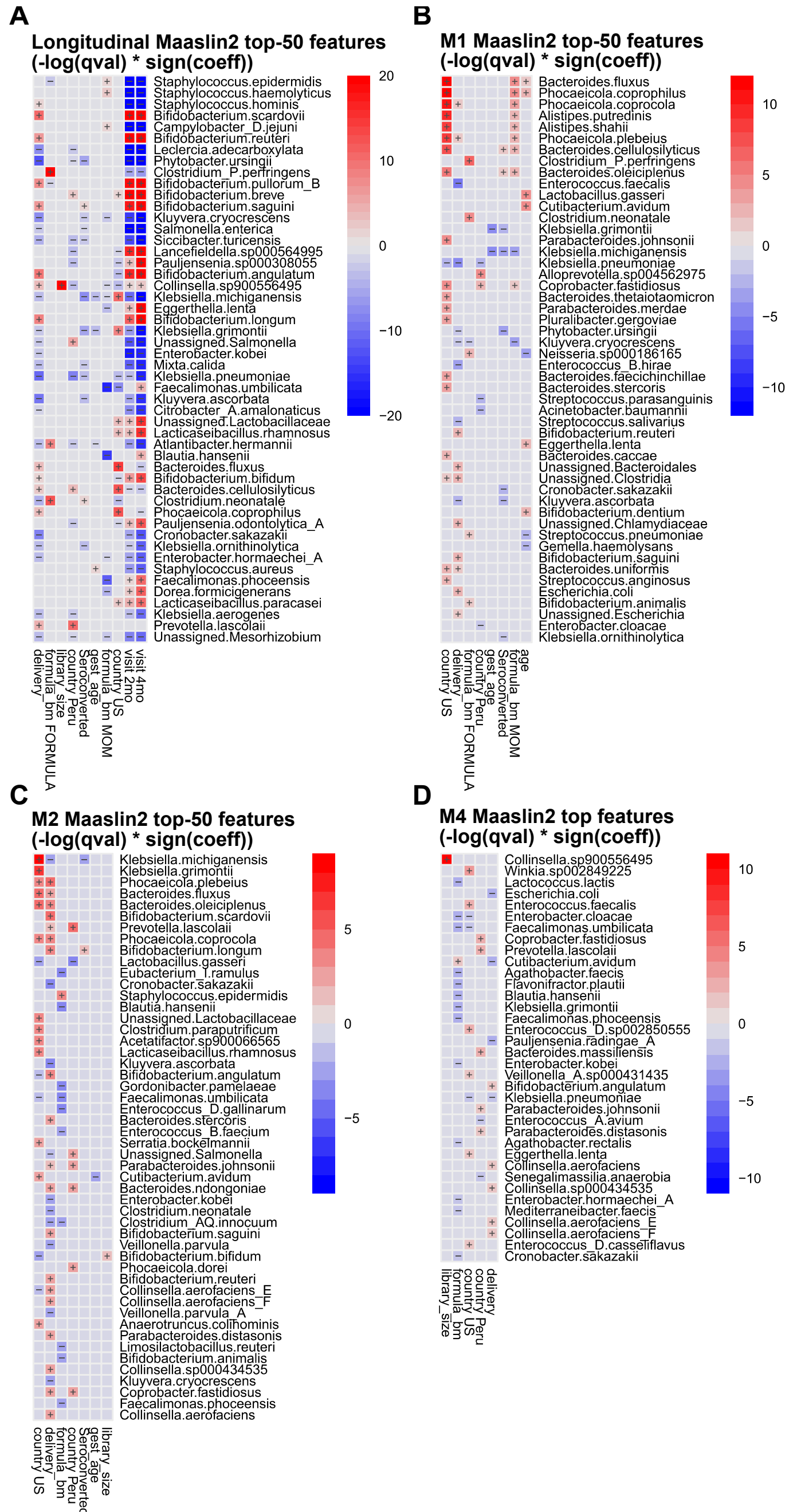

Supplementary Figure 8. Additional differentially abundant taxa in this cohort. Differentially abundant taxa according to longitudinal **(A)** and cross-sectional **(B-D)** MaAsLin2 analyses (N=. Panels reflect each differential abundance analysis performed here: **(A)** using M1, M2, and M4 timepoints in a mixed-effects model, using age in months as a covariate **(B)** using only samples from the M1 timepoint using age in days as a covariate **(C)** using only samples M2 timepoint, with age in days **(D)** using samples from the M4 timepoint, using age in days as a covariate. Red and blue gradients denote positive and negative associations, respectively, with darker color intensities reflecting smaller FDR-adjusted q-values; significant or near-significant cells are marked with **+** or **-**, while light gray cells indicate non-significance.

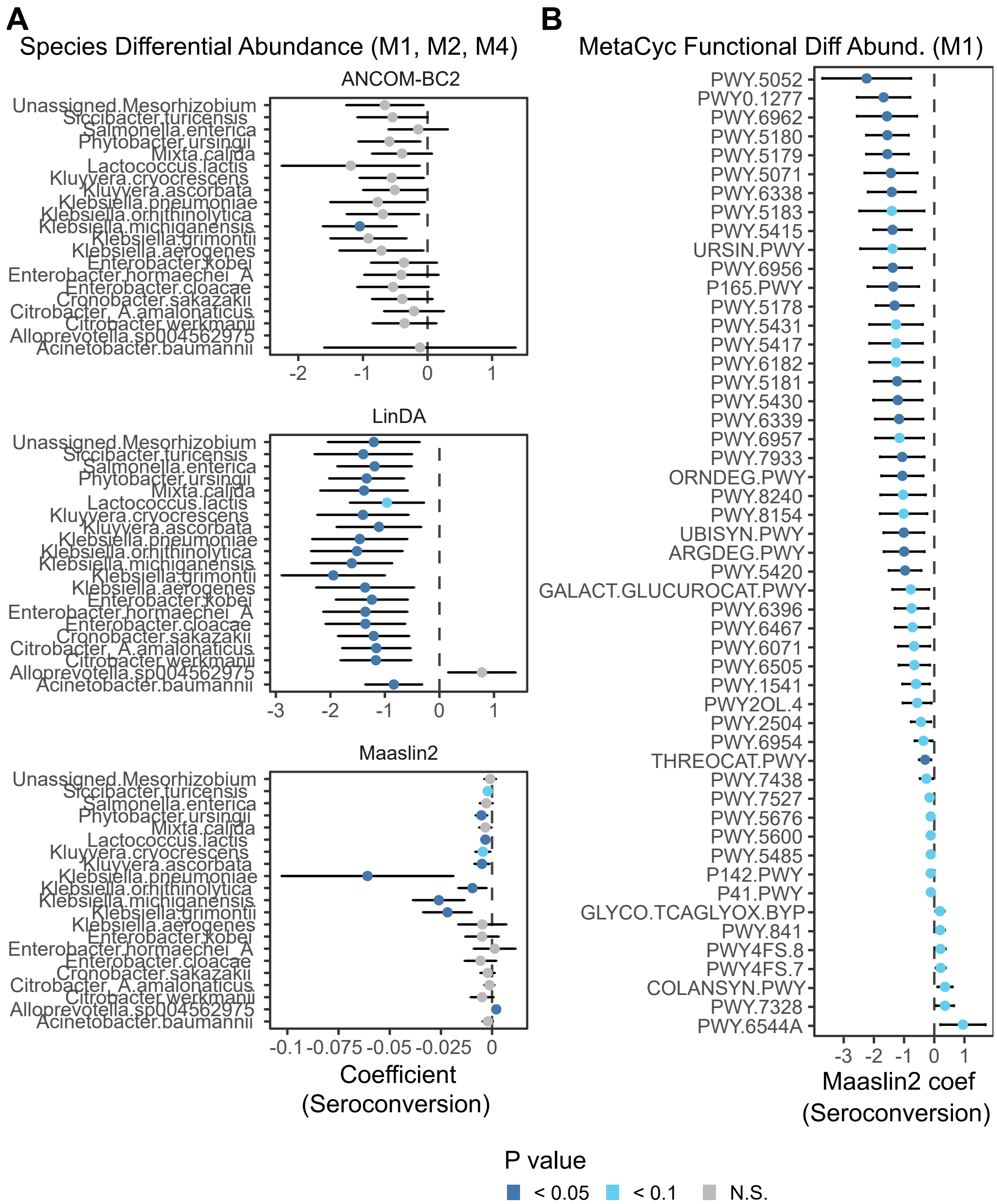

Supplementary Figure 9. Extended differential abundance analysis. (A) Differential abundance analysis (DAA) carried out using a longitudinal mixed-effects models via MaAsLin2[3], ANCOM-BC2[4], and LinDA[5] (N=324 infants; 765 samples). *Klebsiella grimontii*, *Klebsiella ornithinolytica*, *Klebsiella pneumoniae*, and *Phytobacter ursingii* are enriched among RV1 non-responders according to both LinDA and MaAsLin2. All timepoints were included. *Klebsiella michiganensis* are enriched in RV1 non-seroconverters according to all three DAA methods. (**B**) Full MetaCyc differential abundance results for the M1 timepoint. Central points and error bars represent the estimated effect size of seroconversion and confidence intervals, respectively, with point colors indicating P-value significance.

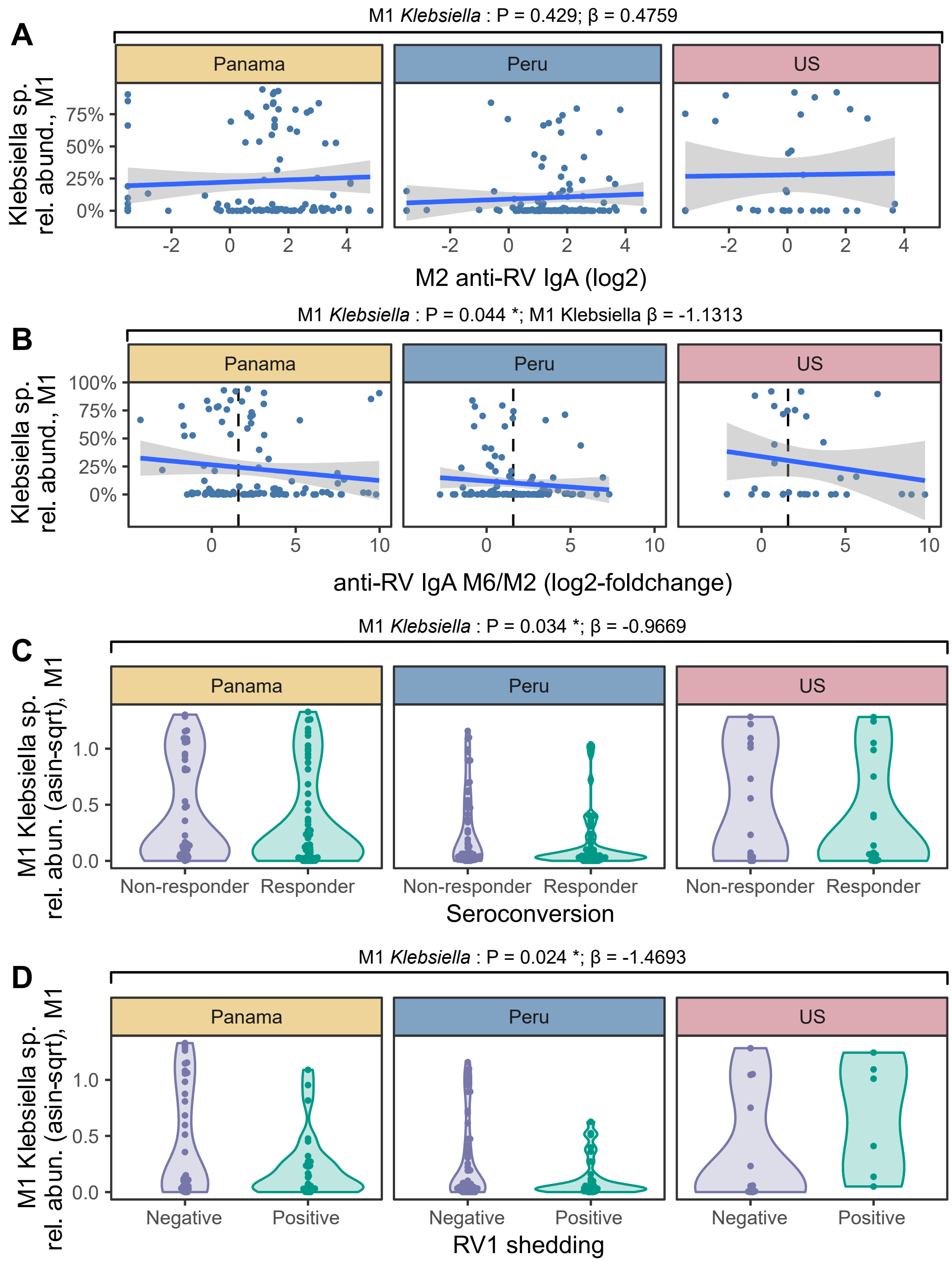

Supplementary Figure 10. Additional analysis of *Klebsiella* relative abundance and pre- and post-RV vaccine findings (**A**) anti-RV IgA pre-vaccine. (**B**) anti-RV foldchange M6/M2, with dotted lines drawn at 3-fold IgA difference M6 / M2, indicating seroconversion. (**C**) RV1 seroconversion, (**D**) and RV1 shedding detection at M4. Statistics indicated above each panel were calculated via linear (pre-vaccine IgA and IgA foldchanges N=252) or logistic (seroconversion, N=252; and shedding, N=172) regression + Wald test including country and untransformed M1 *Klebsiella* sum relative abundance as regression terms. Overall statistical associations, including P-values and beta coefficients are displayed at the top of each respective panel.

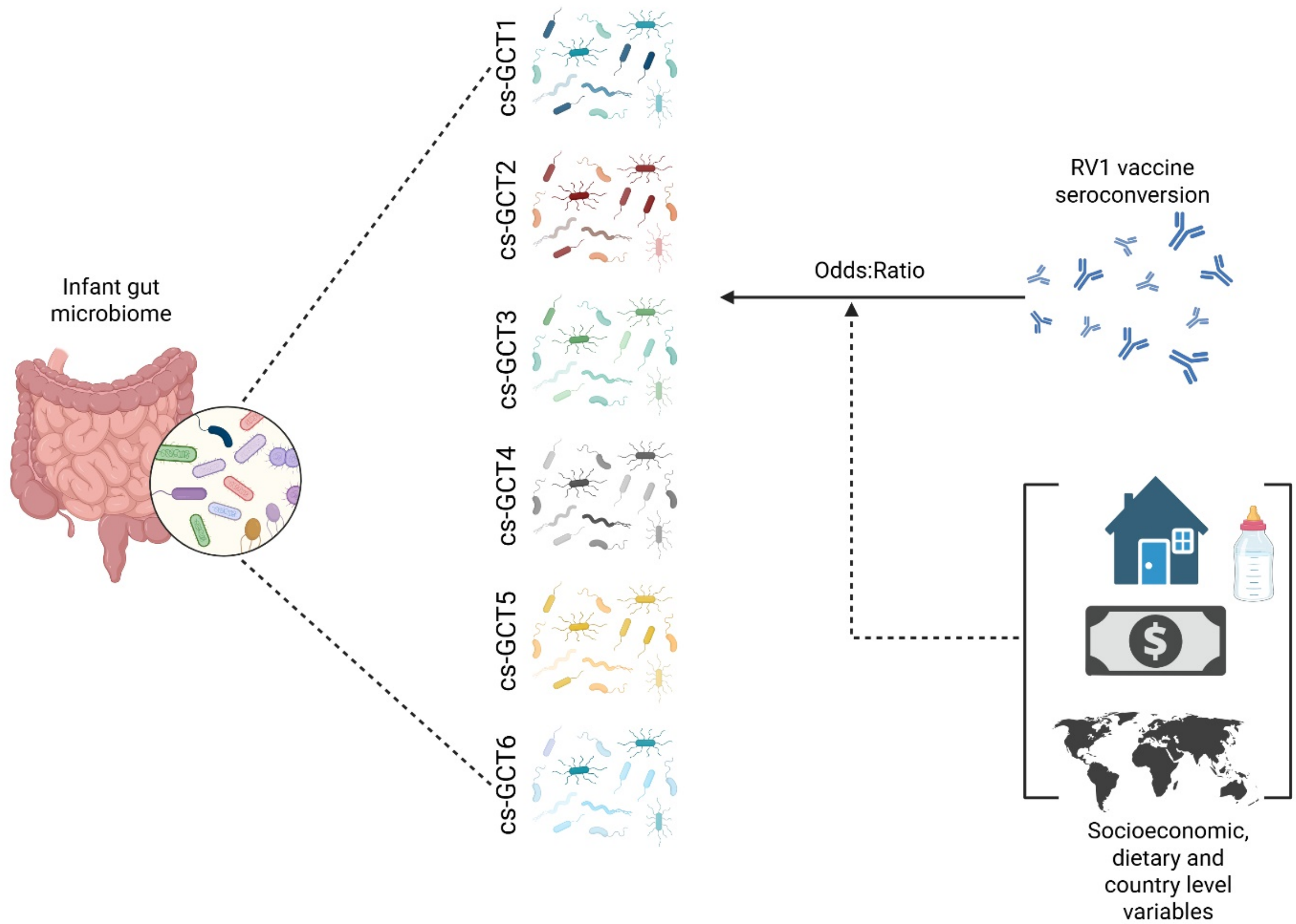

Supplementary Figure 11. Cross-sectional gut community type (cs-GCT) analysis workflow diagram. For this analysis, the odds ratios computed reflect marginal odds of association between RV1 vaccine seroconversion and each cs-GCT, while considering contributions due to demographic, dietary, and other variables that modify the infant gut microbiome. Created in BioRender. Nicols, A. (2026) https://BioRender.com/c3aw0bd.

A

	Seroconversion NR	Seroconversion R (%)	Seropositivity (M2) -	Seropositivity (M2) + (%)	Shedding (M4) -	Shedding (M4) + (%)	Seropositivity (M6) -	Seropositivity (M6) + (%)
cs-GCT1	31	37 (54.4%)	64	4 (5.9%)	28	20 (41.7%)	52	16 (23.5%)
cs-GCT2	25	25 (50.0%)	41	9 (18.0%)	24	12 (33.3%)	32	18 (36.0%)
cs-GCT3	24	18 (42.9%)	38	4 (4.0%)	22	4 (15.4%)	35	7 (16.7%)
cs-GCT4	20	19 (51.3%)	31	8 (20.5%)	15	12 (44.4%)	18	21 (53.8%)
cs-GCT5	13	17 (56.7%)	25	5 (16.7%)	9	10 (52.6%)	19	11 (36.7%)
cs-GCT6	6	17 (73.9%)	22	1 (4.3%)	11	5 (31.2%)	13	10 (43.5%)
	P = 0.265; χ²=6.45		P = 0.121; χ²=8.72		P = 0.119; χ²=8.75		P = 0.004*; χ²=17.04	

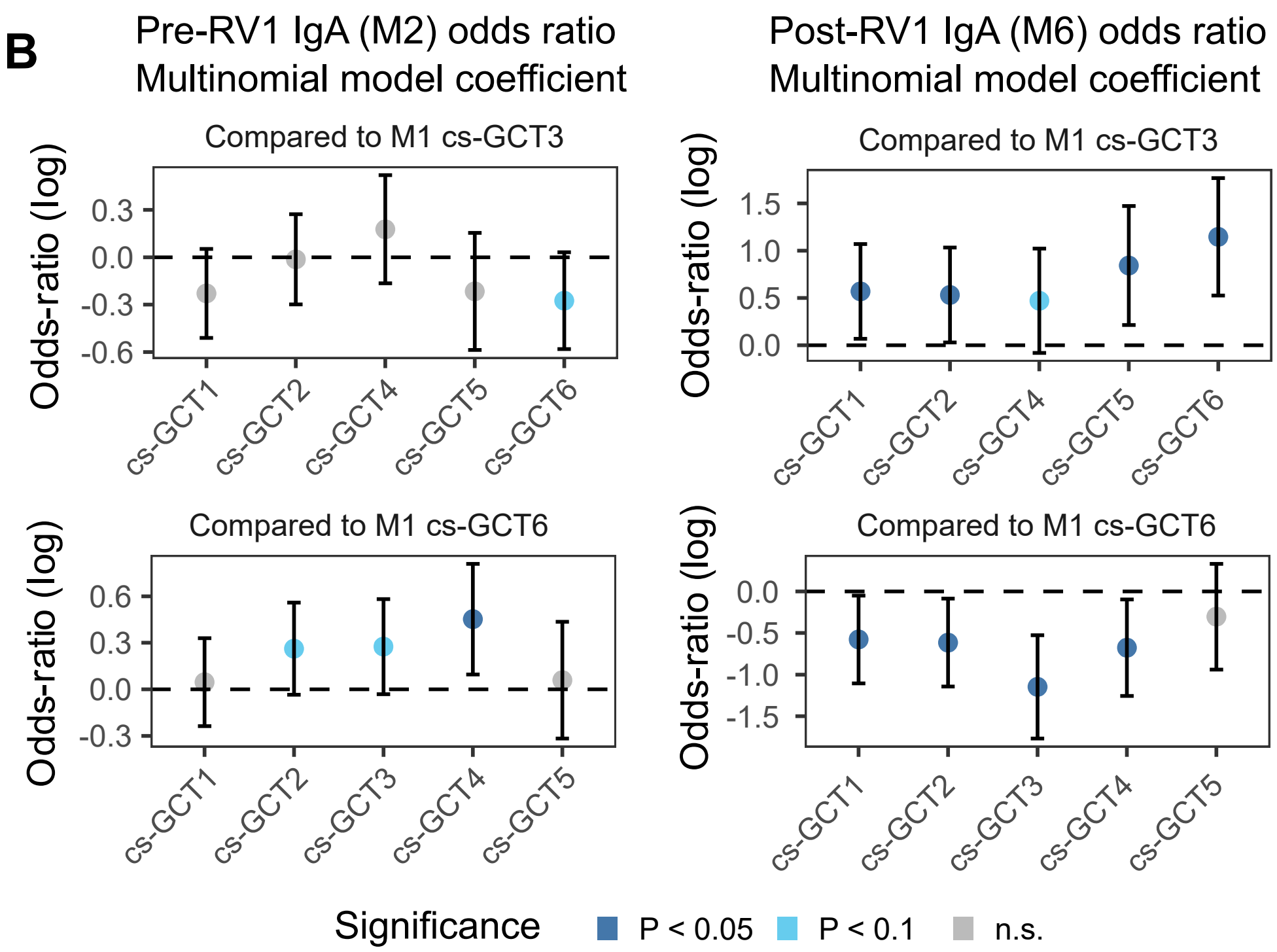

Supplementary Figure 12. Extended analysis of RV1 response among infant M1 gut types. (**A**) Additional response tabulations per cs-GCT. Significance was assessed via one-sided, upper-tailed Chi-squared test (N = 252, seroconversion and seropositivity; N =172, shedding). (**B**) Additional cs-GCT multinomial model coefficients for pre- and post-RV1 anti-RV IgA titers (N=252). Significance was assessed via uncorrected Wald test. Bars indicate 95% confidence intervals as defined by mean +/- 2SE, while points indicate the mean.

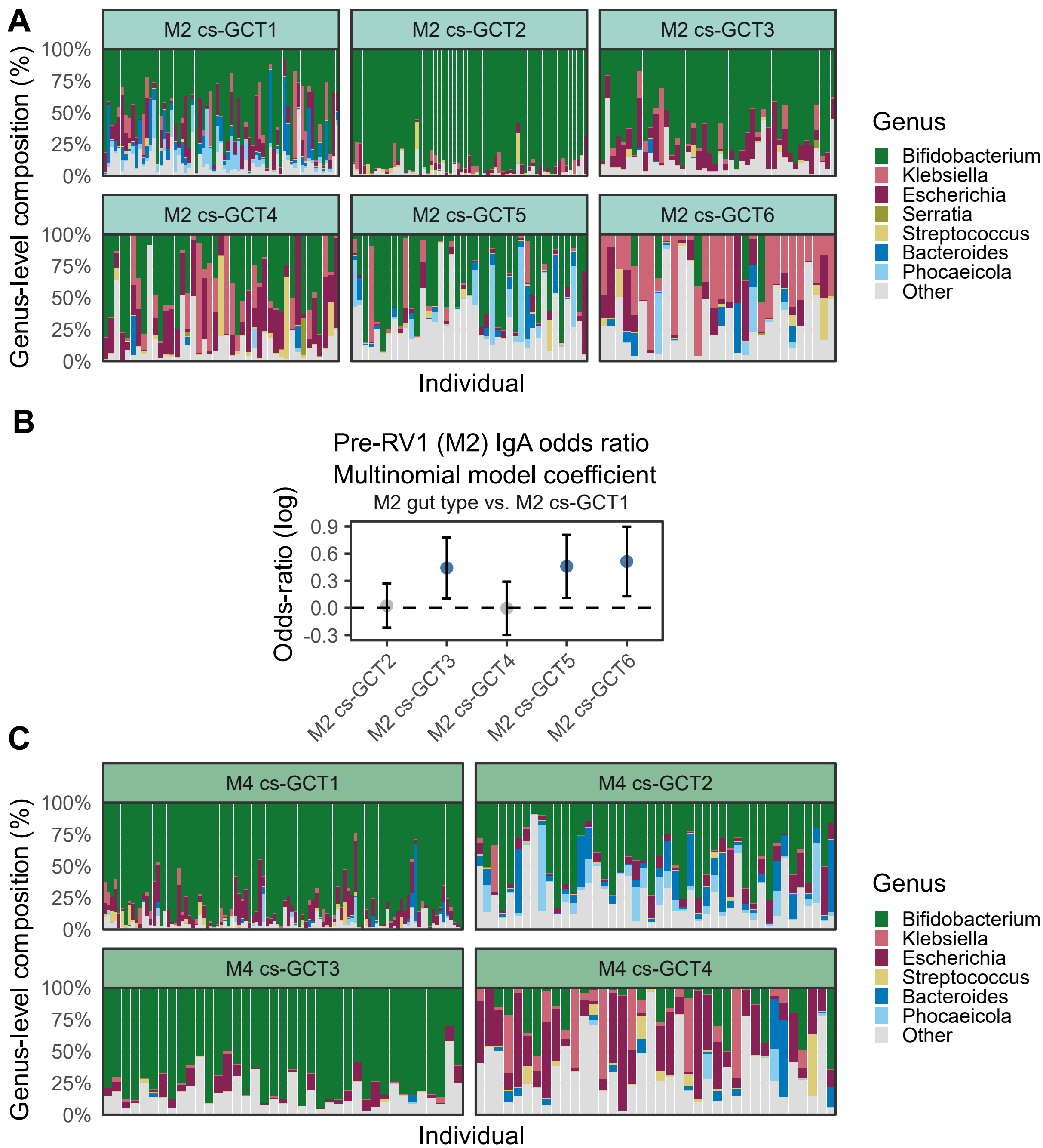

Supplementary Figure 13. Analysis of M2 and M4 cs-GCTs. (**A**) Genus-level relative composition (%) of the month 2 (M2) infant gut microbiome. Individual samples (vertical bars) are grouped into six cross-sectional gut community types (cs-GCT1-6). (**B**) Association between M2 cs-GCT and baseline pre-vaccination anti-rotavirus IgA based on multinomial logistic regression (N = 268). Points indicate the mean log-odds associated with each cs-GCT, and error bars extend to +/- 2SEM. (**C**) Genus-level composition of M4 cs-GCTs. Per multinomial logistic regression (N = 188), no significant association was identified between M4 community composition and any rotavirus or RV1 response measure.

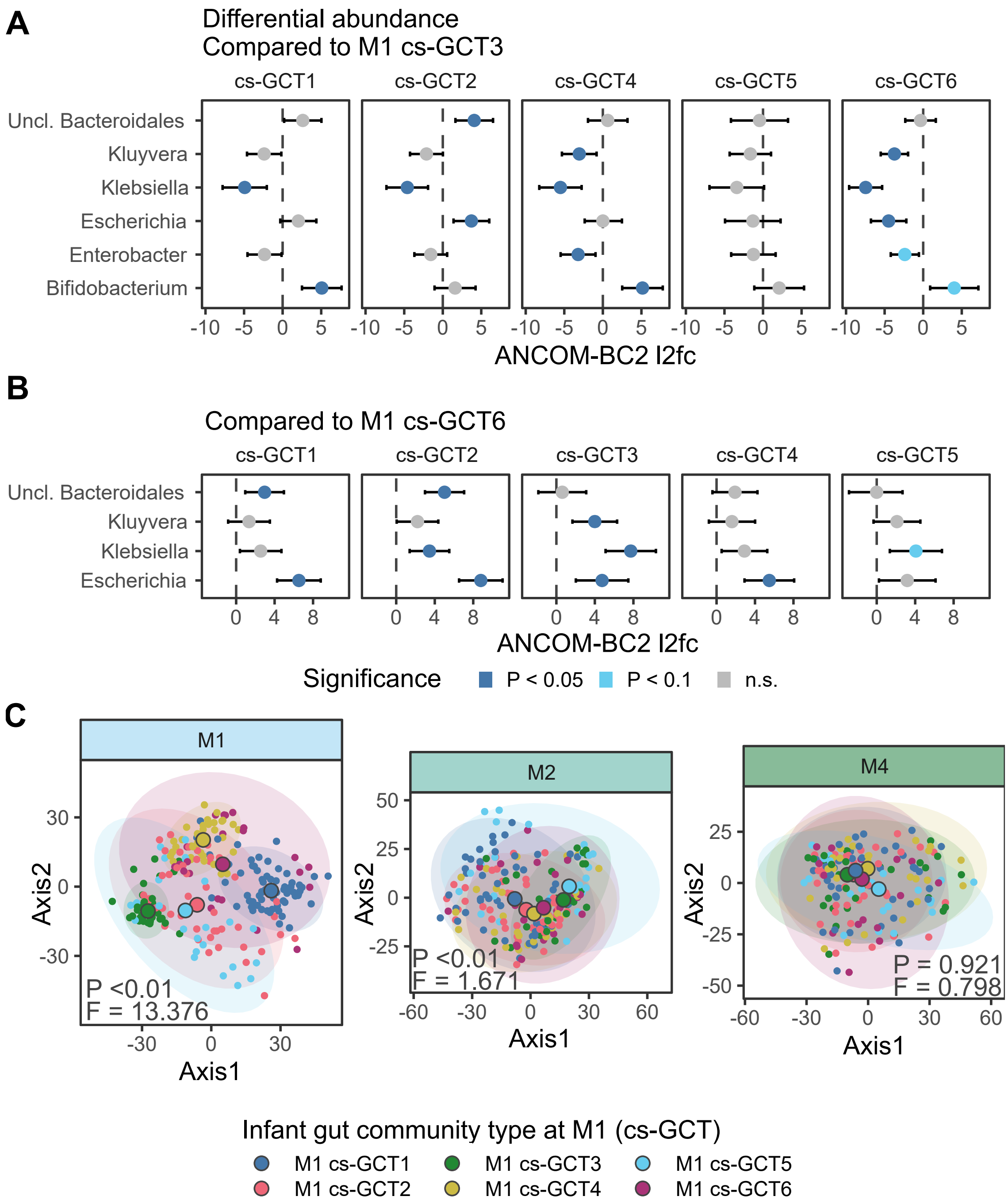

Supplementary Figure 14. Variation in gut ecology among infant M1 gut types. (**A**) Full differential abundance results as compared to M1 cs-GCT3 (N=252). Points indicate the estimated mean and error bars extend to +/- 2 SE of the log2-foldchange. (**B**) Full differential abundance as compared to M1 cs-GCT6. (**C**) Aitchison-PCoA ordination comparing evolution of M1 cs-GCTs from M1 to M4. Statistics shown indicate sequential PERMANOVA at each timepoint with M1 cs-GCT as the final term (N=252, N=223, N=169).

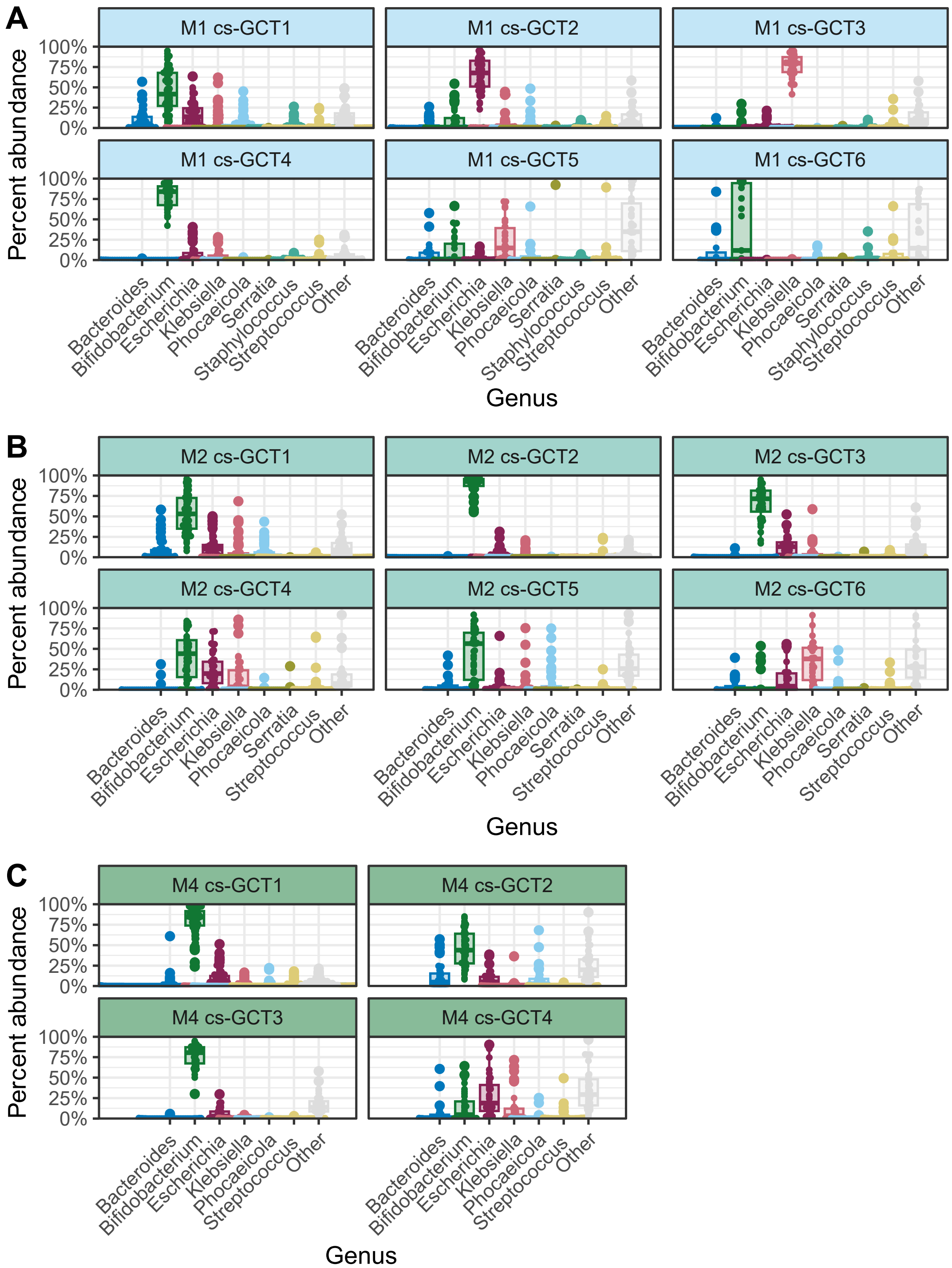

Supplementary Figure 15. Abundance of select taxa within each gut type. Boxplots showing percent abundances of major taxa in gut types at each timepoint: (**A**) M1, (**B**) M2, (**C**) M4. The central horizontal line within each box indicates the median percent abundance, while the lower and upper hinges represent the interquartile range. Differential abundance analysis and is available for M1 in **Supplementary Figure 8** and **Supplementary Figure 14**.

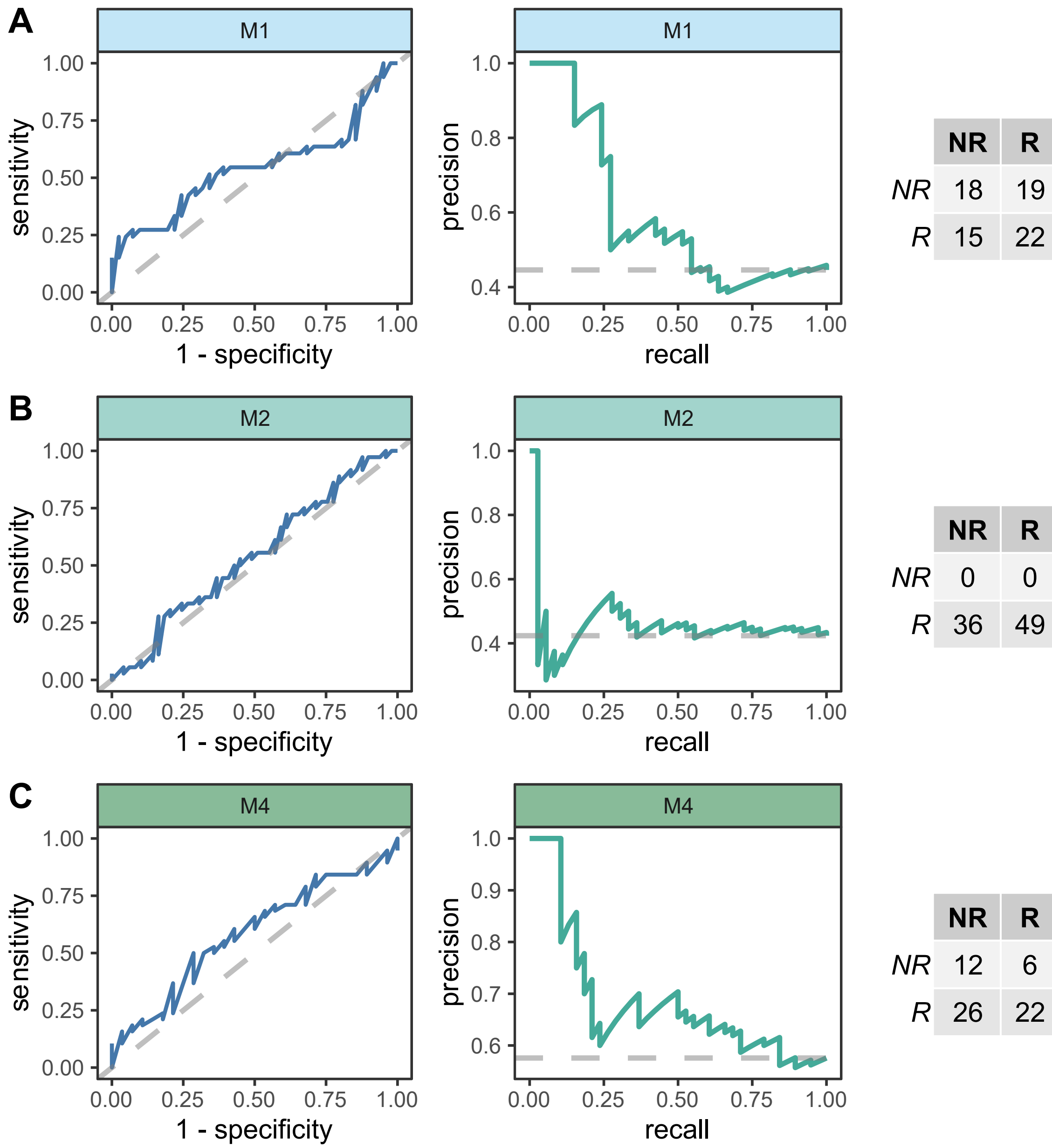

Supplementary Figure 16. RV1 seroconversion machine learning model performance. Receiver-operating characteristic curves, precision recall curves, and tables showing Seroconversion (R = Responder, NR = Non-responder) predictions (y-axis) and ground truth (x-axis) for holdout data at (**A**) M1, (**B**) M2, and (**C**) M4 respectively. The solid blue line plots sensitivity (true positive rate) against 1 - specificity (false positive rate) across various threshold settings. Dashed grey lines represent the expected performance of a naïve classifier.

Supplementary References

1. Reitmeier, S. *et al*. Handling of spurious sequences affects the outcome of high-throughput 16S rRNA gene amplicon profiling. *ISME Commun*. **1**, 31 (2021).
2. Fox, J. & Weisberg, S. *An R Companion to Applied Regression*. (SAGE Publications, 2011).
3. Mallick, H. *et al*. Multivariable association discovery in population-scale meta-omics studies. *PLoS Comput. Biol*. **17**, e1009442 (2021).
4. Lin, H. & Peddada, S. D. Analysis of compositions of microbiomes with bias correction. *Nat. Commun*. **11**, 1–11 (2020).
5. Zhou, H., He, K., Chen, J. & Zhang, X. LinDA: linear models for differential abundance analysis of microbiome compositional data. *Genome Biol*. **23**, 1–23 (2022).
