# Supplementary material for: Klebsiella-dominant gut communities are associated with impeded oral rotavirus vaccine response in infants
Source: Nat Commun. 2026 Oct 15;17:10783. doi: 10.1038/s41467-026-78148-x (PMC13672339; doi:10.1038/s41467-026-78148-x)
Supplement: Supplementary file 2 [file 41467_2026_78148_MOESM7_ESM.pdf]

Corresponding author(s): Pia S. Pannaraj, MD, MPH

Last updated by author(s): Aug 25, 2026

Reporting Summary

Nature Portfolio wishes to improve the reproducibility of the work that we publish. This form provides structure for consistency and transparency in reporting. For further information on Nature Portfolio policies, see our Editorial Policies and the Editorial Policy Checklist.

Statistics

For all statistical analyses, confirm that the following items are present in the figure legend, table legend, main text, or Methods section.

n/a	Confirmed	
☐	☒	The exact sample size (*n*) for each experimental group/condition, given as a discrete number and unit of measurement
☐	☒	A statement on whether measurements were taken from distinct samples or whether the same sample was measured repeatedly
☐	☒	The statistical test(s) used AND whether they are one- or two-sided<br>*Only common tests should be described solely by name; describe more complex techniques in the Methods section.*
☐	☒	A description of all covariates tested
☐	☒	A description of any assumptions or corrections, such as tests of normality and adjustment for multiple comparisons
☐	☒	A full description of the statistical parameters including central tendency (e.g. means) or other basic estimates (e.g. regression coefficient) AND variation (e.g. standard deviation) or associated estimates of uncertainty (e.g. confidence intervals)
☐	☒	For null hypothesis testing, the test statistic (e.g. *F*, *t*, *r*) with confidence intervals, effect sizes, degrees of freedom and *P* value noted<br>*Give P values as exact values whenever suitable.*
☒	☐	For Bayesian analysis, information on the choice of priors and Markov chain Monte Carlo settings
☐	☒	For hierarchical and complex designs, identification of the appropriate level for tests and full reporting of outcomes
☐	☒	Estimates of effect sizes (e.g. Cohen's *d*, Pearson's *r*), indicating how they were calculated

Our web collection on statistics for biologists contains articles on many of the points above.

Software and code

Policy information about availability of computer code

Data collection	Metadata (clinical data) was collected from participants and recorded in RedCap. Metagenome sequences were generated on Illumina NovaSeq X Plus at UC San Diego's Institute for Genomic Medicine.
Data analysis	R version 4.4.0 was used to analyze data. R scripts are available at the github repository PIALabUCSD/rv1-metagenomics-2025

For manuscripts utilizing custom algorithms or software that are central to the research but not yet described in published literature, software must be made available to editors and reviewers. We strongly encourage code deposition in a community repository (e.g. GitHub). See the Nature Portfolio guidelines for submitting code & software for further information.

Data

Policy information about availability of data

All manuscripts must include a data availability statement. This statement should provide the following information, where applicable:

- Accession codes, unique identifiers, or web links for publicly available datasets
- A description of any restrictions on data availability
- For clinical datasets or third party data, please ensure that the statement adheres to our policy

Raw shotgun metagenomic data generated here are available on the NCBI Sequence Read Archive under the BioProject accession number, PRJNA1404625 [http://www.ncbi.nlm.nih.gov/bioproject/PRJNA1404625]. Source data for each figure are provided with this paper. Sample metadata are provided in Supplementary Table 1.
Code used for this analysis is available via the GitHub repository PIALabUCSD/rv1-metagenomics-2025 [github.com\PiaLabUCSD\rv1-metagenomics-2025].

Research involving human participants, their data, or biological material

Policy information about studies with human participants or human data. See also policy information about sex, gender (identity/presentation), and sexual orientation and race, ethnicity and racism.

Reporting on sex and gender	Sex data was collected on all participating infants as reported by their parents. Data was included in the analyses and reported in the manuscript
Reporting on race, ethnicity, or other socially relevant groupings	Data on race and ethnicity were collected all on participants as reported by their parents. The data was included in the analyses and reported in the manuscript
Population characteristics	Infants <2 months were recruited for participation. All demographic and clinical characteristics of the infants and their mothers are reported in Table 1 of the manuscript. These factors were included in the analyses.
Recruitment	Healthy mother and infant (born ≥35 weeks' gestation) pairs were enrolled prior to the infant's first rotavirus vaccine dose at Children's Hospital Los Angeles (CHLA; Los Angeles, USA), Kaiser Permanente (Los Angeles, USA), Universidad Peruana Cayetano Heredia (UPCH; Lima, Peru), and Hospital del Niño Doctor Jose Renan Esquivel (Panama City, Panama), using a convenience recruitment strategy. These hospitals serve patients wide socioeconomic background. Healthy patients were recruited so that we could evaluate immune responses to vaccines. Exclusion criteria included being born <35 weeks' gestation or any immunosuppressive disease or medication exposure.
Ethics oversight	Institutional review boards at CHLA, Kaiser, UPCH, and Hospital del Niño each approved the study at their respective sites. Informed consent was obtained.

Note that full information on the approval of the study protocol must also be provided in the manuscript.

Field-specific reporting

Please select the one below that is the best fit for your research. If you are not sure, read the appropriate sections before making your selection.

☒ Life sciences ☐ Behavioural & social sciences ☐ Ecological, evolutionary & environmental sciences

For a reference copy of the document with all sections, see nature.com/documents/nr-reporting-summary-flat.pdf

Life sciences study design

All studies must disclose on these points even when the disclosure is negative.

Sample size	Rotavirus vaccine seroconversion rates by country were used to determine sample size. Based on an average 90% RV vaccine seroconversion in high income countries, 74% seroconversion in Panama,23 and 66% seroconversion in Peru, power = 0.8, and α = 0.05, a sample size of 255 infants should be included in the study to evaluate differences between vaccine responders and non-responders by country. To account for longitudinal subject retention of 80% at 6 months, we aimed to enroll until reaching a total of 330 infants who complete the pre-vaccination visit. Our final dataset included 324 infants from whom we collected pre- and post-vaccination blood samples and stool for metagenomic analysis that passed quality filtering.
Data exclusions	Infants were excluded if they did not complete study visits or obtain the rotavirus vaccine per the recommended vaccination schedule. From the 328 infants who met those criteria, we generated and sequenced shotgun metagenomes from 816 stool samples. Of those, 51 samples and 4 infants were excluded following quality filtering (Supplementary Figure 4), culminating in a dataset of 765 metagenome samples from 324 infants.
Replication	All ELISAs performed for antibody levels were performed in duplicate. Metagenomics analyses were performed using multiple methods to ensure the same conclusions.
Randomization	This was an observational study to evaluate metagenomic factors taht may impact vaccine immunogenicity. The vaccine is routine vaccine recommended at 2 and 4 months of age per the national schedule in Panama, Peru and US. Thus, there was no randomization.
Blinding	There was no blinding. This was an observational study to evaluate metagenomic factors taht may impact vaccine immunogenicity. The

vaccine is routine vaccine recommended at 2 and 4 months of age per the national schedule in Panama, Peru and US.

Reporting for specific materials, systems and methods

We require information from authors about some types of materials, experimental systems and methods used in many studies. Here, indicate whether each material, system or method listed is relevant to your study. If you are not sure if a list item applies to your research, read the appropriate section before selecting a response.

Materials & experimental systems

n/a	Involved in the study
☐	☒ Antibodies
☒	☐ Eukaryotic cell lines
☒	☐ Palaeontology and archaeology
☒	☐ Animals and other organisms
☐	☒ Clinical data
☒	☐ Dual use research of concern
☒	☐ Plants

Methods

n/a	Involved in the study
☒	☐ ChIP-seq
☒	☐ Flow cytometry
☒	☐ MRI-based neuroimaging

Antibodies

Antibodies used: Anti-calprotectin antibodies were used as a part of the IDK® Calprotectin (MRP 8/14, S100A8/A9) ELISA Kit, supplied by Immundiagnostik AG (Catalog No. KR6967; Lot No. KR6967-241204).

Validation: This is a commercial sandwich ELISA kit utilizing proprietary monoclonal antibodies. The individual capture and detection antibody clones were not purchased or provided separately. The antibodies used in the commercial kit were validated by the manufacturer for the quantitative determination of human calprotectin in stool samples by sandwich ELISA. Manufacturer validation includes established performance characteristics, including analytical sensitivity, measurement range, intra- and inter-assay precision, and analytical specificity, as detailed in the product.

Clinical data

Policy information about clinical studies

All manuscripts should comply with the ICMJE guidelines for publication of clinical research and a completed CONSORT checklist must be included with all submissions.

Clinical trial registration: This was not a clinical trial.

Study protocol: This was not a clinical trial.

Data collection: Demographic and medical history data was collected at enrollment via interview questionnaires. Feeding history and updated medical history was also collected at each visit at birth and 1, 2, 4 and 6 months via interview questionnaire.

Outcomes: The outcome of interest was Rotavirus vaccine seroconversion, defined as a ≥3-fold change in pre vs. post-vaccination infant serum IgA.

Plants

Seed stocks: *Report on the source of all seed stocks or other plant material used. If applicable, state the seed stock centre and catalogue number. If plant specimens were collected from the field, describe the collection location, date and sampling procedures.*

Novel plant genotypes: *Describe the methods by which all novel plant genotypes were produced. This includes those generated by transgenic approaches, gene editing, chemical/radiation-based mutagenesis and hybridization. For transgenic lines, describe the transformation method, the number of independent lines analyzed and the generation upon which experiments were performed. For gene-edited lines, describe the editor used, the endogenous sequence targeted for editing, the targeting guide RNA sequence (if applicable) and how the editor was applied.*

Authentication: *Describe any authentication procedures for each seed stock used or novel genotype generated. Describe any experiments used to assess the effect of a mutation and, where applicable, how potential secondary effects (e.g. second site T-DNA insertions, mosiacism, off-target gene editing) were examined.*
