# Supplementary material for: Klebsiella-dominant gut communities are associated with impeded oral rotavirus vaccine response in infants
Source: Nat Commun. 2026 Oct 15;17:10783. doi: 10.1038/s41467-026-78148-x (PMC13672339; doi:10.1038/s41467-026-78148-x)
Supplement: Supplementary file 3 [file 41467_2026_78148_MOESM8_ESM.pdf]

nature portfolio

# Peer Review File

# Klebsiella-dominated gut communities are associated with impeded oral rotavirus vaccine response in infants

Corresponding Author: Dr Pia Pannaraj

**This file contains all reviewer reports in order by version, followed by all author rebuttals in order by version.**

Version 0:

Reviewer comments:

Reviewer #1

(Remarks to the Author)
This is an important work that integrates microbial and inflammatory measures with rotavirus vaccine (RVV) response data from infants across 3 diverse countries. Previous studies have were from single sites and focused on either microbiome community structure or biomarkers of environmental enteropathy, but not in the same study. Also the inclusion of machine learning methods to understand the complex data is novel. The noteworthy results are that Klebsiella species, particularly those with LPS biosynthetic genes were more abundant in infants that did not seroconvert. Higher fecal calprotectin concentrations was also associated with failure to seroconvert. In general the paper is well-written and there is enough detail provided for the work to be reproduced.

There are a few concerns or perhaps clarifications needed in the methods used:
1. Throughout the paper the authors measure a stool collect at 1 month of age, however, the methods state that stool samples were collected at 1-2 weeks and 2 months, but not 1 month. Which stool was used for the reported 1 month outcomes? Further, from Figure 1A, it appears that RVV shedding was assessed on the day of vaccination. Typically, one would assess this in the days/weeks after vaccination, once the vaccine virus has had a chance to replicate in the intestinal mucosa.

2. There is no mention of maternal antibody levels and their association with the seroconversion outcome. Several studies have shown that transplacentally-acquired antibody and human milk antibodies can block the vaccine virus replication, and decrease immunogenicity. If there are no maternal serum samples available for this analysis, this should be included as a limitation.

3. Diet is a complicated construct. The methods do not describe what what collected for the diet variable. It appears that it was breastfed/mixed/formula-fed in one area and breastfed/formula-fed in one of the results figures. Was this data collected at each visit? Instead of diet, that may be better described as breastfeeding status. Related to point 2 above, breastfeeding would not only influence microbiome composition, but would also influence seroconversion, if the maternal milk contains either adaptive or non-adaptive immune factors that block vaccine take. How was this considered in the models used?

4.In Figure 2B, country, gestational age at birth, child age, and receipt of breastmilk/formula were the factors that resulted in the greatest differences in diversity. However, it does not appear that gestational age was included in the models used.

5. While the finding that Klebsiella species (particularly the LPS synthesizers) could result in higher calprotectin levels and poor seroconversion, this explanation should not be considered causal. It is very possible that other factors, for example, concurrent enteric infections or antibiotic exposure during delivery (which was not captured), could have resulted in microbiome perturbations that led to poor RVV response, with the increase in Klebsiella and calprotectin associated but not causative.

Minor point: For Table 1, please add the word "days"

(Remarks on code availability)

Reviewer #2

(Remarks to the Author)
In this manuscript Seymour et. al. conduct a large analysis of infant microbiomes from three countries to identify connections between the infant microbiome and oral rotavirus vaccine response. The authors find that the bacterial genus Klebsiella is anti-associated with vaccine response, and conduct a number of additional analyses to make conclusions about why this association exists. I found the dataset to be a major strength of the study, and the statistical approaches applied are sophisticated and generally appropriate for addressing numerous potential confounders. However, several results were difficult to interpret within the statistical framework presented, key methodological details regarding sequencing were omitted, and additional discussion clarifying how this work advances beyond prior studies would strengthen the manuscript (see detailed comments below). Overall I believe this work is an important contribution to the scientific field and that it could be appropriate for publication in Nature Communications pending revisions.

Sincerely,
Matt Olm
CU Boulder

1) The metagenomics methods section lacks several key details, most notably the sequencing depth per sample. I was unable to locate this information in the manuscript or supplementary materials. Sequencing depth is critical for evaluating limits of detection and interpreting downstream analyses.

2) On line 536, the authors state: “Data were rarefied to 7,578 reads per sample, resulting in 793 samples for analysis.” This is an unusually low read count for metagenomic analyses. Why was such an aggressive rarefaction threshold necessary?

3) The metagenomic workflow appears more complex than necessary. Specifically, reads were mapped to genomes in the “Web of Life reference version 2” to generate OGUs, which were later converted to GTDB references. A more direct approach would be to map reads directly to GTDB reference genomes (e.g., using tools such as sylph), producing an OGU matrix, phylogenetic tree, and taxonomy in a single framework. While this is not inherently problematic, it would be helpful for the authors to explain the rationale for this pipeline choice.

4) The introduction and discussion appropriately reference related work, but it remains unclear which findings in this study are novel versus confirmatory. For example, the manuscript states that microbial configurations influencing RVV efficacy remain poorly defined and that few studies have integrated longitudinal multi-modal data across populations. A table explicitly comparing prior studies with the current work, highlighting novel contributions, confirmatory findings, and newly integrated data types (microbial, inflammatory, immunological), would be helpful in clarifying this study’s contribution.

5) I found the section describing the cross-sectional gut community types (cs-GCTs) very difficult to follow on several levels. For example, the text in the paragraph starting on line 207 is very difficult to parse, and I wonder if a table or figure might be a clearer way of explaining this information. Second, I was not able to understand sentences like “Accounting for covariates, seroconversion status was associated with overall differences in M1 cs-GCT odds” and “Seroconverters displayed the highest odds associated with Klebsiella- and Escherichia-depleted M1 cs-GCT6 relative to cs-GCT3.” It is not clear what outcome the odds refer to, and more explicit descriptions and plain-language interpretations would improve accessibility. A summary table or schematic might also help.

6) Related to the above point, I would love to see some additional basic plots related to the cs-GCT data that’s easier to interpret than log odds-ratio. I understand that much of this work is wrapped up in complex statistical models, but I think it would still be useful for general understanding of this work to additional descriptive plots showing data like the following: Percentage of infants who seroconverted within each cs-GCT; Country distribution across cs-GCTs; Study-wide ordination (e.g., PCA/PCoA) colored by cs-GCT; Average abundance of key taxa within each cs-GCT. These visualizations might help readers build intuition about the patterns underlying the statistical findings.

7) There are too many colors in Figure 4A to parse by eye, and as a result, I was unable to tell what the dominant microbes are in each cs-GCT. Consider limiting the palette to a smaller number of key taxa (e.g., ~8) and grouping the remainder into an “Other” category.

8) I strongly commend the authors for including the section “Classification models of RV1 response do not generalize.” Reporting negative findings is valuable for the field and strengthens the manuscript.

9) I didn’t see any funding sources in the acknowledgments section, and the BioProject (PRJNA1404625) is not live. This is just a flag to make sure these are addressed before publication.

(Remarks on code availability)
Code looks approprite

Reviewer #3

(Remarks to the Author)
The manuscript presents a clear and convincing case of microbiota association with rotavirus vaccine response in multiple

countries. The results align with previous results from other countries, which supports the overall conclusions.

Major comments:

I would like to see a scatter plot of the association between Klebsiella abundance and IgA fold change as well as a boxplot of Klebsiella with seroconversion and virus shedding. That is needed for the reader to be able to judge how strong the associations are.

Was there an association between pre-vaccine IgA and Klebsiella abundance? How much of the seroconversion and virus shedding was explained by pre-vaccine IgA presence? The pre-vaccine immunity should be carefully considered as a potential causal contributor to the observed patterns. This would help develop some understanding of the causal patterns. The manuscript would benefit from conceptualising a model of how pre-existing immunity, Klebsiella abundance, infant background factors, seroconversion and virus shedding are related and then testing this as a path model.

Minor:
I would like to note that all the genera in Fig. 3A are LPS-producing enterobacteria, so Klebsiella may not be the only culprit, but maybe the overall LPS load is important.

Fig. 3C: could you spell out what the pathways are?

Fig. 5B: make sure the association holds after the most extreme values are removed. So re-run the statistics after removing the values with the lowest and highest Bif levels.

Line 98-101: "Pre-vaccine RV-IgA levels were higher in Peruvian compared to Panamanian and U.S. infants (P=0.06 and P<0.001 respectively), and Panamanian infants displayed higher pre-vaccine IgA than U.S. infants (P=0.064) (Figure 1C). " If the difference is not statisticaly significant, you cannot conclude that there is a difference. Correct to "Pre-vaccine RV-IgA levels were higher in Peruvian compared to U.S. infants (P<0.001), but similar between Panamanian and both Peruvian and U.S. infants (P=0.064 and P=0.064, respectively) (Figure 1C). "

(Remarks on code availability)
The code is clear and usable.

Version 1:

Reviewer comments:

Reviewer #1

(Remarks to the Author)
My comments were adequately addressed.

(Remarks on code availability)

Reviewer #2

(Remarks to the Author)
Authors have adequately addressed all of my concerns and I have no further suggestions for improvement.

(Remarks on code availability)

**Open Access** This Peer Review File is licensed under a Creative Commons Attribution 4.0 International License, which permits use, sharing, adaptation, distribution and reproduction in any medium or format, as long as you give appropriate credit to the original author(s) and the source, provide a link to the Creative Commons license, and indicate if changes were made.
In cases where reviewers are anonymous, credit should be given to 'Anonymous Referee' and the source.
The images or other third party material in this Peer Review File are included in the article's Creative Commons license, unless indicated otherwise in a credit line to the material. If material is not included in the article's Creative Commons license and your intended use is not permitted by statutory regulation or exceeds the permitted use, you will need to obtain permission directly from the copyright holder.
To view a copy of this license, visit https://creativecommons.org/licenses/by/4.0/

***Klebsiella*-dominated gut communities are associated with impeded oral rotavirus vaccine response in infants**

Author responses.

April 9, 2026

**REVIEWER COMMENTS**

Reviewer #1 (Remarks to the Author):

**This is an important work that integrates microbial and inflammatory measures with rotavirus vaccine (RVV) response data from infants across 3 diverse countries. Previous studies have were from single sites and focused on either microbiome community structure or biomarkers of environmental enteropathy, but not in the same study. Also the inclusion of machine learning methods to understand the complex data is novel. The noteworthy results are that Klebsiella species, particularly those with LPS biosynthetic genes were more abundant in infants that did not seroconvert. Higher fecal calprotectin concentrations was also associated with failure to seroconvert. In general the paper is well-written and there is enough detail provided for the work to be reproduced.**

Thank you for your detailed review.

**There are a few concerns or perhaps clarifications needed in the methods used:**

**1. Throughout the paper the authors measure a stool collect at 1 month of age, however, the methods state that stool samples were collected at 1-2 weeks and 2 months, but not 1 month. Which stool was used for the reported 1 month outcomes? Further, from Figure 1A, it appears that RVV shedding was assessed on the day of vaccination. Typically, one would assess this in the days/weeks after vaccination, once the vaccine virus has had a chance to replicate in the intestinal mucosa.**

We appreciate the reviewer's attention to detail. Stool was collected during study visits that were scheduled around the time of infant well-child check-ups at 1-2 weeks, 2, 4, and 6 months. While drafting the manuscript we debated calling the first timepoint 1-2 weeks, but this was confusing in the figure. Thus, we decided to label the visit M1 because it occurred during month 1 (median: 9 days, IQR: 4 days). We added the median information to the Results section on page 5, line 116, which had been included for the other visit timepoints. We also clarified the timepoints labeling in the methods section on page 13, lines 442 and 455.

RVV shedding was assessed only in infants with stool collected 1 to 14 days *after* vaccination (n-216). Shedding is seen as soon as 1 day post-vaccination based on PMID: 24342877. The timing was stated in the first paragraph of the results and now clarified further in the 3rd paragraph of the results on page 4. Figure 1 provides a general representation of all specimens collected at the 4 month time point which included 216 collected in the 1-14 days after vaccination and 30 collected on the day of vaccination. All stools were used in the metagenome analysis, but only the 216 collected after vaccination were used to assess shedding.

**2. There is no mention of maternal antibody levels and their association with the seroconversion outcome. Several studies have shown that transplacentally-acquired antibody and human milk antibodies can block the vaccine virus replication, and decrease immunogenicity. If there are no maternal serum samples available for this analysis, this should be included as a limitation.**

Thank you for this question. We assessed maternal human milk antibodies in our cohort and found no association between milk RV-specific IgA or neutralization activity and infant seroconversion. A separate manuscript describing these findings is under review. The findings are consistent with epidemiologic studies showing that withholding breastfeeding before/after vaccination does not affect immune response. We did not evaluate these data because we felt that doing so would detract from the metagenome analysis. We do not expect that human milk RV-specific IgA is related to the composition of the infant gut microbiome (or vice versa).

We did not assess maternal RV-IgG. We added the following to the limitation paragraph on page 12, line 413: “Transplacentally-acquired maternal IgG can decrease RVV immunogenicity; we did not assess maternal RV-specific antibody levels.”

**3. Diet is a complicated construct. The methods do not describe what what collected for the diet variable. It appears that it was breastfed/mixed/formula-fed in one area and breastfed/formula-fed in one of the results figures. Was this data collected at each visit? Instead of diet, that may be better described as breastfeeding status. Related to point 2 above, breastfeeding would not only influence microbiome composition, but would also influence seroconversion, if the maternal milk contains either adaptive or non-adaptive immune factors that block vaccine take. How was this considered in the models used?**

At each visit, we collected comprehensive dietary information including the breastfeeding status, frequency, duration, formula intake and types of solid foods. We

have added additional details to the methods section, “Study design and participants” (Page 13, line 440).

The infant diet should consist only of human milk or formula during the first 6 months of life per the WHO recommendations. In our cohort, none of the infants had been introduced to solid foods at 2 months of age (RV dose 1). However, 39 out of 229 had been introduced by 4 months (RV dose 2). This information was added to the legend of figure 2.

We have further studies planned to evaluate the milk microbiome, human milk oligosaccharides, and additional adaptive and non-adaptive immune factors. We felt that these were beyond the scope of this manuscript. We added human milk assessment as a consideration in the limitations paragraph (page 12, line 414). However, we are thankful for your excitement about this question.

**4.In Figure 2B, country, gestational age at birth, child age, and receipt of breastmilk/formula were the factors that resulted in the greatest differences in diversity. However, it does not appear that gestational age was included in the models used.**

We thank the reviewer for flagging this issue. We had omitted gestational age from the M1 cross-sectional differential abundance and gut type analyses. It has now been included.

With the re-analysis, the results show minor changes: 1) the species-level multivariable differential abundance analysis p-value associated with *K. grimontii* is now 0.057 and is no longer statistically significant.

There are no changes in interpretation.

We have updated the main text on line 163 (Page 6), line 234 (Page 7), and associated figures to reflect this change.

**5. While the finding that Klebsiella species (particularly the LPS synthesizers) could result in higher calprotectin levels and poor seroconversion, this explanation should not be considered causal. It is very possible that other factors, for example, concurrent enteric infections or antibiotic exposure during delivery (which was not captured), could have resulted in microbiome perturbations that led to poor RVV response, with the increase in Klebsiella and calprotectin associated but not causative.**

We agree with the reviewer. Our use of ‘association’ in the title of the paper was intentional; our experimental framework does not describe a causal relation, only one of

association. We added as a limitation in our discussion: “This study was observational; our framework is unable to assess causal relation between RV1 response and the infant gut microbiome.” (Page 12, line 415)

**6. Minor point: For Table 1, please add the word "days"**

Added. Thank you.

Reviewer #2 (Remarks to the Author):

**In this manuscript Seymour et. al. conduct a large analysis of infant microbiomes from three countries to identify connections between the infant microbiome and oral rotavirus vaccine response. The authors find that the bacterial genus Klebsiella is anti-associated with vaccine response, and conduct a number of additional analyses to make conclusions about why this association exists. I found the dataset to be a major strength of the study, and the statistical approaches applied are sophisticated and generally appropriate for addressing numerous potential confounders. However, several results were difficult to interpret within the statistical framework presented, key methodological details regarding sequencing were omitted, and additional discussion clarifying how this work advances beyond prior studies would strengthen the manuscript (see detailed comments below). Overall I believe this work is an important contribution to the scientific field and that it could be appropriate for publication in Nature Communications pending revisions.**

Thank you for your review and suggestions.

**1) The metagenomics methods section lacks several key details, most notably the sequencing depth per sample. I was unable to locate this information in the manuscript or supplementary materials. Sequencing depth is critical for evaluating limits of detection and interpreting downstream analyses.**

We thank the reviewer for this important observation. We have added sequencing depth information to the methods section, page 15, line 505-512.

“OGUs with <1% coverage were removed, yielding a dataset of 9,556,896,551 reads across 928 samples and 1,698 OGUs, with a median sequencing depth of 7,016,230 (IQR: 8,469,467) reads per sample. Samples with fewer than 500,000 reads were subsequently removed, which also excluded all negative controls and blanks, resulting in 835 samples. Positive controls were further removed, yielding a final dataset of 792 fecal samples comprising 9,016,814,196 reads and 1,623 OGUs, with a median sequencing depth of 7,520,388 (IQR: 8,216,599) reads per sample.”

**2) On line 536, the authors state: "Data were rarefied to 7,578 reads per sample, resulting in 793 samples for analysis." This is an unusually low read count for metagenomic analyses. Why was such an aggressive rarefaction threshold necessary?**

We thank the reviewer for this important comment. We would like to clarify that the reported rarefaction depth of 7,578 refers to **pathway-level counts**, not raw sequencing reads. Taxonomic profiles were rarefied at 500,000 reads per sample. In contrast, functional profiles were generated by mapping reads to MetaCyc pathways, resulting in substantially lower and more variable total counts per sample due to aggregation of reads into functional features. The rarefaction threshold of 7,578 was selected to **maximize sample retention** while standardizing sampling effort across samples, rather than reflecting sequencing depth. To ensure that our findings were not driven by this choice, we performed a sensitivity analysis in which Shannon diversity was calculated using non-rarefied pathway abundances normalized to relative abundance. Results were highly consistent between approaches (Spearman $\rho = 0.983$, $p < 2.2 \times 10^{-16}$), indicating that the observed patterns are robust to normalization strategy.

We have clarified our methods on page 16, lines 551-561.

**3) The metagenomic workflow appears more complex than necessary. Specifically, reads were mapped to genomes in the "Web of Life reference version 2" to generate OGUs, which were later converted to GTDB references. A more direct approach would be to map reads directly to GTDB reference genomes (e.g., using tools such as sylph), producing an OGU matrix, phylogenetic tree, and taxonomy in a single framework. While this is not inherently problematic, it would be helpful for the authors to explain the rationale for this pipeline choice.**

We thank the reviewer for their attention to detail. The rationale for our workflow was to leverage reference-based definitions via the Web of Life to conduct the analysis. Analyzing function was a priority, and the Web of Life database offers metagenomics based on uniform closed-reference functional annotation, taxonomy, and OGU definition. This differentiates the database from comparable technologies, namely Kraken, Metaphlan, or sylph, by which functional annotation must be performed separate from taxonomic annotation.

Regarding GTDB specifically, the Web of Life uses GTDB r202 for higher-order taxonomy. No additional conversion was required; this is the standard ontology provided as part of the database export. However, as written the reference to GTDB may be

confusing to the reader. To clarify that we use the GTDB taxonomy provided by the WoL database, we have modified the methods on page 15, lines 516-518: “Reference taxonomy reflecting Genome Taxonomy Database (GTDB) 202 designations for each OGU were obtained from the Web of Life release 2.”

**4) The introduction and discussion appropriately reference related work, but it remains unclear which findings in this study are novel versus confirmatory. For example, the manuscript states that microbial configurations influencing RVV efficacy remain poorly defined and that few studies have integrated longitudinal multi-modal data across populations. A table explicitly comparing prior studies with the current work, highlighting novel contributions, confirmatory findings, and newly integrated data types (microbial, inflammatory, immunological), would be helpful in clarifying this study’s contribution**.

Overall, the current literature surrounding rotavirus vaccine responses and the gut microbiome suffers from the following limitations: Studies are mostly single cohort, rely on limited sequencing (principally 16S), and lack longitudinal sampling. We have included for the reviewer a table that describes previous studies that explore the relationship between the infant gut microbiome and oral rotavirus vaccines below.

While prior studies presented important findings, we believe our presented study is an important <u>further</u> step in deciphering the complex associations between the gut microbiome and vaccine response.

- Our cohort includes infants from 3 nations of varying socioeconomic level. The models used integrate country level variables allowing for cross country comparisons within the same statistical framework.
- DMM modelling across multiple timepoints allowed us to demonstrate, with granularity, the evolution of the microbiome over time – parsing that effect away from the rotavirus vaccine response.
- We included both seroconversion and shedding as measures of vaccine response. We also included a clinically relevant metric of inflammation in the form of the fecal calprotectin assay.
- Our statistical modelling is more suitable for a complex ecological cohort.

| Manuscript | Cohort | Major findings | Comparisons to presented work |
|---|---|---|---|
| *Miranda et al., preprint (2025)* https://doi.org/10.1101/2025.09.22.25336338 | **Single cohort study**. **RotaTeq and Rotarix. 16S sequencing** of **US infants.** Compared the month 1 microbiome to month 6 RV IgA titer. Primarily modelled alpha diversity vs RV titer. | Higher alpha diversity at M1 was associated with higher RV IgA titer at month 6.<br><br>*Collinsella*, *Atopobium* and *Schaalia* are positively associated with RV titer. *Bifidobacterium*, *Lactobacillus*, *Klebsiella*, *Escherichia-shigella* and *Streptococcus* were all negatively associated. | Only measured 16S in a single country cohort. Alpha diversity vs RV IgA is a relatively simplistic model likely limiting the findings.<br><br>Our study uses **metagenomics** in a **3-country cohort**. The use of DMM modelling allows us to parse our data more discreetly leading to more complex and statistically valid modelling.<br><br>The basic analyses within our framework **confirms the finding relevant to higher diversity**. Our **species level findings provide more granularity** – coupled with **inflammatory readouts** that lend greater clinical relevance. |
| *Wagner et al., Nature Communications (2025)* https://doi.org/10.1038/s41467-025-58632-6 | **RV3-BB trial patients** from an **Indonesian and Malawian cohort. Neonatal vaccination schedule 16S sequencing.** 193 children. | Higher alpha diversity, beta diversity and relative abundance of *Bacteroides* is associated with shedding but not seroconversion in the neonatal schedule. | **Only measured 16S.**<br><br>The findings of increased *Streptococcus* and *Staphylococcus* in participants with no shedding aligns with the theory of increased inflammatory bacteria |

| | | | |
|---|---|---|---|
| | Vaccine response assessed via **RV IgA seroconversion and/or stool shedding**. | *Streptococcus* and *Staphylococcus* are associated with no shedding. | being detrimental to vaccine uptake. Limitations: The study has no inflammatory measure and shedding alone is a suboptimal measure of vaccine response.<br><br>**Our work** provides **increased granularity of metagenomics** and **clinically relevant inflammatory outputs**. |
| *Ngo et al., Cellular and Molecular Gastroenterology and Hepatology (2024)* https://doi.org/10.1016/j.jcmgh.2024.101393 | Mice receiving FMTs from high or low RV vaccine responding human infants. | Suggests involvement of *Clostridium* species in RV vaccine non-response | This work was mechanistically focused. Though FMTs were derived from human infants, the relevance of mouse physiology and vaccine response to humans must be considered. |
| *Cunningham-Oakes et al., BMC Microbiology (2023)* https://doi.org/10.1186/s12866-023-03098-z | **16S and Metagenomic sequencing** of Rotarix vaccinated infants from **India and Malawi**. 16S sequencing was performed longitudinally at 1, 4, 6 and 10 weeks of like whilst metagenomics was only performed at week 6, at the | Higher genus and species level alpha diversity in vaccine non-responders.<br><br>Modelling exhibited poor predictive accuracy. | 16S sequencing formed the bulk of the dataset. Metagenomics was only performed at 1 timepoint.<br><br>Our modelling also shows limited generalizability but was able to parse out microbiome and functional responses. **Our metagenomic data was also longitudinal rather than single timepoint.** |

| | | | |
|---|---|---|---|
| | time of first Rotarix dose | | The major added **benefits of our work** are the **more robust modelling, DMM framework** and inclusion of **inflammatory outputs.** |
| *Robertson et al., Vaccine (2021)* https://doi.org/10.1016/j.vaccine.2021.07.076 | **Single country cohort** of 158 **Zimbabwean** infants. **Shotgun metagenome sequencing** analyzed against serum anti rotavirus IgA following Rotarix vaccination. | No significant differences in diversity of composition between vaccine seroconverters and non-converters. | Stool was only collected at one pre-vaccine and one post-vaccine timepoint.<br><br>Analyses were limited to random forest models and basic regressions.<br><br>A single (low responding) country cohort likely limits the ability of basic models to detect anything significant.<br><br>**Our study** built a **more ecologically valid and complex model** integrating vaccine and **inflammatory outputs**. |
| *Fix et al., The American Journal of Tropical Medicine and Hygiene (2019)* https://doi.org/10.4269/ajtmh.19-0355 | **Single country cohort** of 50 **Nicaraguan** infants vaccinated with RV5 (RotaTeq).<br><br>16S sequencing of the microbiome pre vaccine compared between vaccine | No significant differences in microbiome composition between responders and non-responders. | As with other single country cohorts the ability to parse out significant association between vaccine efficacy and the microbiome are limited.<br><br>**Our study benefits from a wider multi-country cohort** |

| | | | |
|---|---|---|---|
| | responders or non-responders (seroconversion). | | allowing for more **complex and ecologically valid analyses**. |
| *Harris et al., Cell Host & Microbe (2018)*<br><br>https://doi.org/10.1016/j.chom.2018.07.005 | Cohort of **healthy adults aged 18-35** recruited from a high-income country. Three analysis groups including treatment with broad spectrum antibiotics, narrow spectrum and no antibiotics.<br><br>Measured the effect of pre-vaccine antibiotics on RV vaccine shedding and IgA in response to Rotarix vaccination. **Included 16S microbiome analysis** | Antibiotics increased immunogenicity though only at very early timepoints proximal to the dose. Increased abundance of Proteobacteria, especially those of the *Escherichia-shigella* genus correlate with RV vaccine shedding but not serology. | A single country cohort of healthy adults is not generalizable to pediatric responses in either high- or low-income countries. |
| *Parker et al., Vaccine (2017)* https://doi.org/10.1016/j.vaccine.2017.11.031 | **Single country cohort** of **South Indian** infants vaccinated with oral **Rotarix**.<br><br>16S sequencing and Taqman array real-time PCR on pre-vaccine stool compared against shedding and seroconversion. | No consistent or significant association between the bacterial microbiota and serological response or shedding. | As with other single country cohorts, the ability to parse out significant association between vaccine efficacy and the microbiome are limited.<br><br>**Our study benefits from a wider multi-country cohort** allowing for more **complex and** |

| | | | |
|---|---|---|---|
| | | | **ecologically valid analyses**. |
| *Harris et al., Gut Microbes (2017)* https://doi.org/10.1080/19490976.2017.137616 2 | Nested, case-control study. Compared the pre-vaccine microbiota (microarray) of 10 6-week-old **Pakastani infants** who responded to Rotarix vs 10 who did not (as determined by IgA seropositivity. Also included **10 healthy Dutch infants as a control**. | RV1 response was correlated with higher abundance of *Clostridia* and Proteobacteria (*Serratia* and *Escherichia*). | **16S sequencing.**<br><br>**Assayed principally phylogenetic differences** in a limited (though well matched) cohort. |
| *Harris et al., The Journal of Infectious Diseases (2016)* https://doi.org/10.1093/infdis/jiw518 | Nested, case-control study. Compared the pre-vaccine microbiota (microarray) of **39 vaccine responder/non-responder pairs from rural Ghana** to Dutch infants. | Ghanaian responders were more similar to Dutch infants than nonresponders.<br><br>Ghanian infants with a high abundance of the Bacteroidetes phylum were correlated with poor vaccine response. | The modelling in both the *Harris et al* papers are limited, by both the type of assay used, cohort and statistical method. It is possible that both studies are simply detecting the microbiome signatures of each country. The lack of longitudinal timepoints prevents meaningful analyses with respect to time.<br><br>The major added **benefits of our work** are the **more robust modelling, DMM framework** and inclusion of **inflammatory outputs** |

**5) I found the section describing the cross-sectional gut community types (cs-GCTs) very difficult to follow on several levels. For example, the text in the paragraph starting on line 207 is very difficult to parse, and I wonder if a table or figure might be a clearer way of explaining this information. Second, I was not able to understand sentences like "Accounting for covariates, seroconversion status was associated with overall differences in M1 cs-GCT odds" and "Seroconverters displayed the highest odds associated with Klebsiella- and Escherichia-depleted M1 cs-GCT6 relative to cs-GCT3." It is not clear what outcome the odds refer to, and more explicit descriptions and plain-language interpretations would improve accessibility. A summary table or schematic might also help.**

We thank the reviewer for their critique of the verbiage in this section. Our approach is conceptually similar to the statistical approach implemented by Maaslin2. Instead of using linear regression to quantify associations between covariates and species abundances, we use a multinomial logistic regression to identify covariate associations to 'risk ratios' of cross-sectionally generated Dirichlet-Multinomial clusters (DMMs or cs-GCTs).

We agree that this section is difficult to follow and have made the following modifications to address the critiques:

- We have modified the introductory paragraph on page 7, starting at line 208 as follows:

  > "To describe overall patterns in infant gut community composition, we applied Dirichlet's Multinomial Mixture modeling[24] and generated cross-sectional gut community types (cs-GCTs). These summarize as a series of categorical variables the configuration of the gut microbiome at M1, M2, and M4 (**Figure 4A**, **Extended Data Figure 4**). To highlight combinations of genera that define each gut type, we applied differential abundance analysis (DAA) comparing cs-GCTs (**Figure 4B**, **Extended Data Figure 5A,B**): Here, the gut type M1 cs-GCT3 is enriched in *Klebsiella*…"

- We clarify in the following paragraph that gut type designation is the outcome variable in the model, and include on page 7, lines 228-232:

  "Using cs-GCTs as the outcome, a multinomial model was used to quantify association of clinical variables and RV1 seroconversion to the odds or infants assigning to particular gut types at each timepoint. This model was selected to quantify the extent to which RV1 seroconversion associates with each gut type while

accounting for other factors related to beta diversity such as country of origin, diet and breastfeeding."

Finally, we added the following schematic as supplemental figure that describes the organization of the cs-GCT multinomial model:

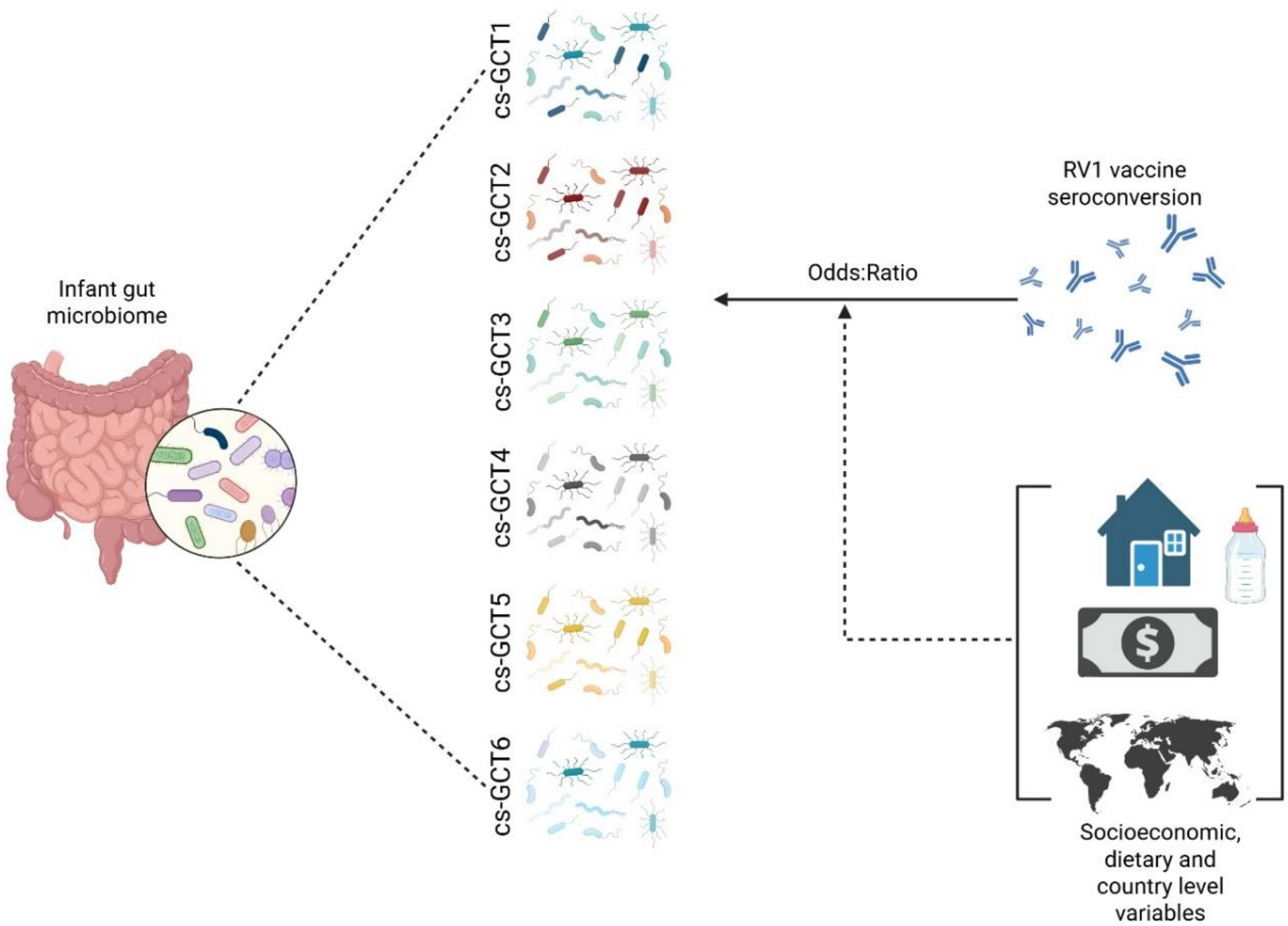


**Supplemental Figure 7. Cross-sectional gut community type (cs-GCT) analysis workflow diagram.** For this analysis, odds ratios computed reflect marginal associations (via type-III sum-of-squares ANOVA) between RV1 vaccine seroconversion and each cs-GCT, simultaneously considering contribution due to variation in gut types associated with demographic, dietary, and other variables that modify the infant gut microbiome. Specific model formulae are available in **Supplemental Text 2**.

**6) Related to the above point, I would love to see some additional basic plots related to the cs-GCT data that's easier to interpret than log odds-ratio. I understand that much of this work is wrapped up in complex statistical models, but I think it would still be useful for general understanding of this work to additional descriptive plots showing data like the following: Percentage of infants who seroconverted within each cs-GCT; Country distribution across cs-GCTs; Study-wide ordination (e.g., PCA/PCoA) colored by cs-GCT; Average abundance of key taxa within each cs-GCT. These visualizations might help readers build intuition about the patterns underlying the statistical findings.**

Thank you for this critique. We have made the following changes:

We have created a new Extended Data Figure 6, in which panel A includes the percentage of infants who seroconverted within each cs-GCT. Likewise, we include a table of per- cs-GCT response statistics via chi-squared test:

**A**

| | Seroconversion | | Seropositivity (M2) | | Shedding (M4) | | Seropositivity (M6) | |
|---|---|---|---|---|---|---|---|---|
| | NR | R (%) | - | + (%) | - | + (%) | - | + (%) |
| cs-GCT1 | 31 | 37 (54.4%) | 64 | 4 (5.9%) | 28 | 20 (41.7%) | 52 | 16 (23.5%) |
| cs-GCT2 | 25 | 25 (50.0%) | 41 | 9 (18.0%) | 24 | 12 (33.3%) | 32 | 18 (36.0%) |
| cs-GCT3 | 24 | 18 (42.9%) | 38 | 4 (4.0%) | 22 | 4 (15.4%) | 35 | 7 (16.7%) |
| cs-GCT4 | 20 | 19 (51.3%) | 31 | 8 (20.5%) | 15 | 12 (44.4%) | 18 | 21 (53.8%) |
| cs-GCT5 | 13 | 17 (56.7%) | 25 | 5 (16.7%) | 9 | 10 (52.6%) | 19 | 11 (36.7%) |
| cs-GCT6 | 6 | 17 (73.9%) | 22 | 1 (4.3%) | 11 | 5 (31.2%) | 13 | 10 (43.5%) |
| | P = 0.265 | | P = 0.121 | | P = 0.119 | | P = 0.004 * | |

We moved Panel C from Extended Data Figure 5 to Extended Data Figure 6B, that includes study-wide ordinations colored by M1 cs-GCTs:

**C**

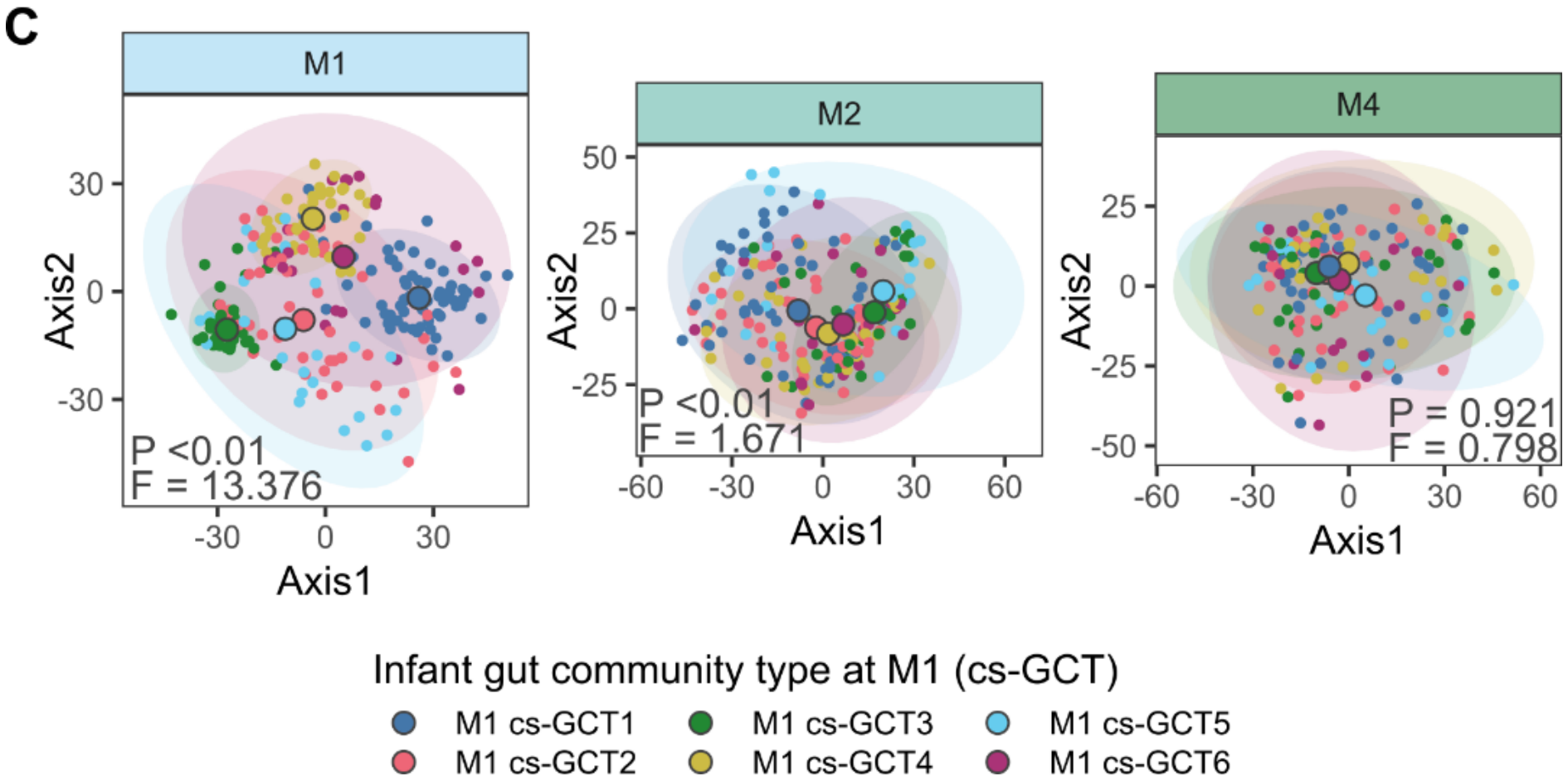


To briefly indicate the distribution of countries per cs-GCT, we have added indicators representing the number and percentage of infants per country assigned to each cs-GCT in Figure 3A:

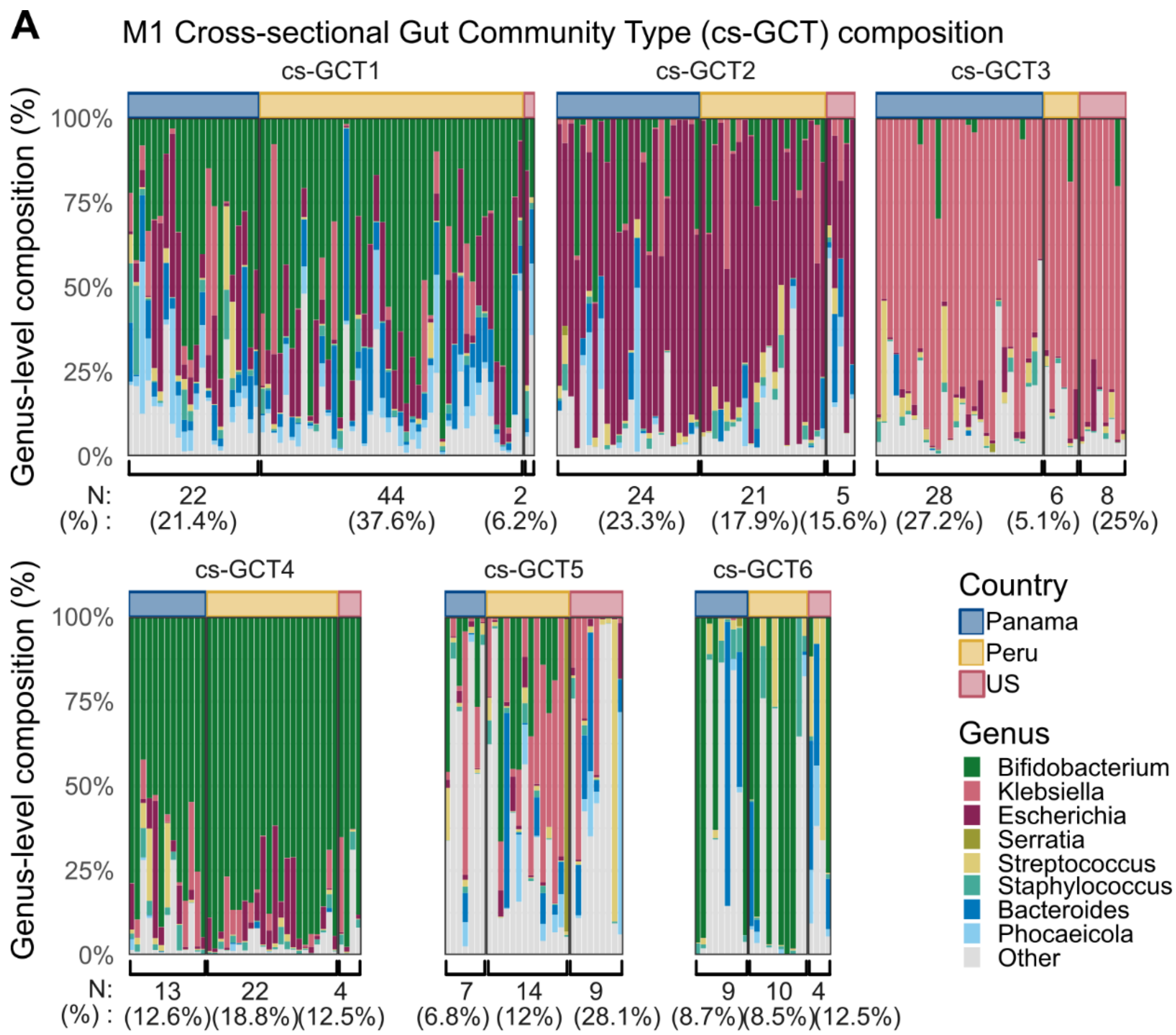
A
M1 Cross-sectional Gut Community Type (cs-GCT) composition
cs-GCT1
cs-GCT2
cs-GCT3
Genus-level composition (%)
100%
75%
50%
25%
0%
N:
(%) :
22
(21.4%)
44
(37.6%)
2
(6.2%)
24
(23.3%)
21
(17.9%)
5
(15.6%)
28
(27.2%)
6
(5.1%)
8
(25%)
cs-GCT4
cs-GCT5
cs-GCT6
13
(12.6%)
22
(18.8%)
4
(12.5%)
7
(6.8%)
14
(12%)
9
(28.1%)
9
(8.7%)
10
(8.5%)
4
(12.5%)
Country
Panama
Peru
US
Genus
Bifidobacterium
Klebsiella
Escherichia
Serratia
Streptococcus
Staphylococcus
Bacteroides
Phocaeicola
Other

Finally, we created a new supplemental figure 8 showing a breakdown of percent abundances for important taxa across all cs-GCTs at each timepoint:

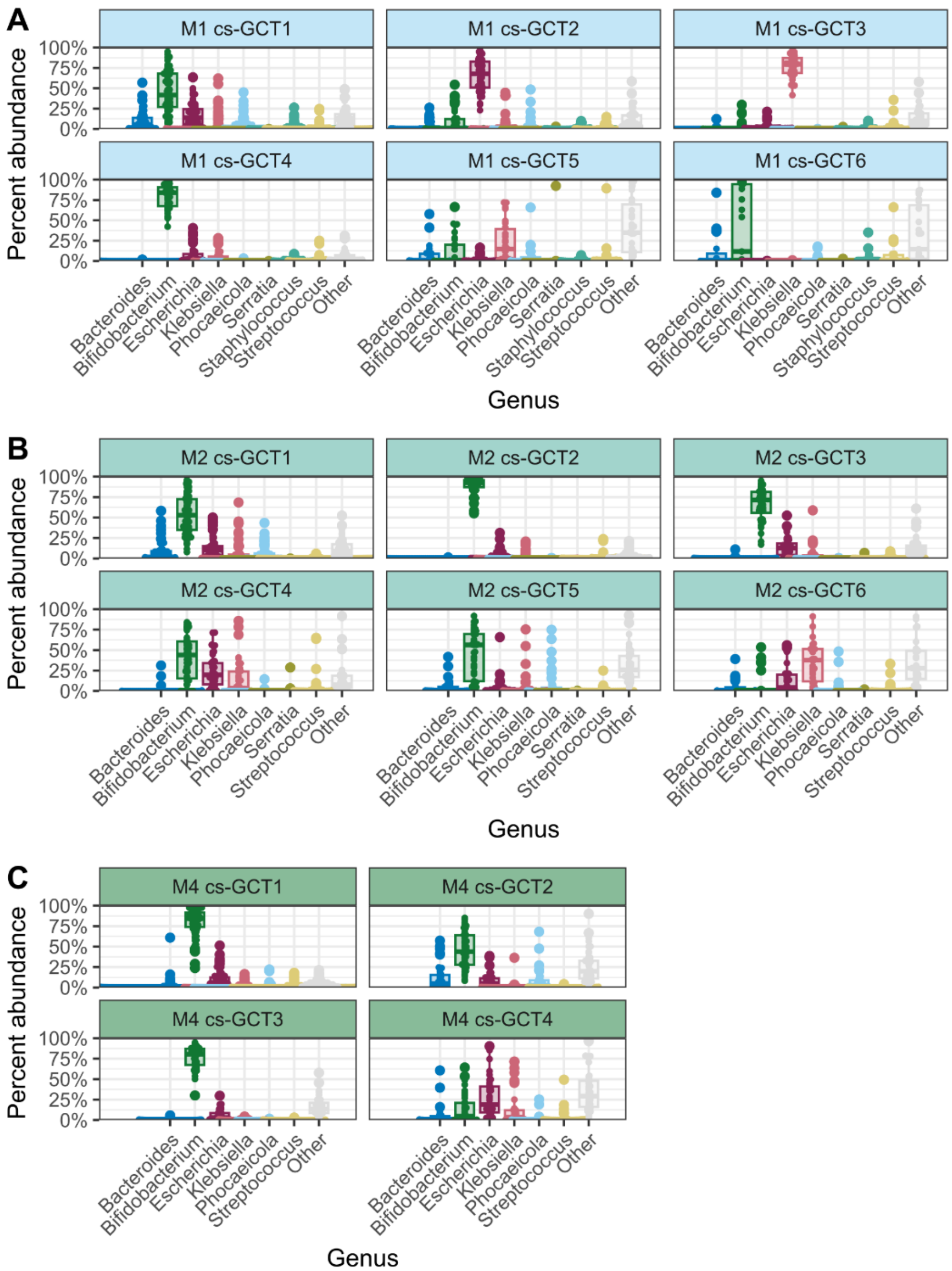


**Supplemental Figure 8. Abundance of select taxa within each gut type.** Boxplots showing percent abundances of major taxa in gut types at each timepoint: (**A**) M1, (**B**) M2, (**C**) M4.

**7) There are too many colors in Figure 4A to parse by eye, and as a result, I was unable to tell what the dominant microbes are in each cs-GCT. Consider limiting the palette to a smaller number of key taxa (e.g., ~8) and grouping the remainder into an "Other" category.**

We have taken the reviewer's recommendation and reduced the number of taxa displayed in the barplots in Fig 4A. We have reduced the number of taxa to 8 + an 'other' category (see figure in response above).

**8) I strongly commend the authors for including the section "Classification models of RV1 response do not generalize." Reporting negative findings is valuable for the field and strengthens the manuscript.**

Thank you for the reassurance of the value of this section.

**9) I didn't see any funding sources in the acknowledgments section, and the BioProject (PRJNA1404625) is not live. This is just a flag to make sure these are addressed before publication.**

Thank you for flagging these items. The funding acknowledgement has been added. The study was funded by NIH NIAID R01HD100542.

The reviewer link for the BioProject and associated SRA metadata are available at https://urldefense.com/v3/__https://dataview.ncbi.nlm.nih.gov/object/PRJNA1404625?reviewer=h1aos2qukrubsjtin4b1d1mt8r__;!!LLK065n_VXAQ!gzAGoquaHI9Vx9PNzZgPj_jk6ePlQDw_GgbrRY0vuwL3_pHeIwcX9ggq9f5cYGh5-BA8gN4Ts6DShHXDaWyf$ in read-only format. This will be made live upon publication.

**Reviewer #2 (Remarks on code availability):**

**Code looks appropriate**

-------

Reviewer #3 (Remarks to the Author):

**The manuscript presents a clear and convincing case of microbiota association with rotavirus vaccine response in multiple countries. The results align with previous results from other countries, which supports the overall conclusions.**

Thank you for your review and suggestions.

Major comments:

**1. I would like to see a scatter plot of the association between Klebsiella abundance and IgA fold change as well as a boxplot of Klebsiella with seroconversion and virus shedding. That is needed for the reader to be able to judge how strong the associations are.**

Thank you for this suggestion. We have added a new Extended Data Figure 7. Panel A shows no relationship between *Klebsiella* abundance and pre-vaccination anti-RV IgA. Panel B shows the significant relationship between IgA foldchange and *Klebsiella* abundance. Likewise, Panels C and D are violin plots with seroconversion and shedding to help visualize data that do not follow a strict gaussian distribution:

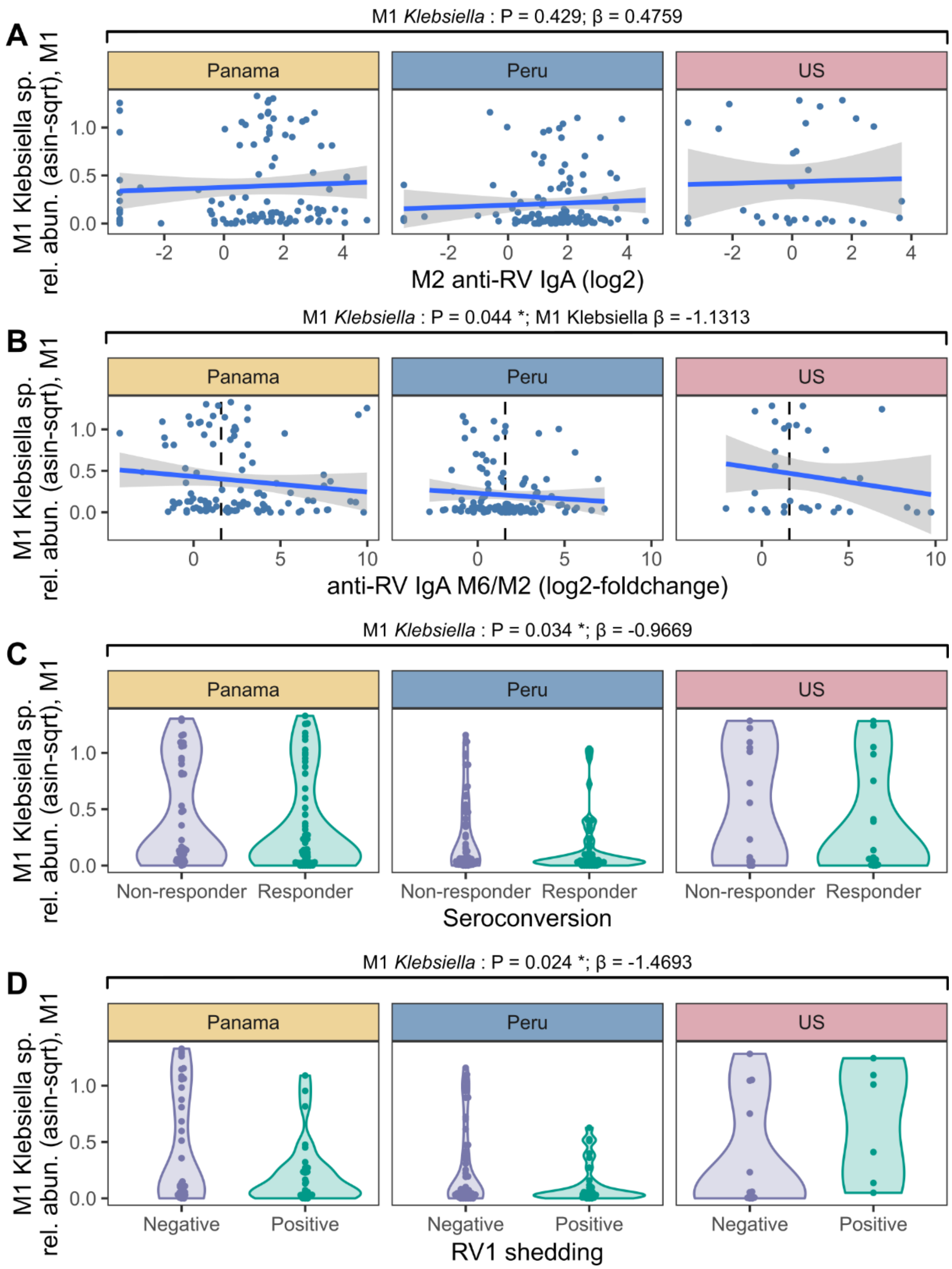


**Extended Data Figure 7. Additional analysis of *Klebsiella* relative abundance and pre- and post-RV vaccine findings:** (**A**) anti-RV IgA pre-vaccine. (**B**) anti-RV foldchange M6/M2, with dotted lines drawn at 3-fold IgA difference M6 / M2, indicating seroconversion. (**C**) RV1 seroconversion, (**D**) and RV1 shedding detection at M4. Statistics indicated above each panel were calculated via linear (pre-vaccine IgA and IgA foldchanges) or logistic (for seroconversion and shedding) regression + Wald test including country and untransformed M1 *Klebsiella* sum relative abundance as terms.

**2. Was there an association between pre-vaccine IgA and Klebsiella abundance? How much of the seroconversion and virus shedding was explained by pre-vaccine IgA presence? The pre-vaccine immunity should be carefully considered as a potential causal contributor to the observed patterns. This would help develop some understanding of the causal patterns. The manuscript would benefit from conceptualising a model of how pre-existing immunity, Klebsiella abundance, infant background factors, seroconversion and virus shedding are related and then testing this as a path model.**

As our experimental framework is observational, we are unable to describe a causal relationship (only correlations).

To clarify the question of pre-vaccine immunity, we included an additional panel in Extended Data Figure 7. The figure demonstrates that *Klebsiella* abundance is inverse to IgA foldchange as well as seroconversion (see figure above), however is not correlated to pre-vaccine IgA. This agrees with our beta diversity and cs-GCT analyses, in which we account for pre-vaccine IgA using model terms and note the same pattern.

---

Regarding a path model: we were unable to identify a structural equation modeling (SEM) framework that natively enables integration of the data models that we use to describe this cohort.

SEM frameworks are limited in their ability to model the relationship of covariates to microbiome data. Specifically: popular SEM frameworks (R's lavaan or piecewiseSEM) do not support endogenous categorical variables like cs-GCTs or cumulative longitudinally-measured covariates i.e: breastfeeding modality.

**Minor:**

**3. I would like to note that all the genera in Fig. 3A are LPS-producing enterobacteria, so Klebsiella may not be the only culprit, but maybe the overall LPS load is important.**

Thank you for this observation. We agree that all genera shown in Fig. 3A are LPS-producing enterobacteria, and that Panel A alone cannot distinguish a *Klebsiella*-specific effect from a broader gram-negative LPS load. This is precisely why we extended the analysis in 3D, where differential abundance of KEGG orthologs associated with LPS biosynthesis stratified by species demonstrated that specifically *Klebsiella* species

carrying LPS biosynthetic genes are enriched among non-responders. This analysis links taxonomic signal to functional potential and supports our interpretation.

To improve clarity, we have slightly revised the figure legend in 3A: "… Percentages indicate contribution to total variation across dbRDA1. *Klebsiella* was the dominant contributor at 8.6%."

**4. Fig. 3C: could you spell out what the pathways are?**

We thank the reviewer for this suggestion. The pathways shown in Fig. 3C are: the superpathway of lipopolysaccharide biosynthesis (LPSSYN-PWY), superpathway of (Kdo)2-lipid A biosynthesis (KDO-NAGLIPASYN-PWY), Kdo transfer to lipid IVA III (PWY-6467), O-antigen building blocks biosynthesis in *E. coli* (OANTIGEN-PWY), and three superpathways of O-antigen building blocks biosynthesis derived from GDP-mannose (PWY-7323), UDP-glucose (PWY-7328), and UDP-N-acetylglucosamine (PWY-7332).

These pathways collectively represent the major biosynthetic routes for LPS core and O-antigen structures. We have updated the figure legend accordingly:

> "Pathways shown include the superpathway of LPS biosynthesis (LPSSYN-PWY), (Kdo)2-lipid A biosynthesis (KDO-NAGLIPASYN-PWY), Kdo transfer to lipid IVA III (PWY-6467), O-antigen building blocks biosynthesis (OANTIGEN-PWY; PWY-7323; PWY-7328; PWY-7332)."

**5. Fig. 5B: make sure the association holds after the most extreme values are removed. So re-run the statistics after removing the values with the lowest and highest Bif levels.**

Thank you for this comment. We share the reviewer's dedication to statistical rigor. To account for low Bif levels, below, we include for the reviewer the same analysis using Tweedie regression, which is used to address to zero-inflation and is recommended by the authors of Maaslin2 if the user suspects zero-abundance samples may interfere with the regression:

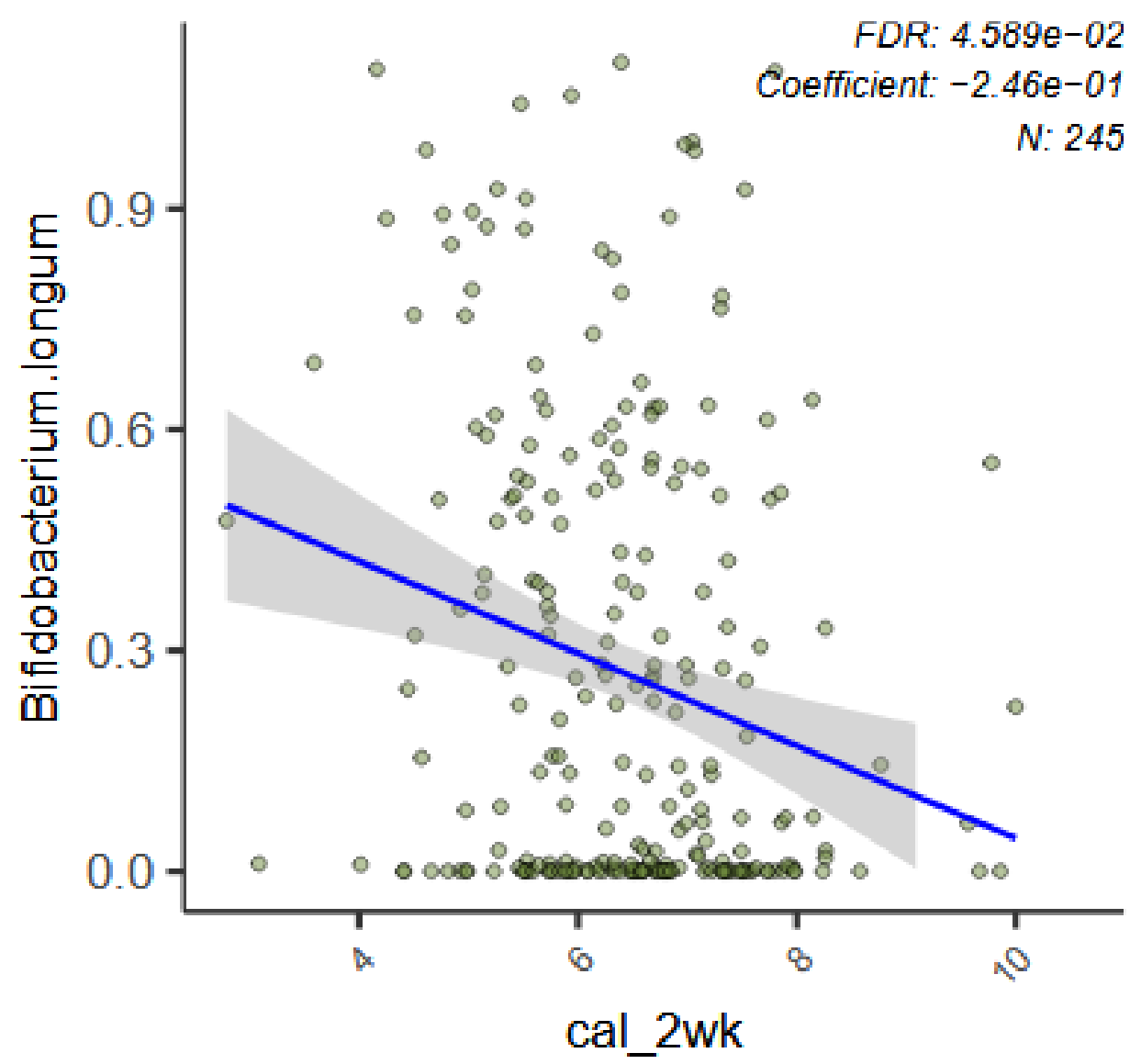


The interpretation regarding *B. longum* is robust to adjustment in model specification;the taxon remains negatively correlated with calprotectin at M1, with an FDR-corrected p-value of 0.04589.

**6. Line 98-101: "Pre-vaccine RV-IgA levels were higher in Peruvian compared to Panamanian and U.S. infants (P=0.06 and P<0.001 respectively), and Panamanian infants displayed higher pre-vaccine IgA than U.S. infants (P=0.064) (Figure 1C). " If the difference is not statisticaly significant, you cannot conclude that there is a difference. Correct to "Pre-vaccine RV-IgA levels were higher in Peruvian compared to U.S. infants (P<0.001), but similar between Panamanian and both Peruvian and U.S. infants (P=0.064 and P=0.064, respectively) (Figure 1C). "**

Thank you for flagging this error. We have made the following changes to lines 98 to 101 on page 4:

> Pre-vaccine RV-IgA levels were higher in Peruvian compared to U.S. infants (P<0.001) and similar to Panamanian infants (P=0.06). Panamanian infants displayed similar pre-vaccine IgA than U.S. infants (P=0.064).

**7. Reviewer #3 (Remarks on code availability):**

**The code is clear and usable.**
